# Quantitative HILIC-Q-TOF-MS Analysis of Glycosaminoglycans and Non-reducing End Carbohydrate Biomarkers via Glycan Reductive Isotopic Labeling

**DOI:** 10.1021/acs.analchem.5c02338

**Published:** 2025-08-06

**Authors:** Amrita Basu, Stephanie Archer-Hartmann, Pradeep Chopra, Mehrnoush Taherzadeh Ghahfarrokhi, Xiaolin Dong, Neil G. Patel, Yiwen Zhang, Biswa Choudhury, Kosuke Funato, Dhananjay Yellajoshyula, Geert-Jan Boons, Parastoo Azadi, Ryan J. Weiss

**Affiliations:** † Complex Carbohydrate Research Center, 1355 University of Georgia, Athens, Georgia 30602, United States; ‡ Department of Chemistry, University of Georgia, Athens, Georgia 30602, United States; § Department of Biochemistry and Molecular Biology, 1355 University of Georgia, Athens, Georgia 30602, United States; ∥ Department of Neurosciences, School of Medicine, 2546 Case Western Reserve University, Cleveland, Ohio 44106, United States; ⊥ GlycoAnalytics Core, 8784 University of California San Diego, La Jolla, California 92093, United States; # Center for Molecular Medicine, University of Georgia, Athens, Georgia 30602, United States; ∇ Department of Chemical Biology and Drug Discovery, Utrecht Institute for Pharmaceutical Sciences and Bijvoet Center for Biomolecular Research, University of Utrecht, Utrecht 3584 CG, the Netherlands

## Abstract

Glycosaminoglycans (GAGs) are linear, heterogeneous polysaccharides expressed on all animal cells. Sulfated GAGs, including heparan sulfate (HS) and chondroitin/dermatan sulfate (CS/DS), are involved in numerous physiological and pathological processes; therefore, precise and robust analytical methods for their characterization are essential to correlate structure with function. In this study, we developed a method utilizing hydrophilic interaction liquid chromatography coupled with time-of-flight mass spectrometry (HILIC-Q-TOF-MS) and glycan reductive isotopic reducing end labeling (GRIL) for the quantitative compositional analysis of HS and CS/DS polysaccharides. Lyase-generated disaccharides and commercial standards were chemically tagged on the reducing end with aniline stable isotopes, thus enabling the absolute quantification of HS and CS/DS disaccharides in complex biological samples. In addition, we adapted this workflow, in conjunction with new synthetic carbohydrate standards, for the quantification of disease-specific non-reducing end (NRE) carbohydrate biomarkers that accumulate in patients with mucopolysaccharidoses (MPS), a subclass of lysosomal storage disorders. As a proof of concept, we applied this method to measure NRE biomarkers in patient-derived MPS IIIA and MPS IIID fibroblasts, as well as in cortex tissue from a murine model of MPS VII. Overall, this method demonstrates improved sensitivity compared to previous GRIL-LC/MS techniques and, importantly, avoids the use of ion-pairing reagents, which are undesirable in certain mass spectrometry instrumentation and contexts. By combining the benefits of HILIC separation with isotopic labeling, our approach offers a robust and accessible tool for the analysis of GAGs, paving the way for advancements in understanding GAG structure and function.

## Graphical abstract

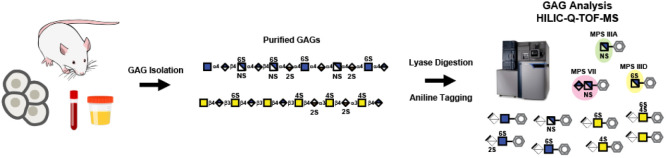

## Introduction

Glycosaminoglycans (GAGs) are essential polysaccharides found abundantly in the extracellular matrix (ECM) and on the cell surface of all animal cells, and they play pivotal roles in various physiological processes such as cell signaling, adhesion, and tissue development. GAG chains are composed of repeating disaccharide units of amino sugars and uronic acids that are sulfated at specific residues, thus creating binding sites for proteins and ligands that impact a diverse array of cellular processes.^1^ Heparan sulfate (HS) and chondroitin/dermatan sulfate (CS/DS) are two major subclasses of GAGs that consist of repeating glucosamine (GlcN) and glucuronic/iduronic acid (GlcA/IdoA) residues or *N*-acetyl-galactosamine (GalNAc) and GlcA/IdoA sugars, respectively (Figure 1A). While CS chains contain repeating disaccharide units of GalNAc and GlcA, dermatan sulfate (DS), also known as CS-B, is characterized by the additional presence of IdoA along with GalNAc and GlcA.^2^ The glucosamine residues of HS can be *N*-acetylated or *N*-sulfated and *O*-sulfated at the C3 and/or C6 positions, while GlcA/IdoA can be 2-*O*-sulfated. CS/DS can be *O*-sulfated at the C4 or C6 position of GalNAc residues and at the C2 position of GlcA/IdoA. These sulfated GAGs are covalently linked to serine residues of core proteins, known as proteoglycans, via a common tetrasaccharide linker (Ser-Xyl-Gal-Gal-GlcA). Genetic mutations in specific HS and CS/DS biosynthetic enzymes, as well as lysosomal enzymes important for GAG catabolism, can result in multiple human disorders, including musculoskeletal, lysosomal storage, and metabolic diseases.^3–5^ Given their significance in cellular physiology and pathophysiology, elucidating the composition, structure, and downstream function of GAGs is essential for understanding relevant biological processes and developing therapeutic interventions for relevant human diseases.

**Figure 1:**
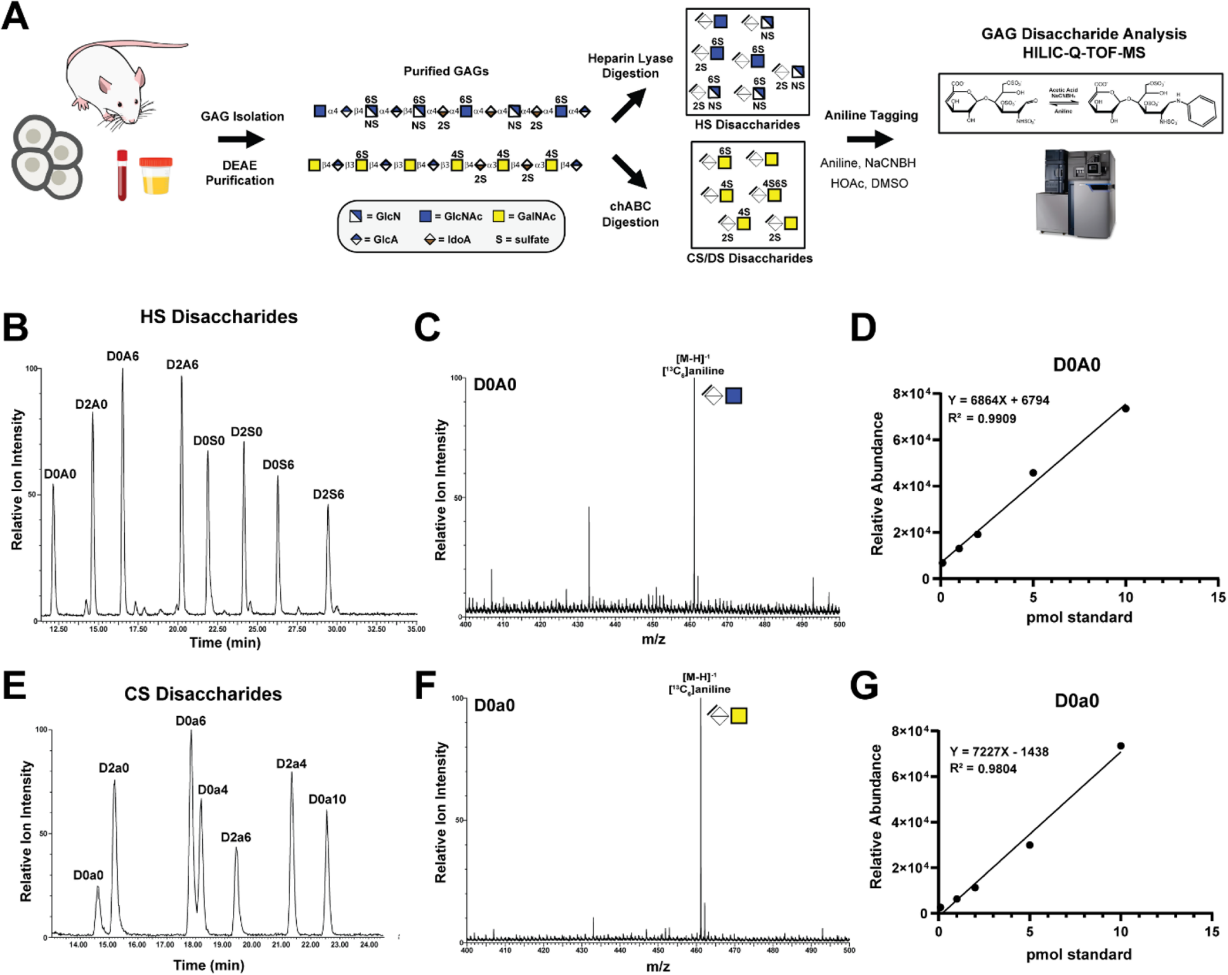
HILIC-Q-TOF-MS method development for disaccharide analysis of glycosaminoglycans. (A) Overview of the workflow for isolation, purification, and reductive isotopic labeling for HILIC-Q-TOF-MS analysis. (B) The extracted ion chromatogram (XIC) for each of a mixture of eight HS disaccharide standards. The disaccharide structure code is described in Table S1 and ref 41. (C) XIC for the free molecular ion for [^13^C_6_] aniline-labeled HS disaccharide, D0A0, [M-H]^−1^ (*m*/*z* = 461). (D) Sensitivity and linear range for HS species (D0A0) shown as a correlation between picomole (pmol) amount and relative mass abundance. (E) The extracted ion chromatogram (XIC) for each of a mixture of seven CS/DS disaccharide standards. (F) XIC for the free molecular ion for [^13^C_6_] aniline-labeled CS/DS disaccharide, D0a0, [M-H]^−1^ (*m*/*z* = 461). (G) Sensitivity and linear range for CS/DS species (D0a0) are shown as a correlation between the picomole amount and relative mass abundance.

Sulfated GAGs, including heparin, HS, and CS/DS, are difficult to study due to their intrinsically heterogeneous polysaccharide backbone, diverse sulfation patterning, and spatiotemporal expression across cell and tissue types.^1,6^ A myriad of analytical techniques has been developed for the structural characterization and quantification of GAGs, each offering unique advantages and limitations. Spectrophotometric methods, such as the carbazole assay^7,8^ and the 1,9-dimethylmethylene blue (DMMB) assay,^9^ provide rapid and cost-effective quantification of total GAG content based on colorimetric detection of uronic acid or sulfated GAGs, respectively. However, these methods lack specificity and cannot discriminate between different GAG subtypes.^10–12^ Chromatographic separation techniques, such as thin-layer chromatography (TLC) and high-performance liquid chromatography (HPLC), enable the determination of GAG disaccharide composition and sulfation patterns, yet they require laborious sample preparation and calibrated standards to provide quantitative results.^13–16^ Overall, mass-spectrometry-based methods provide unparalleled sensitivity and structural information but are often technically challenging and require specific instrumentation and expertise. Therefore, a balance among sensitivity, specificity, throughput, and resource requirements is important to consider when selecting an appropriate analytical method for GAG analysis.

While innovative “top-down” methods are under development to analyze GAG oligosaccharides^17–19^ and fully intact chains,^20–23^ disaccharide analysis remains an essential analytical tool for GAG compositional analysis. To assess sulfated GAG composition via LC-MS, intact chains are typically purified from cells, tissues, or biological fluids via anion-exchange chromatography, followed by subsequent depolymerization into disaccharides using bacterial lyase enzymes.^24^ GAG-specific lyase enzymes are used to cleave the native chains by a β-eliminative mechanism to produce disaccharide subunits with a 4,5-unsaturated uronic acid at the non-reducing end.^25,26^ While lyase digestion eliminates the epimeric configuration at C-5 of the uronic acid (GlcA/IdoA), it preserves the critical *N*-sulfated, *N*-acetylated, and *O*-sulfated modifications in the resulting disaccharides, allowing for detailed compositional analysis. The depolymerization products can be derivatized with fluorophores (e.g., AMAC)^27^ or isotopic species (e.g., [^13^C_6_] aniline) at the reducing end^28–30^ via reductive amination and analyzed by liquid chromatography techniques and quantitative high-resolution mass spectrometry.

Conventional LC/MS analysis of GAG disaccharides faces limitations in quantitative analysis due to inherent structural variations among glycan species and external factors, such as solvent effects and contaminants, that influence ionization efficiency.^11^ To address these quantitative shortcomings, researchers have adapted glycan reductive isotope labeling (GRIL) with liquid chromatography and mass spectrometry, known as GRIL-LC/MS, to enable both the separation and quantification of heparan sulfate (HS) and chondroitin/dermatan sulfate (CS/DS) disaccharides.^29,31^ While this is a useful method, it is important to note that this approach utilizes a basic ion-pairing agent (e.g., dibutylamine), which interacts with anionic GAG disaccharides to form a neutral ion pair that can interact effectively with the hydrophobic stationary phase but can, unfortunately, contaminate both the LC and MS instrumentation.^32^ As a result, this method has not been widely adopted, mainly due to limited mass spectrometry resources and/or instrumentation that cannot be solely dedicated to these analyses. Hydrophilic interaction liquid chromatography coupled with mass spectrometry (HILIC-LC/MS) offers a powerful solution for these challenges. HILIC is a chromatographic technique that separates polar and hydrophilic compounds based on their differential interactions with a hydrophilic stationary phase and a mobile phase containing a high percentage of organic solvent and a small amount of water. Analytes are retained primarily by partitioning into a thin layer of water on the surface of the stationary phase, making it particularly suitable for the separation of hydrophilic molecules, such as GAGs.^17,33,34^

Previously developed HILIC LC-MS methods for GAG characterization suffer from low sensitivity,^35^ relative quantification, and limited resolution of isobaric species,^14,36,37^ which inhibit the complete characterization of these heterogeneous biomolecules in complex biological samples. In this study, we adapted the GRIL-LC/MS methodology to HILIC coupled with quadrupole time-of-flight mass spectrometry (HILIC-Q-TOF-MS) to enable robust analysis of GAG species with increased sensitivity and without the need for ion-pairing reagents. Here, we show the utilization of this method to analyze commercial and pharmaceutical GAG standards, including anticoagulant heparin as well as HS and CS/DS polysaccharides isolated from murine tissues, human cells, and urine. Importantly, we expanded this method to enable simultaneous quantification of glycosaminoglycan disaccharide profiles and disease biomarker non-reducing end (NRE) species in a single LC-MS run, using patient-derived fibroblasts from Sanfilippo Syndrome patients (MPS IIIA/D) and cortex from a mouse model of Sly Syndrome (MPS VII). New synthetic NRE standards, in conjunction with our improved HILIC-Q-TOF-MS disaccharide method, provide powerful tools to analyze GAGs from biological samples and quantify biomarkers in relevant human disorders.

## Experimental Section

### Materials

HS and CS/DS disaccharides were purchased from Iduron. Disaccharide stock solutions (50 μM) were prepared by dissolving HS or CS/DS standards in MS-grade water at equimolar concentrations and stored in aliquots at −20 °C. Heparin was obtained from Scientific Protein Laboratories (SPL), and heparan sulfate purified from CHO-S (rHS01) was obtained from TEGA Therapeutics Inc. Chondroitin sulfate A sodium salt from bovine trachea (CS-A) was obtained from Sigma (#C9819). *N*-sulfo-glucosamine sodium salt (S0) was obtained from Selleck Chemicals.

### Cell Lines and Cell Culture

A375 (ATCC, CRL-1619) and TC28a2 (Millipore Sigma, SCC042) cells were grown in Dulbecco’s Modified Eagle Medium (DMEM; Gibco) supplemented with 10% (v/v) FBS and 1% (v/v) penicillin/streptomycin at 37 °C under an atmosphere of 5% CO_2_/95% air. Cells were subcultured every 2–4 days, and fresh cell stocks were revived from liquid nitrogen after ≤10 passages. Human dermal fibroblasts derived from patient biopsies were obtained from the Coriell Institute (Camden, New Jersey) for two healthy individuals (GM01652 and AG02602) and MPS IIIA (GM06110) and MPS IIID (GM05093) patients. Fibroblasts were cultured in Dulbecco’s Modified Eagle Medium (DMEM; Gibco) supplemented with 15% (v/v) FBS and 1% (v/v) penicillin/streptomycin at 37 °C under an atmosphere of 5% CO_2_/95% air. For analysis of lysosomal accumulation of heparan sulfate, fibroblasts were seeded on 15 cm tissue culture plates, grown to confluence, and maintained with routine media changes every 4 days for 4 weeks to ensure intracellular bioaccumulation of GAGs for downstream analyses.

### Tissues and Biospecimens

All procedures on animals were approved by the Institutional Animal Care and Use Committee (IACUC) of the University of Georgia, and protocols were performed in accordance with the Guide for the Care and Use of Laboratory Animals published by the National Institute of Health (NIH). Male and female NOD-SCID mice (Jackson Laboratories) were sacrificed under a protocol approved by the Institutional Animal Care and Use Committee (IACUC) of the University of Georgia. The brain and liver were isolated, washed multiple times with cold PBS (phosphate-buffered saline), and lyophilized overnight. The lyophilized tissue samples were preweighed, homogenized in wash buffer (50 mM sodium acetate buffer, 200 mM NaCl, pH 6.0), and subsequently used for GAG isolation, as previously described.^24^ Human urine samples, without personal identifying information, were obtained from healthy donors with documented informed consent and IRB approval from the University of Georgia. Urine samples were centrifuged at 2000 rpm prior to GAG isolation.

MPS VII mice (ByBir-Gusbmps/J) carrying a null *Gusb* (*Gusb* ^mps^) allele^38^ were obtained from Jackson Laboratories and were housed in Association for Assessment and Accreditation of Laboratory Animal Care at Case Western Reserve University. Total GAGs were isolated from cortical tissue dissected from brains after PBS perfusion of 2-month-old mutant (*Gusb* ^*mps/mps*^) and littermate control (*Gusb* ^*+/+*^) mice.

### GAG Isolation and Purification from Mammalian Cells

Cell surface and intracellular GAGs were isolated and purified from cells, as previously reported.^24,39^ Briefly, cells were washed twice with PBS, lifted with trypsin (Gibco), and the trypsin fraction (cell surface GAGs) was separated from the cell pellet (intracellular GAGs) via centrifugation (1000 rpm, 5 min). Cell pellets were washed with cold PBS and then lysed using 0.5% CHAPS lysis buffer (50 mM HEPES, 120 mM NaCl, 2 mM EDTA, pH 7.4) containing a protease inhibitor cocktail (Roche). 50 μL of cell lysate was set aside for protein quantification via BCA assay. The trypsin-released or intracellular GAG fractions were diluted 1:10 in wash buffer (50 mM sodium acetate, 200 mM NaCl, and 0.1% Triton X-100, pH 6.0) and incubated with Pronase (0.4 mg/mL, Sigma) overnight at 37 °C with mild agitation. The product was centrifuged (4000*g*, 20 min), then passed through a DEAE-Sephacel (Cytiva) column equilibrated in 50 mM sodium acetate buffer, pH 6.0, containing 200 mM NaCl, and subsequently passed through a PD-10 desalting column (Cytiva).

### GAG Depolymerization and HILIC-Q-TOF-MS Disaccharide Analysis

For HS disaccharide analysis, lyophilized GAGs were incubated with 2 mU each of heparin lyases I, II, and III (IBEX) for 16 h at 37 °C in a buffer containing 40 mM ammonium acetate and 3.3 mM calcium acetate, pH 7. For CS/DS disaccharide analysis, lyophilized GAGs were incubated with 2 mU of chondroitinase ABC (Sigma) for 16 h at 37 °C in a buffer containing 50 mM Tris and 50 mM NaCl, pH 7.9. HS and CS/DS disaccharides were aniline-tagged, as previously described.^29^ HILIC-UPLC was performed on a Waters Acquity UPLC system (Waters Corporation, Milford, MA) equipped with a binary solvent manager, sample manager, fluorescence detector, and column manager. Separation was achieved on a Waters Acquity UPLC Amide BEH column (2.1 mm × 150 mm, 1.7 μm) maintained at 40 °C. Mobile phases consisted of (A) acetonitrile and (B) 50 mM ammonium formate in water at pH 4.4 (Ammonium Formate Solution – Glycan Analysis, Waters). For HS analyses, the following gradient program was used: 0–5 min, 90% A; 5–48 min, 90–67% A; 48–60 min, 67% A; 60–65 min, 67–90% A; and 65–70 min, 90% A. For the mixture of MPS IIIA NRE (S0) and HS disaccharides, the following gradient was used: 0–5 min, 92% A; 5–48 min, 92–67% A; 48–49 min, 67% A; 49–51.5 min, 67–65% A; 51.5–60 min, 65% A; 60–62 min, 65–92% A; and 62–70 min, 92% A. For CS/DS analyses, the following gradient program was used: 0–5 min, 94–92% A; 5–8 min, 92–90% A; 8–28 min, 90–78% A; 28–30 min, 78–75% A; 30–37 min, 75–94% A; and 37–40 min, 94% A. The flow rate was 0.5 mL/min in all runs. The injection volume was 1–2 μL. The UHPLC system was coupled to a Waters Synapt XS Q-TOF mass spectrometer equipped with an electrospray ionization source operated in negative ion mode. The following source parameters were used: source temperature 80 °C, desolvation temperature 250 °C, cone gas flow 50 L/h, desolvation gas flow 1000 L/h, capillary voltage 2.0 kV, sampling cone 35 V, and source offset 4.0 V. Data were acquired in resolution mode from 200 to 1000 *m*/*z*. For the MPS IIIA NRE runs, an isolated 10 min window in the beginning of the run was performed at targeted enhancement at the 338 *m*/*z* value for increased sensitivity for the NRE monosaccharide ions. MassLynx software (Waters Corporation) was used for molecular feature extraction and data processing. Total GAGs were normalized to protein amount measured via BCA assay (cells), sample volume (urine), or dry weight (lyophilized tissue).

### Non-Reducing End (NRE) Analysis

Cell lysates from aged patient fibroblasts or homogenized brain cortex from MPS VII mice were used for NRE analyses. GAGs were isolated as described above, subsequently enzymatically depolymerized with heparin lyases, and differentially mass-labeled by reductive amination with [^12^C_6_] aniline, as described above. Each sample was then mixed with [^1 3^C_6_] aniline-tagged *N*-sulfo-glucosamine (S0), glucosamine-6-sulfate (H6), or β-d-glucopyranosyluronate-(1–4)-*O*-2-deoxy-2-*N*-sulfamino-α/β-d-glucopyranose (G0S0) standards (10 pmol), respectively. The injection volume was 1.0 μL. See the Supporting Information for synthetic schemes and chemical characterization of synthetic standards. Samples were analyzed by HILIC-Q-TOF-MS, as described above. NREs were quantified using the isotopically labeled internal standard and normalized to total protein, as measured by BCA, or tissue weight.

## Results and Discussion

### Isotopic Labeling and HILIC-Q-TOF-MS Enable Separation and Sensitive Detection of GAG Disaccharides

The goal of this study was to establish a robust and accessible method for the absolute quantification of GAGs across diverse biological samples, including cells, tissues, and biofluids. To achieve this, we aimed to advance and optimize the GRIL-LC-MS technique to enable the separation and quantification of HS and CS/DS disaccharides labeled with distinct isotopic aniline tags (^12^C_6_ and ^13^C_6_) using hydrophilic interaction liquid chromatography (HILIC) coupled with quadrupole time-of-flight (Q-TOF) mass spectrometry (Figure 1A). The use of HILIC eliminates the need for ion-pairing reagents while ensuring high-fidelity separation and detection of GAG species. Optimization of the HILIC method for GRIL-LC/MS was based on the chromatographic behavior of commercial unsaturated HS and CS/DS disaccharide standards. Each equimolar mixture of GAG disaccharides was first subjected to isotopic labeling of [^13^C_6_] aniline via reductive amination following previously published procedures.^29^ This reaction involves incubating standard disaccharides with a 100-fold molar excess of [^13^C_6_] aniline at 37 °C for 16 h, which results in complete conjugation. Next, we developed a chromatography method to separate the aniline-tagged HS or CS/DS disaccharides on a BEH Amide 1.7 μm HILIC column using isocratic and linear gradients of acetonitrile and ammonium formate (NH_4_HCO_2_) over 70- and 40-min runs, respectively (Figure 1B,E, see Experimental Section for details). By gradient optimization, it was possible to fully resolve the mixture of eight HS and seven CS/DS disaccharides, respectively, with baseline separation, with the exception of CS/DS disaccharides D0a4 and D0a6, which gave adequate but incomplete (∼80%) baseline separation (Figure 1E). Aniline tagging gave superior disaccharide separation compared to previous HILIC-MS techniques.^40^ All HS and CS/DS disaccharide standards resolved with retention times based on the degree of sulfation and disaccharide identity and are presented as extracted ion chromatograms (XICs). Overall, the *N*-acetylated standards eluted earlier than the *N*-sulfated HS disaccharides. The nonsulfated D0A0 (ΔUA-GlcNAc) and D0a0 (ΔUA-GalNAc) disaccharides (461 *m*/*z*) eluted earliest at the lowest ammonium formate concentration (Figure 1C,F), while the trisulfated D2S6 (659 *m*/*z*) and disulfated D0a10 (621 *m*/*z*) species eluted last for HS and CS/DS, respectively. While the elution order was similar to prior methods, disaccharide standards were run individually to confirm specific HS and CS/DS disaccharide species and retention times (Figure S1).

To evaluate the linearity and sensitivity of our HILIC-Q-TOF-MS quantification approach, we analyzed varying amounts of HS and CS/DS aniline-labeled disaccharide standards. This method enabled sensitive and reliable detection down to 0.1 picomoles of material, with strong linear correlations (*R* ^2^ > 0.98) between disaccharide concentration and relative mass abundance (Figures 1D,G and S2 and S3), which outperformed previously published methods.^29^ Representative results for D0A0 (ΔUA-GlcNAc) and D0a0 (ΔUA-GalNAc) illustrate the robust and sensitive performance of the method (Figure 1D,G). The expected and detected masses for all [^12^C_6_] and [^13^C_6_] aniline-tagged disaccharides are included in Table S1. The table also includes sodium adducts, which were detected for mono-, di-, and trisulfated species, respectively. Isotopic labeling of disaccharide standards results in a mass shift difference of 6.02 *m*/*z* compared to [^12^C_6_] aniline-labeled samples. We observed nominal sulfate loss only for disulfated CS/DS species (Figure S4), which has been observed in earlier methods;^29,33^ however, this effect was comparable between [^12^C_6_] aniline-labeled samples and [^13^C_6_] aniline-labeled internal standards, ensuring disaccharide quantification without bias. In addition to mass spectrometric detection, we established that aniline fluorescence (285 nm excitation, 345 nm emission) could reliably detect both HS and CS/DS disaccharide species via a fluorescence detector (Figure S5), providing an alternative method for sample detection and analysis.

### HILIC-Q-TOF-MS Analysis of GAG Disaccharides from Commercial Samples

To validate the robustness and applicability of our HILIC-Q-TOF-MS approach, we analyzed commercially available GAG standards, including pharmaceutical heparin (SPL), HS isolated from Chinese hamster ovary (CHO) cells (CHO-S, TEGA Therapeutics), and chondroitin sulfate-A from bovine trachea (CS-A). Each commercial GAG product was enzymatically digested with a mixture of heparin lyases or chondroitinase ABC (chABC), followed by [^12^C_6_] aniline labeling of the resulting disaccharides, as described above. The labeled samples were resuspended in LC-MS-grade water and spiked with [^13^C_6_] aniline-labeled disaccharide standards before subsequent analysis. Upon separation via HILIC chromatography, aniline-labeled disaccharides were clearly resolved and detected based on their expected mass shifts (Figure 2A,D,G). The pharmaceutical heparin disaccharide profile was dominated by highly sulfated species, with D2S6 (trisulfated disaccharide) representing ∼50% of the total disaccharide composition (Figure 2B,C), consistent with previous LC-MS analyses.^42–44^ CHO-derived HS was predominantly nonsulfated (>70%), reflecting the characteristic structural composition of HS from mammalian cell sources^45,46^ ­(Figure 2C,F). For CS-A from bovine trachea, which is primarily 4-*O*-sulfated, our HILIC-Q-TOF-MS method gave an expected distribution of 4-*O*-sulfated (∼70%), 6-*O*-sulfated (∼20%), and nonsulfated (D0a0, ∼10%) disaccharides, similar to prior studies^47^ (Figure 2G–I). Overall, these results show the robustness of this method for the analysis of pharmaceutical and commercial GAG products with diverse compositions.

**Figure 2:**
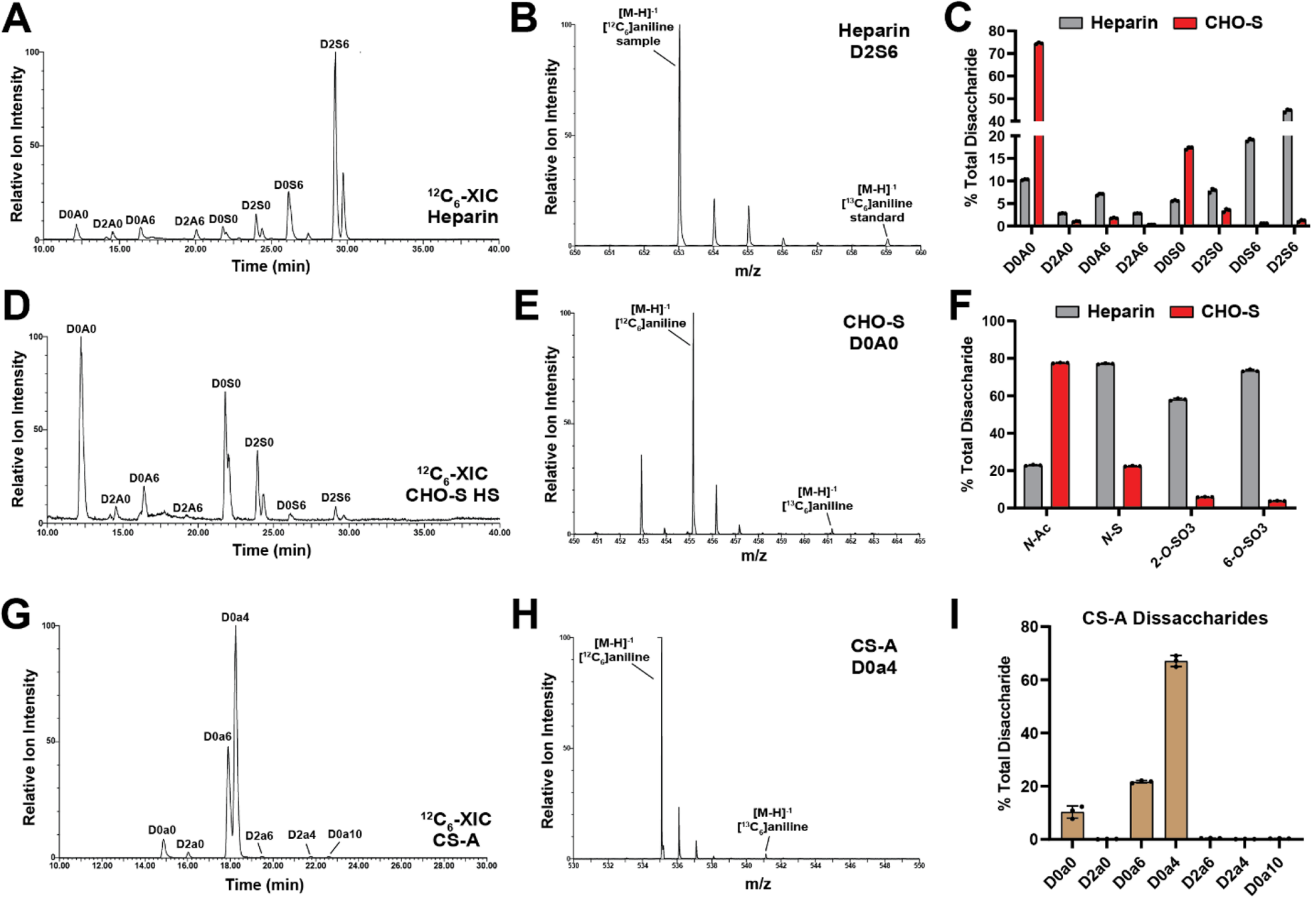
HILIC-Q-TOF-MS analysis of commercial GAG standards. (A) XIC chromatogram for [^12^C_6_] aniline-tagged heparin disaccharides resolved by HILIC-Q-TOF-MS. (B) Corresponding mass spectra for D2S6 from the standard and heparin sample tagged with [^13^C_6_] or [^12^C_6_] aniline, respectively. (C) LC-MS quantification of disaccharides from heparin and CHO-S HS (*n* = 3 biological replicates). (D) XIC chromatogram for [^12^C_6_] aniline-tagged CHO-S HS disaccharides resolved by HILIC-Q-TOF-MS. (E) Corresponding mass spectra for D0A0 from the standard and CHO-S HS sample tagged with [^13^C_6_] or [^12^C_6_] aniline, respectively. (F) Sulfation per disaccharide for pharmaceutical heparin and CHO-S HS (*n* = 3 biological replicates). (G) XIC chromatogram for [^12^C_6_] aniline-tagged CS-A disaccharides resolved by HILIC-Q-TOF-MS. (H) Corresponding mass spectra for D0a0 from the standard and CS-A tagged with [^13^C_6_] or [^12^C_6_] aniline, respectively. (I) LC-MS quantification of disaccharides from CS-A (*n* = 3 biological replicates).

### HILIC-Q-TOF-MS Analysis of GAG Disaccharides from Biological Samples

We next aimed to assess the utility of our HILIC-Q-TOF-MS method using biological samples from cells, tissues, and urine. GAGs were isolated and purified from two distinct human cell lines, A375 melanoma cells and articular chondrocytes (TC28a2), using published methods.^24^ Purified GAGs from each sample were digested with heparin lyases or chABC to yield HS and CS/DS disaccharides, respectively. The disaccharides were tagged with [^12^C_6_] aniline and analyzed via HILIC-Q-TOF-MS (see Figure S6 for XIC chromatograms, Table S3 for raw mass abundances). A375 HS disaccharide analyses revealed ∼60% nonsulfated species, with similar sulfation patterning compared to results using the original GRIL-LC-MS method^39,48^ (Figure 3A,B). A375 cells exhibited similar HS and CS/DS levels (Figure 3C,F), with ∼80% 4-*O*-sulfated CS/DS chains (Figure 3E). TC28a2 chondrocytes exhibited primarily *N*-acetylated and *N*-sulfated HS residues and 4-*O*- and 6-*O*-sulfated CS/DS disaccharides, respectively, with significantly higher total HS versus CS/DS levels (Figure 3C,F). The HILIC-Q-TOF-MS method could distinguish fine compositional differences between diverse human cell types while simultaneously providing absolute quantification of total GAG levels.

**Figure 3:**
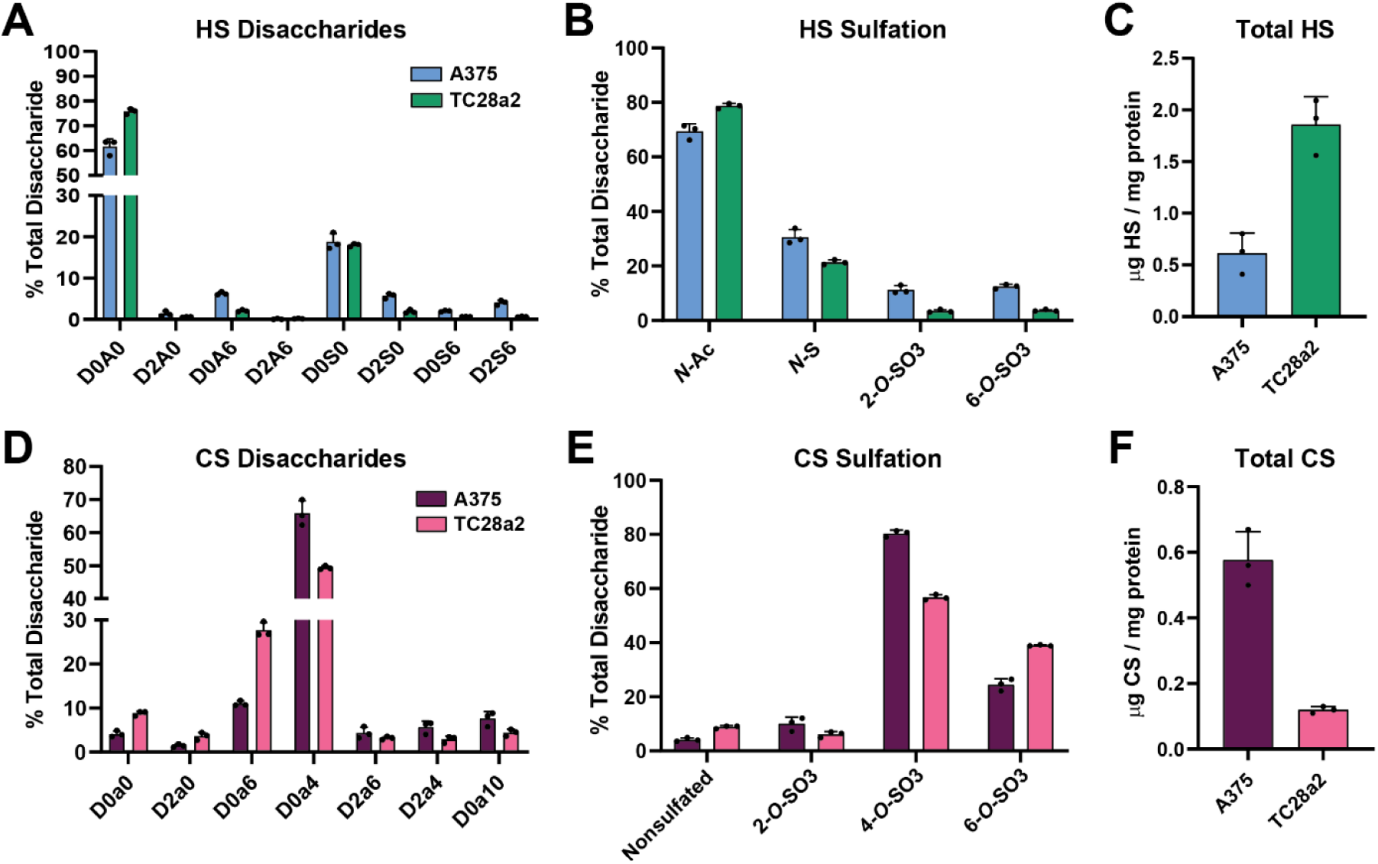
HILIC-Q-TOF-MS analysis of GAGs isolated from human cells. (A) LC-MS quantification of HS disaccharides from human malignant melanoma cells (A375) and human chondrocytes (TC28a2) (*n* = 3 biological replicates). (B) HS sulfation per disaccharide for A375 and TC28a2 cells (*n* = 3). (C) LC-MS quantification of total HS in A375 and TC28a2 cells (*n* = 3). (D) LC-MS quantification of CS/DS disaccharides from A375 and TC28a2 cells (*n* = 3). (E) CS/DS sulfation per disaccharide for A375 and TC28a2 cells (*n* = 3). (F) LC-MS quantification of total CS/DS in A375 and TC28a2 cells (*n* = 3).

We subsequently expanded our method to measure GAGs isolated from tissue, using the liver and brain from NOD-SCID mice as proof of concept. GAGs were purified, and the resultant disaccharides were easily separated and quantified using the HILIC-Q-TOF-MS method, providing comparable yet distinct HS disaccharide profiles for both tissues (Figure 4A,B). The CS/DS composition differed slightly between tissues, with ∼20% 4,6-*O*-sulfated D0a10 species in liver compared with minimal detection in brain samples. Both tissues showed primarily 4-*O*-sulfated (D0a4) chains (Figure 4C,D). Overall, the HS and CS/DS profiles were comparable to prior studies analyzing murine tissues.^49–51^ Finally, we obtained fresh urine samples from healthy donors, purified GAGs, and analyzed the resulting disaccharides. The HILIC-Q-TOF-MS method enabled separation and quantification of HS (Figure 4E,F) and CS/DS (Figure 4G,H) disaccharides from 500 μL of urine, with comparable GAG profiles to prior analyses from a cohort of urine samples from healthy donors.^52^ Overall, these results showcase the versatility, sensitivity, and reproducibility of the disaccharide analysis method using HILIC-Q-TOF-MS with glycan reductive isotope labeling.

**Figure 4:**
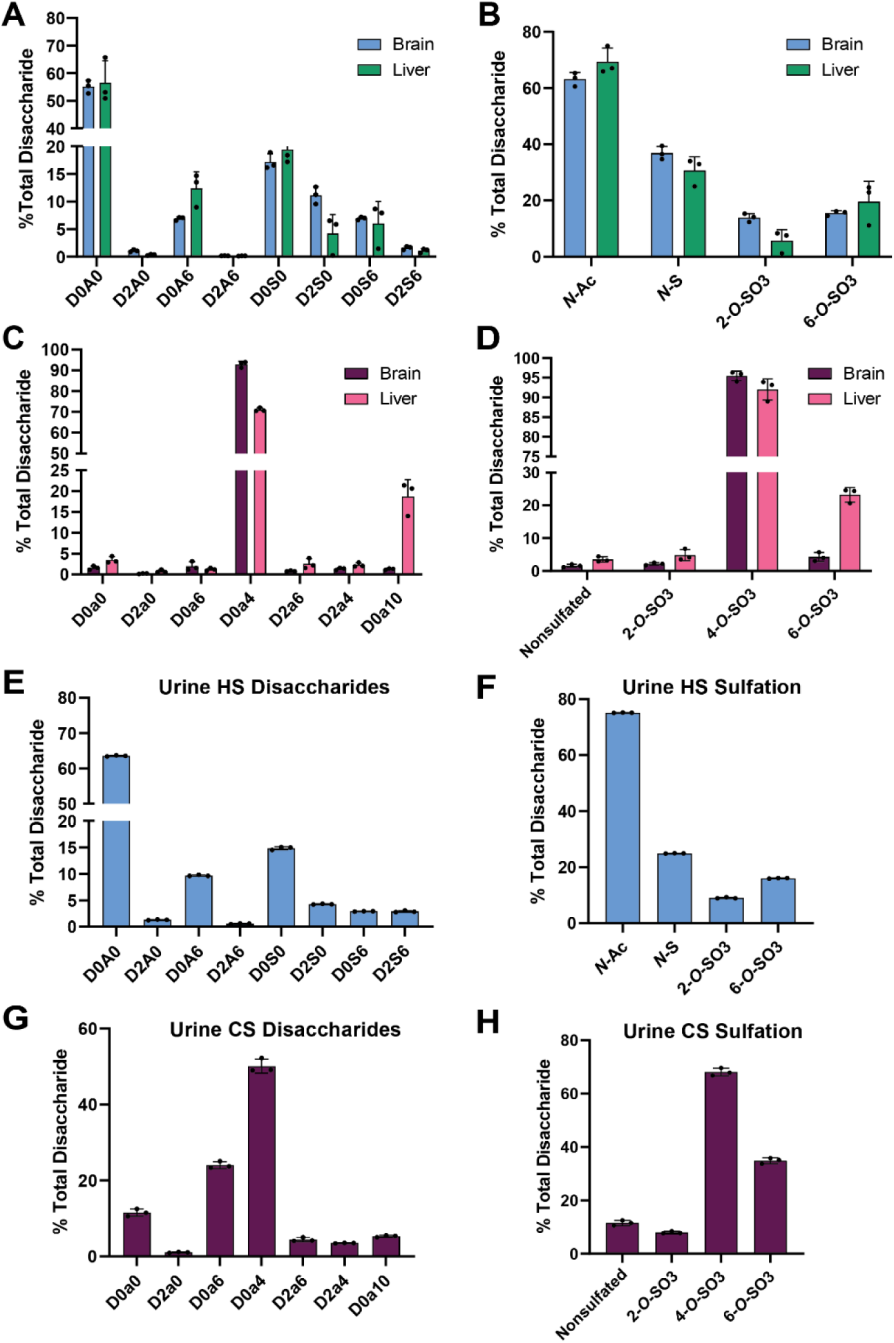
HILIC-Q-TOF-MS analysis of GAGs isolated from murine tissue and human urine. (A) LC-MS quantification of HS disaccharides from NOD-SCID mouse whole brain and liver (*n* = 3 biological replicates). (B) HS sulfation per disaccharide for mouse whole brain and liver (*n* = 3). (C) LC-MS quantification of CS/DS disaccharides from mouse whole brain and liver (*n* = 3). (D) CS/DS sulfation per disaccharide for mouse whole brain and liver (*n* = 3). (E) LC-MS quantification of HS disaccharides from human urine (*n* = 3). (F) HS sulfation per disaccharide for human urine (*n* = 3). (G) LC-MS quantification of CS/DS disaccharides from human urine (*n* = 3). (H) CS/DS sulfation per disaccharide for human urine (*n* = 3).

### Non-reducing End Biomarker Quantification in Disease Models of Mucopolysaccharidoses

Finally, we expanded the application of this method for absolute quantification of non-reducing end (NRE) carbohydrate biomarkers for a subgroup of inherited lysosomal storage disorders known as mucopolysaccharidoses (MPS).^32^ MPS is a group of at least 11 different autosomal recessive disorders caused by loss-of-function mutations in lysosomal enzymes responsible for the catabolism of sulfated GAGs. HS and CS/DS are typically degraded via stepwise removal of monosaccharide residues and sulfate groups from the non-reducing end of the polysaccharide chains in the lysosome. Deficiency in a single lysosomal enzyme leads to the cumulative buildup of undegraded GAGs containing a characteristic terminal NRE carbohydrate structure, which is detectable by LC-MS upon release with enzymatic digestion.^53^ Patients exhibit a variety of progressive, multisystemic symptoms early in childhood, which often lead to early mortality within the first two decades of life.^54^ Sensitive detection and quantification of NRE species are important for patient diagnosis and monitoring therapeutic efficacy.^55^

Currently, the quantitative analysis of NRE biomarkers in MPS patients remains challenging due to the lack of appropriate chemical standards and the need for dedicated instrumentation. To address this, we synthesized new NRE HS standards from respective mono- and disaccharide precursors to enable absolute quantification of characteristic NRE biomarkers for MPS I (I0S0), MPS II (I2S6), MPS IIID (H6), and MPS VII (G0S0) (see Supporting Information for synthetic methods). Each NRE species, including a commercial *N*-sulfo-glucosamine monosaccharide standard for MPS IIIA (S0), was isotopically labeled with aniline, as described above, and suitably detected by HILIC-Q-TOF-MS (Figures 5A,D,G and S7 and Table 2). As a proof of concept, we analyzed total HS levels and NRE biomarkers isolated from MPS IIIA (*N*-sulfo-glucosamine sulfohydrolase [*SGSH*] deficiency)^56^ and MPS IIID (glucosamine-6-sulfatase [*GNS*] deficiency)^57^ patient-derived fibroblasts. Fibroblasts were aged in culture for 4 weeks to ensure lysosomal storage of GAGs, and accumulated intracellular HS and NRE species were isolated, digested with heparin lyases, and analyzed in comparison to healthy control fibroblasts. By spiking in a known amount of isotopically [^13^C_6_] aniline-labeled NRE standards to each sample, we could absolutely quantify disease-specific NRE monosaccharides and total HS via HILIC-Q-TOF-MS (Figure 5). Unique retention times of the NRE standards enabled the simultaneous evaluation of NREs and HS disaccharides. Total HS levels were significantly elevated in both MPS IIIA and IIID fibroblasts (Figure 5B,E), as expected, with a concomitant accumulation of each respective NRE monosaccharide species (Figure 5C,F). Furthermore, for the first time, we could detect and quantify G0S0 NRE disaccharide (Figure 5G) accumulated in brain cortex from an established murine model of MPS VII (β-d-glucuronidase [*GUSB*] deficiency), also known as Sly Syndrome.^58^ MPS VII murine cortex exhibited significant accumulation of intracellular HS (Figure 5H) and G0S0 NRE, respectively (Figure 5I). NRE disaccharide standards for MPS I (α-L-iduronidase [*IDUA*] deficiency) and MPS II (iduronate-2-sulfatase [*IDS*] deficiency) were sufficiently detected using our HILIC-Q-TOF-MS method and will be useful tools for the field (Figure S7). Overall, these results reveal the utility of the HILIC-Q-TOF-MS method to provide a simple and accessible method to quantify GAGs from biological samples and disease biomarkers for diagnosis and treatment efficacy for rare genetic disorders.

**Figure 5:**
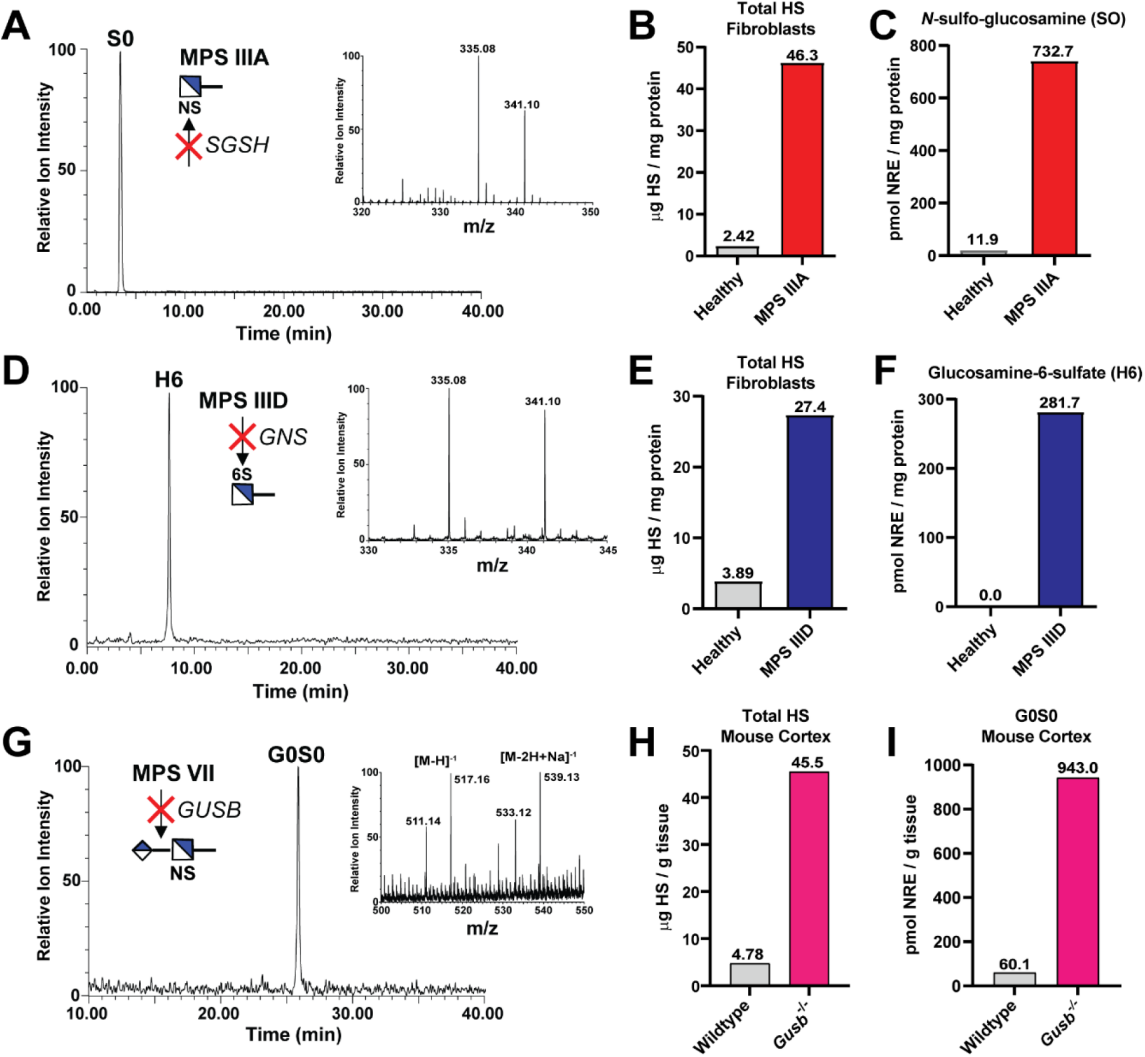
HILIC-Q-TOF-MS analysis of non-reducing end (NRE) species from MPS IIIA/IIID fibroblasts and a mouse model of MPS VII. (A) XIC chromatogram for aniline-tagged *N*-sulfo-glucosamine (S0) monosaccharide resolved by HILIC-Q-TOF-MS. Inset: mass spectra for [^12^C_6_] (335 *m*/*z*) and [^13^C_6_] (341 *m*/*z*) aniline-tagged S0 shown in panel A. (B) LC-MS quantification of total HS levels from aged MPS IIIA patient-derived fibroblasts (GM06110) versus healthy control fibroblasts. (C) Analysis of *N*-sulfo-glucosamine (S0) NRE species in MPS IIIA and healthy fibroblasts. (D) XIC chromatogram for aniline-tagged glucosamine-6-sulfate (H6) monosaccharide resolved by HILIC-Q-TOF-MS. Inset: mass spectrum for [^12^C_6_] (335 *m*/*z*) and [^13^C_6_] (341 *m*/*z*) aniline-tagged H6 shown in panel D. (E) LC-MS quantification of total HS levels from aged MPS IIID patient-derived fibroblasts (GM05093) versus healthy control fibroblasts. (F) Quantification of glucosamine-6-sulfate (H6) NRE species in MPS IIID and healthy fibroblasts. (G) XIC chromatograph for aniline-tagged G0S0 NRE disaccharide resolved by HILIC-Q-TOF-MS. Inset: mass spectra for [^12^C_6_] (511 *m*/*z*) and [^13^C_6_] (517 *m*/*z*) aniline-tagged G0S0 shown in panel G. Respective sodium adducts (533, 539 *m*/*z*) are also included. (H) LC-MS quantification of total HS levels from wild-type and MPS VII mice (*Gusb* ^*–/–*^). (I) Quantification of G0S0 NRE species in wild-type and MPS VII (*Gusb* ^–/–^) mice.

## Method Considerations

The method outlined in this report demonstrates the use of GRIL labeling combined with commercially available chromatography and mass spectrometry. It boasts sensitivity below 1 picomole and has been used to analyze GAG content from a wide variety of starting sources, including commercial and pharmaceutical GAG products, as well as GAGs from biological materials such as urine, cells, and tissue. The method’s utilization of standards labeled with isotopically heavy aniline allows for quantitative analysis of GAG samples with a large variation in *N*- and *O*-sulfation and *N*-acetylation, which greatly impact ionization efficiency and adduct formation. Furthermore, this labeling method can be carried out without extensive purification, which may result in sample loss or bias. However, it should be noted that the use of these standards adds a level of complexity to the method, in that it requires acquisition of both the standards themselves as well as an isotopically heavy tag for reductive amination. It is because both GAG disaccharide standards and heavy aniline are commercially available that the GRIL method is well-suited for GAG disaccharide analysis and broad application.

Because the method utilizes a common reductive amination labeling strategy, its use can be theoretically adapted to a wide variety of other oligosaccharides, including longer oligosaccharides. However, the adaptation of this method to such species would rely on a number of factors, including labeling efficiency, modifications to the chromatography, potential adduct and ionization considerations, and appropriation of suitable standards. While labeling via reductive amination can be carried out on much larger oligosaccharides than the ones we feature in this report, and HILIC chromatography is commonly used for larger oligosaccharides, such as *N*-glycans, the method would be more complicated when applied to larger GAG fragments. From a practical perspective, the consideration of a GAG oligosaccharide, such as a tetrasaccharide, greatly increases the complexity in terms of the chemical standards that would need to be procured, as well as the number of observed *m*/*z* values arising from sodium adducts and/or acetyl or sulfate losses in the spray.

The HILIC-LC/MS method described in this study is device-agnostic and can be utilized on most liquid chromatography–mass spectrometry systems that are equipped with chromatography amenable to HILIC chromatography and a mass spectrometer sensitive enough to detect the analytes of interest. We utilized a BEH amide HILIC column, which is widely sold commercially through a variety of vendors, including other column types. While we laud the method’s ability to detect as low as 0.1 picomoles of material using a Waters Q-TOF, not all studies of GAGs require such low sample amounts, and other mass spectrometer types (including triple quadrupole, ion trap, and Orbitrap) may also be used, albeit with understandable differences in sensitivity. Because the method is highly focused on only a select number of analytes and relies on the use of internal standards (which confirm both retention time and mass), the mass resolution requirements may also be minimized. In short, the instrumentation requirements are based more on the starting amount of material than on the method itself.

## Conclusions

In this study, we have established a robust and sensitive method that combines hydrophilic interaction liquid chromatography, high-resolution time-of-flight mass spectrometry, and glycan reductive isotopic labeling for GAG disaccharide analysis. This workflow achieves exceptional sensitivity and specificity in quantifying both disaccharide profiles and non-reducing end biomarkers from complex biological samples. Notably, the avoidance of ion-pairing reagents addresses long-standing challenges in mass spectrometry workflows, making this approach broadly accessible across diverse analytical contexts. The synthesis of saturated NRE monosaccharide and disaccharide structures importantly enables the detection and absolute quantification of disease-specific biomarkers for mucopolysaccharidoses. The HILIC-Q-TOF-MS method can be applied to urine, blood, and other biological specimens to aid in the diagnosis and therapeutic efficacy assessment for these disorders. Looking ahead, the integration of synthetic standards and advanced chromatographic techniques paves the way for deeper exploration of GAG structure–function relationships, with implications for therapeutic drug discovery. Future studies will focus on expanding this methodology to encompass other GAG oligosaccharides, including 3-*O*-sulfated HS species and additional GAG subclasses (e.g., keratan sulfate), to further optimize this method for biomedical and clinical applications.

## Supplementary Material





## Data Availability

Any data supporting the analyses in the manuscript are available from the corresponding author upon reasonable request. Raw data for LC-MS analysis of GAGs are available at GlycoPOST^59^ under project ID GPST000604.

## Notes

Tables, supplementary figures, synthetic schemes, and details of experimental procedures are provided in the Supporting Information (PDF)
Table S3: Raw mass abundances for disaccharides from all biological samples (XLSX)

## References

[1] Basu A., Patel N. G., Nicholson E. D., Weiss R. J. (2022). Spatiotemporal diversity
and regulation of glycosaminoglycans in cell homeostasis and human
disease. Am. J. Physiol. Cell Physiol..

[2] Trowbridge J. M., Gallo R. L. (2002). Dermatan sulfate: new functions from an old glycosaminoglycan. Glycobiology.

[3] Mizumoto S., Yamada S. (2021). Congenital Disorders
of Deficiency in Glycosaminoglycan
Biosynthesis. Front. Genet..

[4] Mizumoto S., Yamada S., Sugahara K. (2015). Mutations in Biosynthetic
Enzymes
for the Protein Linker Region of Chondroitin/Dermatan/Heparan Sulfate
Cause Skeletal and Skin Dysplasias. BioMed.
Res. Int..

[5] Khan S. A., Nidhi F., Leal A. F., Celik B., Herreño-Pachón A. M., Saikia S., Benincore-Flórez E., Ago Y., Tomatsu S. (2024). Glycosaminoglycans in mucopolysaccharidoses and other
disorders. Adv. Clin Chem..

[6] Esko J. D., Selleck S. B. (2002). Order out of chaos:
Assembly of ligand binding sites
in heparan sulfate. Annu. Rev. Biochem..

[7] Bitter T., Muir H. M. (1962). A modified uronic
acid carbazole reaction. Anal. Biochem..

[8] Cesaretti M., Luppi E., Maccari F., Volpi N. (2003). A 96-well assay for
uronic acid carbazole reaction. Carbohydr. Polym..

[9] Ladner Y. D., Alini M., Armiento A. R. (2023). The Dimethylmethylene Blue Assay
(DMMB) for the Quantification of Sulfated Glycosaminoglycans. Methods Mol. Biol..

[10] Frazier S. B., Roodhouse K. A., Hourcade D. E., Zhang L. (2008). The Quantification
of Glycosaminoglycans: A Comparison of HPLC, Carbazole, and Alcian
Blue Methods. Open Glycosci..

[11] Kubaski F., Osago H., Mason R. W., Yamaguchi S., Kobayashi H., Tsuchiya M., Orii T., Tomatsu S. (2017). Glycosaminoglycans
detection methods: Applications of mass spectrometry. Mol. Genet. Metab..

[12] Barbosa I., Garcia S., Barbier-Chassefière V., Caruelle J.-P., Martelly I., Papy-García D. (2003). Improved and
simple micro assay for
sulfated glycosaminoglycans quantification in biological extracts
and its use in skin and muscle tissue studies. Glycobiology.

[13] Toyoda H., Kinoshita-Toyoda A., Selleck S. B. (2000). Structural analysis of glycosaminoglycans
in *Drosophila* and *Caenorhabditis elegans* and demonstration that *tout-velu*, a *Drosophila* gene related to EXT tumor suppressors, affects heparan sulfate *in vivo*. J. Biol. Chem..

[14] Studelska D. R., Giljum K., McDowell L. M., Zhang L. (2006). Quantification of glycosaminoglycans
by reversed-phase HPLC separation of fluorescent isoindole derivatives. Glycobiology.

[15] Deakin J. A., Lyon M. (2008). A simplified and sensitive fluorescent method for disaccharide analysis
of both heparan sulfate and chondroitin/dermatan sulfates from biological
samples. Glycobiology.

[16] Toyoda H., Kinoshita-Toyoda A., Fox B., Selleck S. B. (2000). Structural analysis
of glycosaminoglycans in animals bearing mutations in sugarless, sulfateless,
and tout-velu. Drosophila homologues of vertebrate genes encoding
glycosaminoglycan biosynthetic enzymes. J. Biol.
Chem..

[17] Wu J., Wei J., Chopra P., Boons G. J., Lin C., Zaia J. (2019). Sequencing
Heparan Sulfate Using HILIC LC-NETD-MS/MS. Anal.
Chem..

[18] Klein D. R., Leach F. E., Amster I. J., Brodbelt J. S. (2019). Structural
Characterization
of Glycosaminoglycan Carbohydrates Using Ultraviolet Photodissociation. Anal. Chem..

[19] Lin L., Liu X., Zhang F., Chi L., Amster I. J., Leach F. E. 3., Xia Q., Linhardt R. J. (2017). Analysis of heparin
oligosaccharides
by capillary electrophoresis-negative-ion electrospray ionization
mass spectrometry. Anal. Bioanal. Chem..

[20] Chi L., Wolff J. J., Laremore T. N., Restaino O. F., Xie J., Schiraldi C., Toida T., Amster I. J., Linhardt R. J. (2008). Structural
analysis of bikunin glycosaminoglycan. J. Am.
Chem. Soc..

[21] Ly M., Leach F. E., Laremore T. N., Toida T., Amster I. J., Linhardt R. J. (2011). The proteoglycan bikunin has a defined
sequence. Nat. Chem. Biol..

[22] Laremore T. N., Leach F. E., Amster I. J., Linhardt R. J. (2011). Electrospray
ionization Fourier transform mass spectrometric analysis of intact
bikunin glycosaminoglycan from normal human plasma. Int. J. Mass Spectrom..

[23] Yu Y., Duan J., Leach F. E., Toida T., Higashi K., Zhang H., Zhang F., Amster I. J., Linhardt R. J. (2017). Sequencing the dermatan sulfate chain of decorin. J. Am. Chem. Soc..

[24] Basu A., Weiss R. J. (2023). Glycosaminoglycan Analysis: Purification, Structural
Profiling, and GAG-Protein Interactions. Methods
Mol. Biol..

[25] Maruyama Y., Nakamichi Y., Itoh T., Mikami B., Hashimoto W., Murata K. (2009). Substrate specificity of streptococcal unsaturated
glucuronyl hydrolases for sulfated glycosaminoglycan. J. Biol. Chem..

[26] Linhardt R. J., Avci F. Y., Toida T., Kim Y. S., Cygler M. (2006). CS lyases:
structure, activity, and applications in analysis and the treatment
of diseases. Adv. Pharmacol..

[27] Volpi N., Galeotti F., Yang B., Linhardt R. J. (2014). Analysis of glycosaminoglycan-derived,
precolumn, 2-aminoacridone-labeled disaccharides with LC-fluorescence
and LC-MS detection. Nat. Protoc..

[28] Chang Y., Yang B., Zhao X., Linhardt R. J. (2012). Analysis of glycosaminoglycan-derived
disaccharides by capillary electrophoresis using laser-induced fluorescence
detection. Anal. Biochem..

[29] Lawrence R., Olson S. K., Steele R. E., Wang L., Warrior R., Cummings R. D., Esko J. D. (2008). Evolutionary
differences in glycosaminoglycan
fine structure detected by quantitative glycan reductive isotope labeling. J. Biol. Chem..

[30] Kitagawa H., Kinoshita A., Sugahara K. (1995). Microanalysis of glycosaminoglycan-derived
disaccharides labeled with the fluorophore 2-aminoacridone by capillary
electrophoresis and high-performance liquid chromatography. Anal. Biochem..

[31] Xia B., Feasley C. L., Sachdev G. P., Smith D. F., Cummings R. D. (2009). Glycan
reductive isotope labeling for quantitative glycomics. Anal. Biochem..

[32] Lawrence R., Brown J. R., Lorey F., Dickson P. I., Crawford B. E., Esko J. D. (2014). Glycan-based biomarkers for mucopolysaccharidoses. Mol. Genet. Metab..

[33] Gill V. L., Aich U., Rao S., Pohl C., Zaia J. (2013). Disaccharide
analysis of glycosaminoglycans using hydrophilic interaction chromatography
and mass spectrometry. Anal. Chem..

[34] Staples G.
O., Naimy H., Yin H., Kileen K., Kraiczek K., Costello C. E., Zaia J. (2010). Improved hydrophilic
interaction
chromatography LC/MS of heparinoids using a chip with postcolumn makeup
flow. Anal. Chem..

[35] Staples G. O., Bowman M. J., Costello C. E., Hitchcock A. M., Lau J. M., Leymarie N., Miller C., Naimy H., Shi X., Zaia J. (2009). A chip-based amide-HILIC
LC/MS platform for glycosaminoglycan
glycomics profiling. Proteomics.

[36] Lu H., McDowell L. M., Studelska D. R., Zhang L. (2010). Glycosaminoglycans
in Human and Bovine Serum: Detection of Twenty-Four Heparan Sulfate
and Chondroitin Sulfate Motifs Including a Novel Sialic Acid-modified
Chondroitin Sulfate Linkage Hexasaccharide. Glycobiol. Insights.

[37] Takegawa Y., Araki K., Fujitani N., Furukawa J., Sugiyama H., Sakai H., Shinohara Y. (2011). Simultaneous analysis of heparan
sulfate, chondroitin/dermatan sulfates, and hyaluronan disaccharides
by glycoblotting-assisted sample preparation followed by single-step
zwitter-ionic-hydrophilic interaction chromatography. Anal. Chem..

[38] Sands M. S., Birkenmeier E. H. (1993). A single-base-pair
deletion in the beta-glucuronidase
gene accounts for the phenotype of murine mucopolysaccharidosis type
VII. Proc. Natl. Acad. Sci. U. S. A..

[39] Basu A., Champagne R. N., Patel N. G., Nicholson E. D., Weiss R. J. (2023). TFCP2 is a transcriptional regulator of heparan sulfate
assembly and melanoma cell growth. J. Biol.
Chem..

[40] Ouyang Y., Wu C., Sun X., Liu J., Linhardt R. J., Zhang Z. (2016). Development
of hydrophilic interaction chromatography with quadruple time-of-flight
mass spectrometry for heparin and low molecular weight heparin disaccharide
analysis. Rapid Commun. Mass Spectrom..

[41] Lawrence R., Lu H., Rosenberg R. D., Esko J. D., Zhang L. (2008). Disaccharide structure
code for the easy representation of constituent oligosaccharides from
glycosaminoglycans. Nat. Methods.

[42] Warda M., Gouda E. M., Toida T., Chi L., Linhardt R. J. (2003). Isolation
and characterization of raw heparin from dromedary intestine: evaluation
of a new source of pharmaceutical heparin. Comp.
Biochem. Physiol., Part C: toxicol. Pharmacol..

[43] Thacker B. E., Thorne K. J., Cartwright C., Park J., Glass K., Chea A., Kellman B. P., Lewis N. E., Wang Z., Di Nardo A. (2022). Multiplex
genome editing of mammalian cells
for producing recombinant heparin. Metab. Eng..

[44] Fu L., Li G., Yang B., Onishi A., Li L., Sun P., Zhang F., Linhardt R. J. (2013). Structural characterization of pharmaceutical
heparins prepared from different animal tissues. J. Pharm. Sci..

[45] Datta P., Li G., Yang B., Zhao X., Baik J. Y., Gemmill T. R., Sharfstein S. T., Linhardt R. J. (2013). Bioengineered Chinese Hamster Ovary
cells with Golgi-targeted 3-O-sulfotransferase-1 biosynthesize heparan
sulfate with an antithrombin-binding site. J.
Biol. Chem..

[46] Bame K. J., Esko J. D. (1989). Undersulfated heparan sulfate in a Chinese hamster
ovary cell mutant defective in heparan sulfate N-sulfotransferase. J. Biol. Chem..

[47] Muthusamy A., Achur R. N., Valiyaveettil M., Madhunapantula S. V., Kakizaki I., Bhavanandan V. P., Gowda C. D. (2004). Structural characterization
of the bovine tracheal chondroitin sulfate chains and binding of Plasmodium
falciparum–infected erythrocytes. Glycobiology.

[48] Weiss R. J., Spahn P. N., Chiang A. W. T., Liu Q., Li J., Hamill K. M., Rother S., Clausen T. M., Hoeksema M. A., Timm B. M. (2021). Genome-wide screens uncover KDM2B as a modifier of
protein binding to heparan sulfate. Nat. Chem.
Biol..

[49] Tykesson E., Eriksson M., Li J.-P., Maccarana M. (2025). Disaccharide
Analysis of Glycosaminoglycans From Twenty-Four Organs of Young and
Aged Mice. Proteoglycan Res..

[50] Warda M., Toida T., Zhang F., Sun P., Munoz E., Xie J., Linhardt R. J. (2006). Isolation and characterization
of heparan sulfate from
various murine tissues. Glycoconj. J..

[51] Nagamine S., Tamba M., Ishimine H., Araki K., Shiomi K., Okada T., Ohto T., Kunita S., Takahashi S., Wismans R. G. (2012). Organ-specific Sulfation
Patterns of Heparan
Sulfate Generated by Extracellular Sulfatases Sulf1 and Sulf2 in Mice. J. Biol. Chem..

[52] Han X., Sanderson P., Nesheiwat S., Lin L., Yu Y., Zhang F., Amster I. J., Linhardt R. J. (2020). Structural analysis
of urinary glycosaminoglycans from healthy human subjects. Glycobiology.

[53] Lawrence R., Brown J. R., Al-Mafraji K., Lamanna W. C., Beitel J. R., Boons G. J., Esko J. D., Crawford B. E. (2012). Disease-specific
non-reducing end carbohydrate biomarkers for mucopolysaccharidoses. Nat. Chem. Biol..

[54] Penon-Portmann M., Blair D. R., Harmatz P. (2023). Current and
new therapies for mucopolysaccharidoses. Pediatr.
Neonatol..

[55] Vera M. U., Le S. Q., Victoroff A., Passage M. B., Brown J. R., Crawford B. E., Polgreen L. E., Chen A. H., Dickson P. I. (2020). Evaluation
of non-reducing end pathologic glycosaminoglycan detection method
for monitoring therapeutic response to enzyme replacement therapy
in human mucopolysaccharidosis I. Mol. Genet.
Metab..

[56] Seker
Yilmaz B., Davison J., Jones S. A., Baruteau J. (2021). Novel therapies
for mucopolysaccharidosis type III. J. Inherited
Metab. Dis..

[57] Roca C., Motas S., Marco S., Ribera A., Sanchez V., Sanchez X., Bertolin J., Leon X., Perez J., Garcia M. (2017). Disease correction by AAV-mediated gene therapy in
a new mouse model of mucopolysaccharidosis type IIID. Hum. Mol. Genet..

[58] Grant C. L., López-Valdez J., Marsden D., Ezgü F. (2024). Mucopolysaccharidosis
type VII (Sly syndrome) - What do we know?. Mol. Genet. Metab..

[59] Watanabe Y., Aoki-Kinoshita K. F., Ishihama Y., Okuda S. (2021). GlycoPOST
realizes
FAIR principles for glycomics mass spectrometry data. Nucleic Acids Res..

