# Supplementary material for: Quantitative HILIC-Q-TOF-MS Analysis of Glycosaminoglycans and Non-reducing End Carbohydrate Biomarkers via Glycan Reductive Isotopic Labeling
Source: Anal Chem. 2025 Aug 6;97(32):17490–500. doi: 10.1021/acs.analchem.5c02338 (PMC12368834; doi:10.1021/acs.analchem.5c02338)

SUPPLEMENTARY INFORMATION

Quantitative HILIC-Q-TOF-MS analysis of glycosaminoglycans and non-reducing end carbohydrate biomarkers via glycan reductive isotopic labeling

Amrita Basu[1], Stephanie Archer-Hartmann[1], Pradeep Chopra[1], Mehrnoush Taherzadeh Ghahfarrokhi[1,2], Xiaolin Dong[1,3], Neil G. Patel[1,3], Yiwen Zhang[4], Biswa Choudhury[5], Kosuke Funato[3,6], Dhananjay Yellajoshyula[4], Geert-Jan Boons[1,2,7], Parastoo Azadi[1], Ryan J. Weiss[1,3,*]

[1]Complex Carbohydrate Research Center, University of Georgia, Athens, Georgia 30602, United States

[2]Department of Chemistry, University of Georgia, Athens, Georgia 30602, United States

[3]Department of Biochemistry and Molecular Biology, University of Georgia, Athens, Georgia 30602, United States

[4]Department of Neurosciences, School of Medicine, Case Western Reserve University, Cleveland, Ohio 44106, United States

[5]GlycoAnalytics Core, University of California San Diego, La Jolla, California 92093, United States

[6]Center for Molecular Medicine, University of Georgia, Athens, Georgia 30602, United States

[7]Department of Chemical Biology and Drug Discovery, Utrecht Institute for Pharmaceutical Sciences and Bijvoet Center for Biomolecular Research, University of Utrecht, Utrecht, 3584 CG, The Netherlands

*Corresponding author email address: ryan.weiss@uga.edu

This document contains supplemental methods and information as pertains to all ions identified for GAGs, their putative assignments, and retention times. In addition, synthetic schemes for non-reducing end standards and their characterization are included.

Table of Contents

Supplementary Methods……...……………………………………………………….S3

Supplementary Figure 1…………………………………………………………….S4

Supplementary Figure 2…………………………………………………………….S5

Supplementary Figure 3…………………………………………………………….S6

Supplementary Figure 4…………………………………………………………….S7

Supplementary Figure 5…………………………………………………………….S8

Supplementary Figure 6…………………………………………………………….S9

Supplementary Figure 7…………………………………………………………….S10

Supplementary Tables 1-2.………………………………………………………….S11

General synthetic methods………………………………………………………….S12-26

NMR spectra for synthetic standards……………………………………………….S27-38

Supplementary Methods:

Sample preparation and quantification of total GAG and disaccharide composition

To calculate the amount of each disaccharide or NRE species, peak areas are extracted from XIC chromatograms for each carbohydrate species, representing the ion intensities corresponding to individual [$^{12}C_6$] and [$^{13}C_6$] aniline-tagged heparan sulfate or chondroitin/dermatan sulfate species. Because the [$^{13}C_6$] aniline-tagged standards are added at known amounts and behave in the same way as the corresponding [$^{12}C_6$] aniline-tagged disaccharides, the absolute amount of these residues can be calculated from the ratio of the XIC profiles of each isotope (expressed in picomoles). To back-calculate the actual amount of each disaccharide in a given sample, picomole values are scaled to account for the i) proportion of the digest analyzed, ii) the original sample volume, and iii) injection volume. It is recommended to take a defined proportion of the enzymatically digested/aniline-tagged material for subsequent HILIC-Q-TOF-MS analysis. Typically, 50% for cell-derived samples or 25% for tissue-derived samples is digested and tagged, but this is dependent on cell number, tissue size, and GAG content.

$$\textbf{Amount}_{\textbf{pmol}} = ([^{13}C_6] \text{ peak area} / [^{12}C_6] \text{ peak area}) \times \text{pmol of } [^{13}C_6] \text{ standard injected}$$

$$\textbf{Injected}_{\textbf{pmol}} = \text{Amount}_{\text{pmol}} \times (\text{Volume}_{\text{injected}} / \text{Volume}_{\text{total in vial}})$$

$$\textbf{Injected}_{\textbf{ng}} = \text{Injected}_{\text{pmol}} \times (\text{MW of carbohydrate species} / 1000)$$

$$\textbf{Total in vial (µg)} = [\text{Sum of Injected}_{\text{ng}} / (\text{Volume}_{\text{added to vial}} / \text{Volume}_{\text{total in vial}})] / 1000$$

$$\textbf{Total in sample (µg)} = \text{Total in vial} \times (100 / \% \text{ digested}) \times (\text{Volume}_{\text{digest}} / \text{Volume}_{\text{added in vial}})$$

Total HS or CS content is calculated by summing the picomole values of all detected disaccharides. Results are expressed as absolute mass (µg) normalized to tissue wet weight or protein concentration (µg HS per mg tissue/protein). Disaccharide compositional analysis is expressed as the relative molar contributions (%) of species with distinct sulfation patterns, including *N*-sulfation (*N*-S), *N*-acetylation (*N*-Ac), and *O*-sulfation (e.g., 2-*O*, 4-*O*, 6-*O* positions).

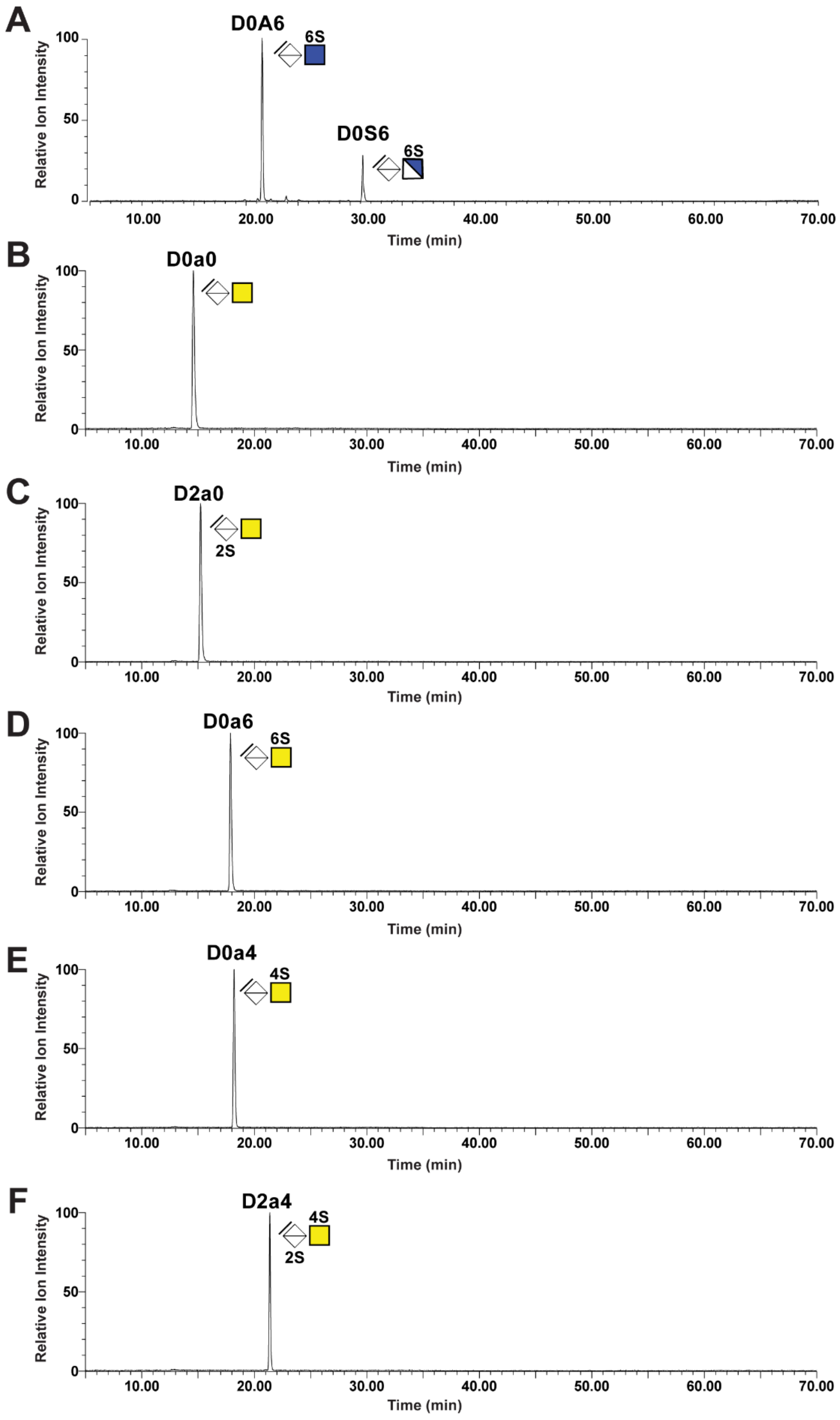

Supplemental Figure 1. Individual disaccharide runs with validated elution times. (A) XIC chromatograms for a mixture of [^{13}C] aniline-tagged D0A6/D0S6 HS disaccharide standards. XIC chromatograms of (B) D0a0, (C) D2a0, (D) D0a6, (E) D0a4, and (F) D2a4 CS/DS disaccharide standards, respectively.

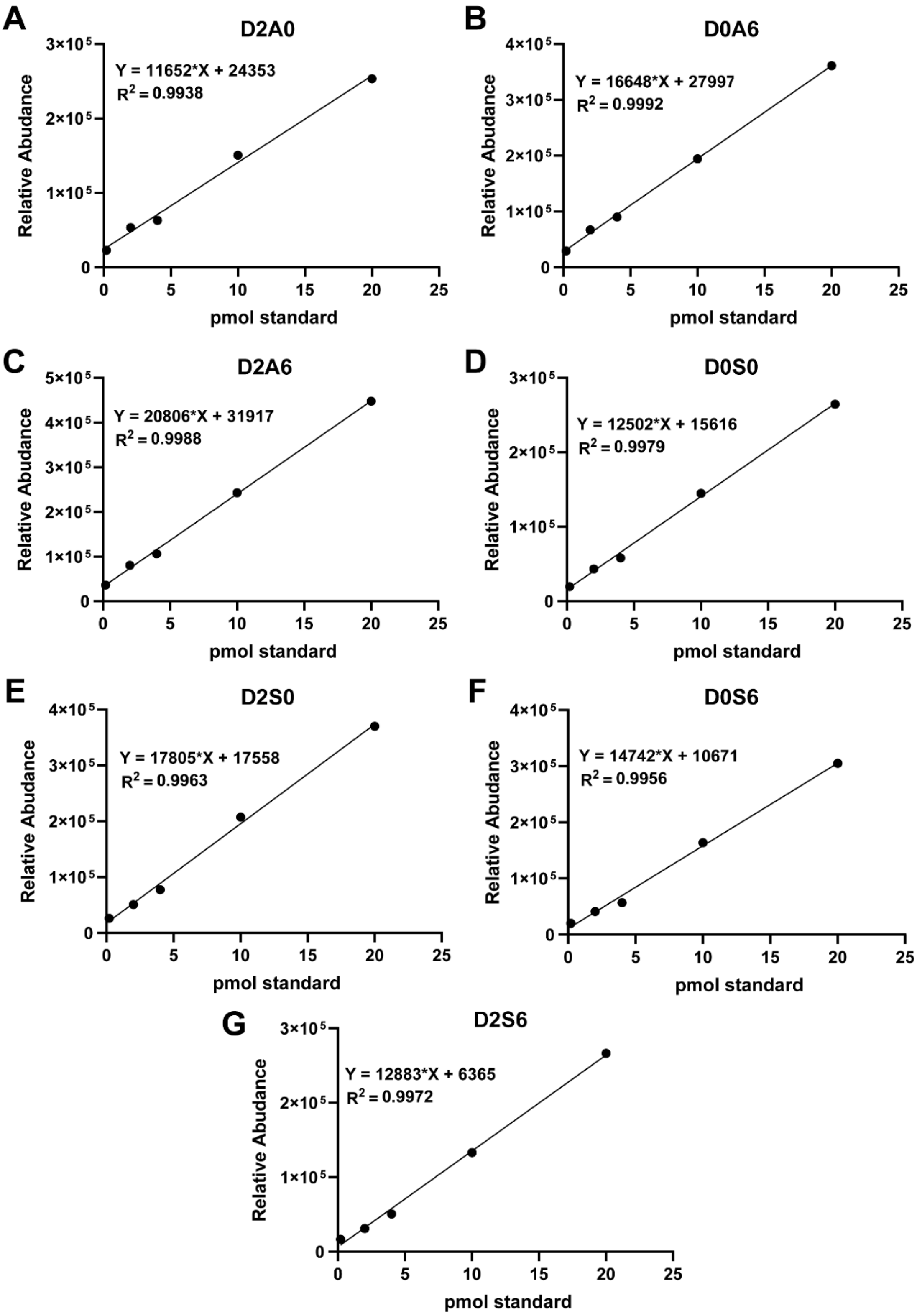

Supplemental Figure 2. Sensitivity and linear range for HS disaccharides. Dynamic range of the HILIC-UPLC/MS analysis for [^{13}C] aniline-tagged (A) D2A0, (B) D0A6, (C) D2A6, (D) D0S0, (E) D2S0, (F) D0S6, and (G) D2S6 disaccharide standards. Disaccharide standards were mixed at equimolar concentrations, injected at various picomole amounts, and followed by HILIC-Q-TOF-MS analysis. The relative abundance at each disaccharide is plotted versus picomole amount injected.

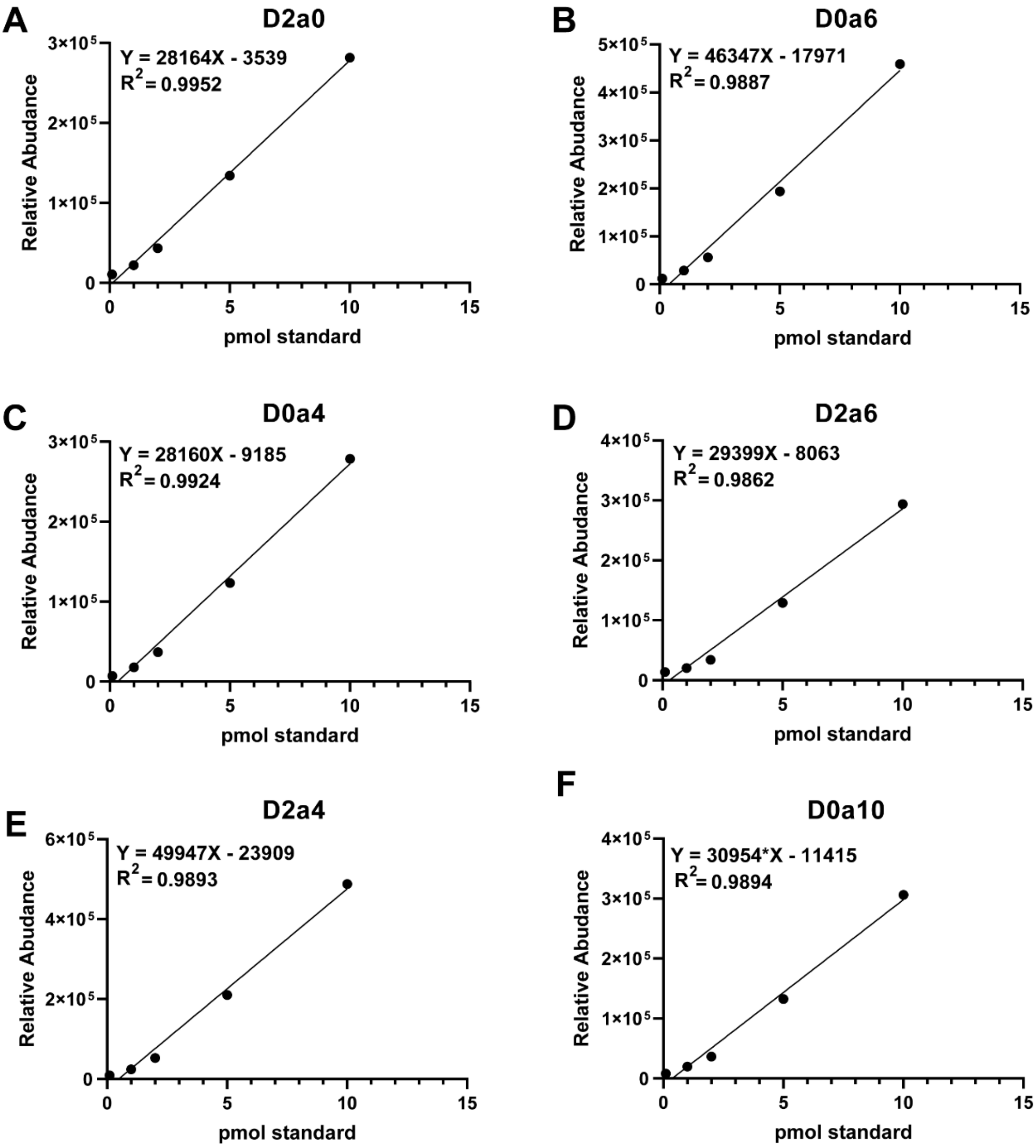

Supplemental Figure 3. Sensitivity and linear range for CS/DS disaccharides. Dynamic range of the HILIC-UPLC/MS analysis for [^{13}C] aniline-tagged (A) D2a0, (B) D0a6, (C) D0a4, (D) D2a6, (E) D2a4, and (F) D0a10 disaccharide standards. Disaccharides were mixed at equimolar concentrations, injected at various picomole amounts, and followed by HILIC-Q-TOF-MS analysis. The relative abundance at each disaccharide is plotted versus picomole amount injected.

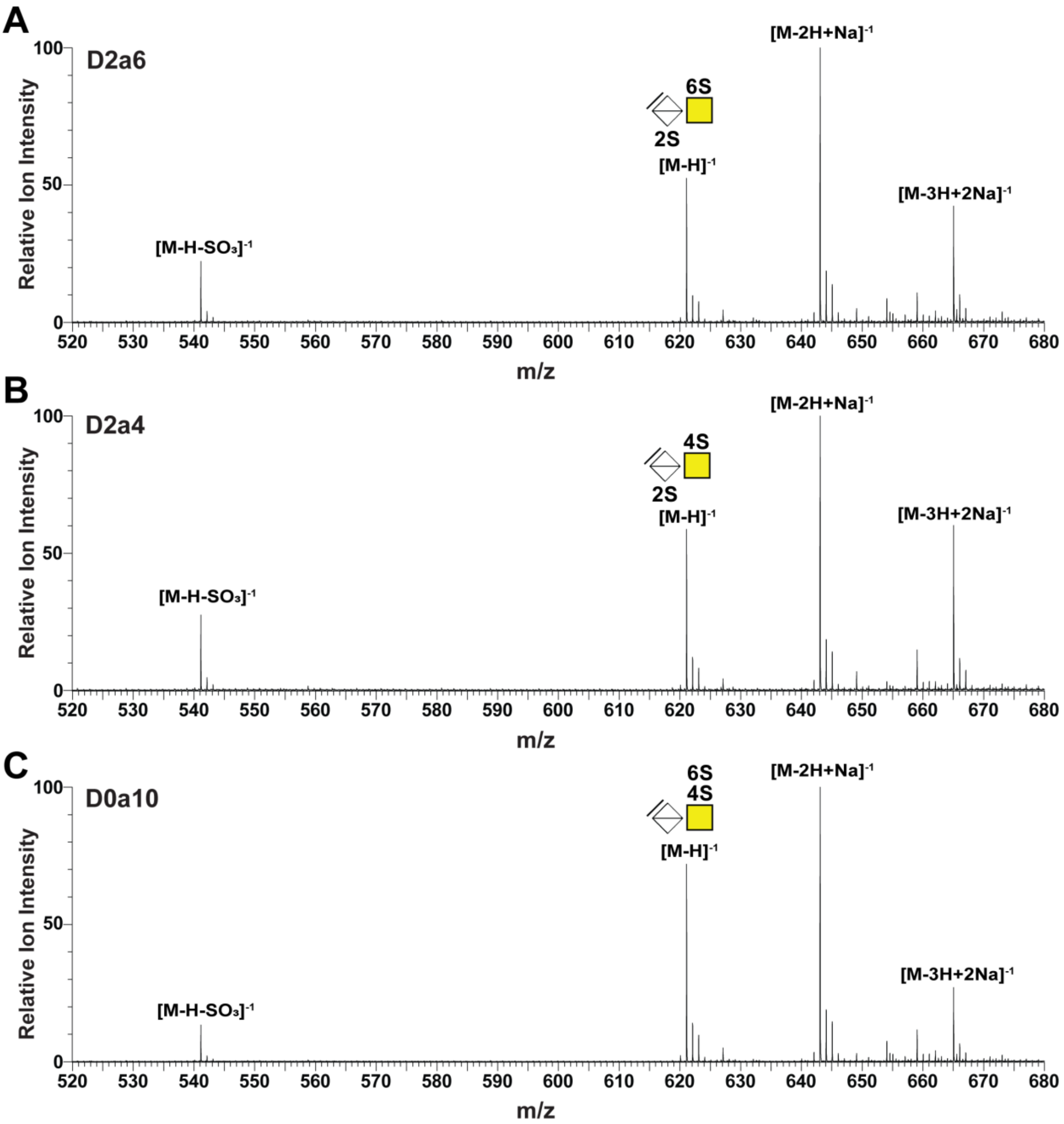

Supplemental Figure 4. TOF-MS mediated sulfate loss for di-sulfated CS/DS disaccharides. [^{13}C] aniline-tagged (A) D2a6, (B) D2a4, and (C) D0a10 disaccharide standards show some sulfate loss (541 m/z). Parent ions and sodium adducts are included for reference.

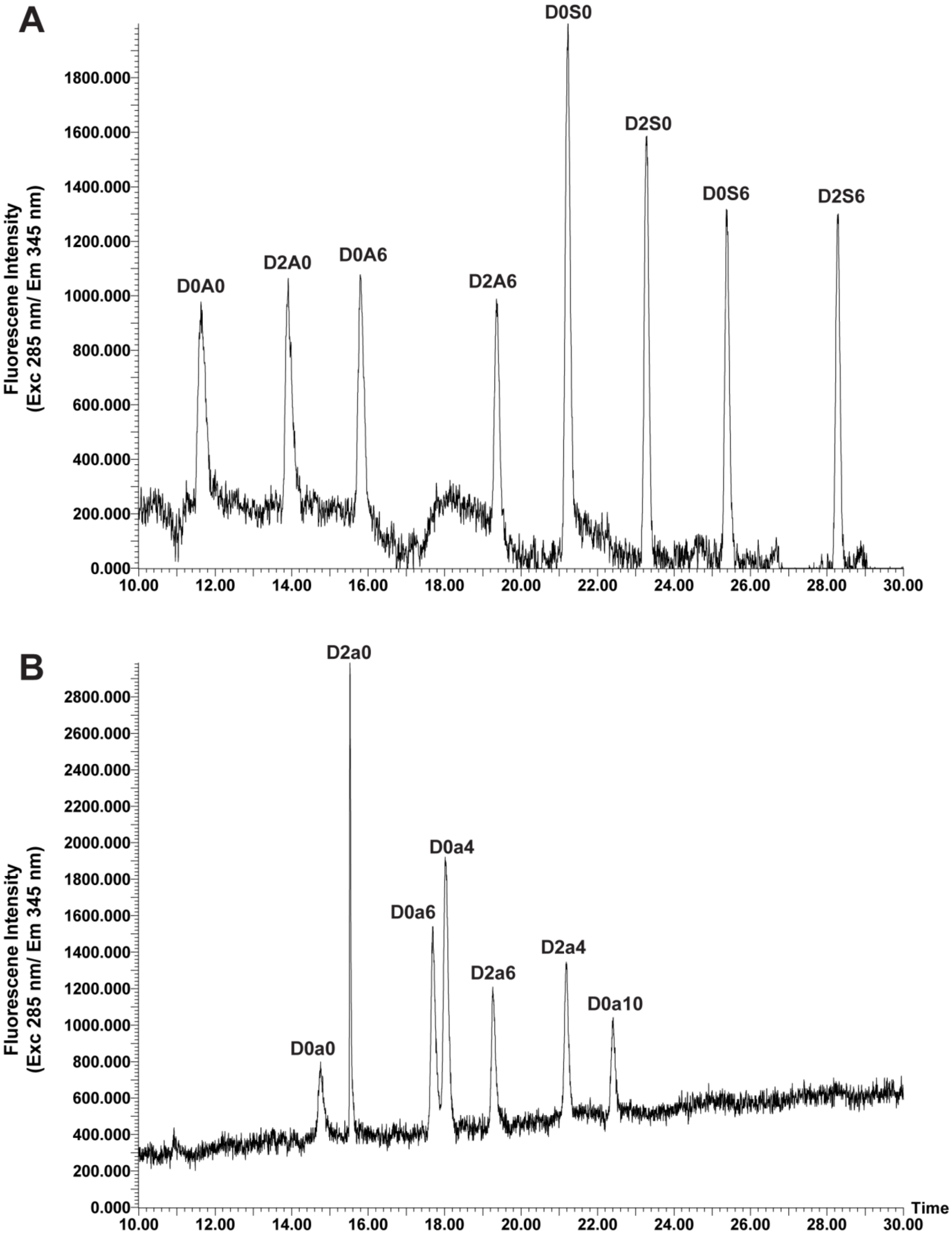

Supplemental Figure 5. Monitoring HS and CS/DS disaccharides using the UPLC fluorescence detector. [^{13}C] aniline-tagged (A) HS and (B) CS/DS disaccharide standards could be monitored via aniline fluorescence (285/345 nm) using an in-line fluorescence detector.

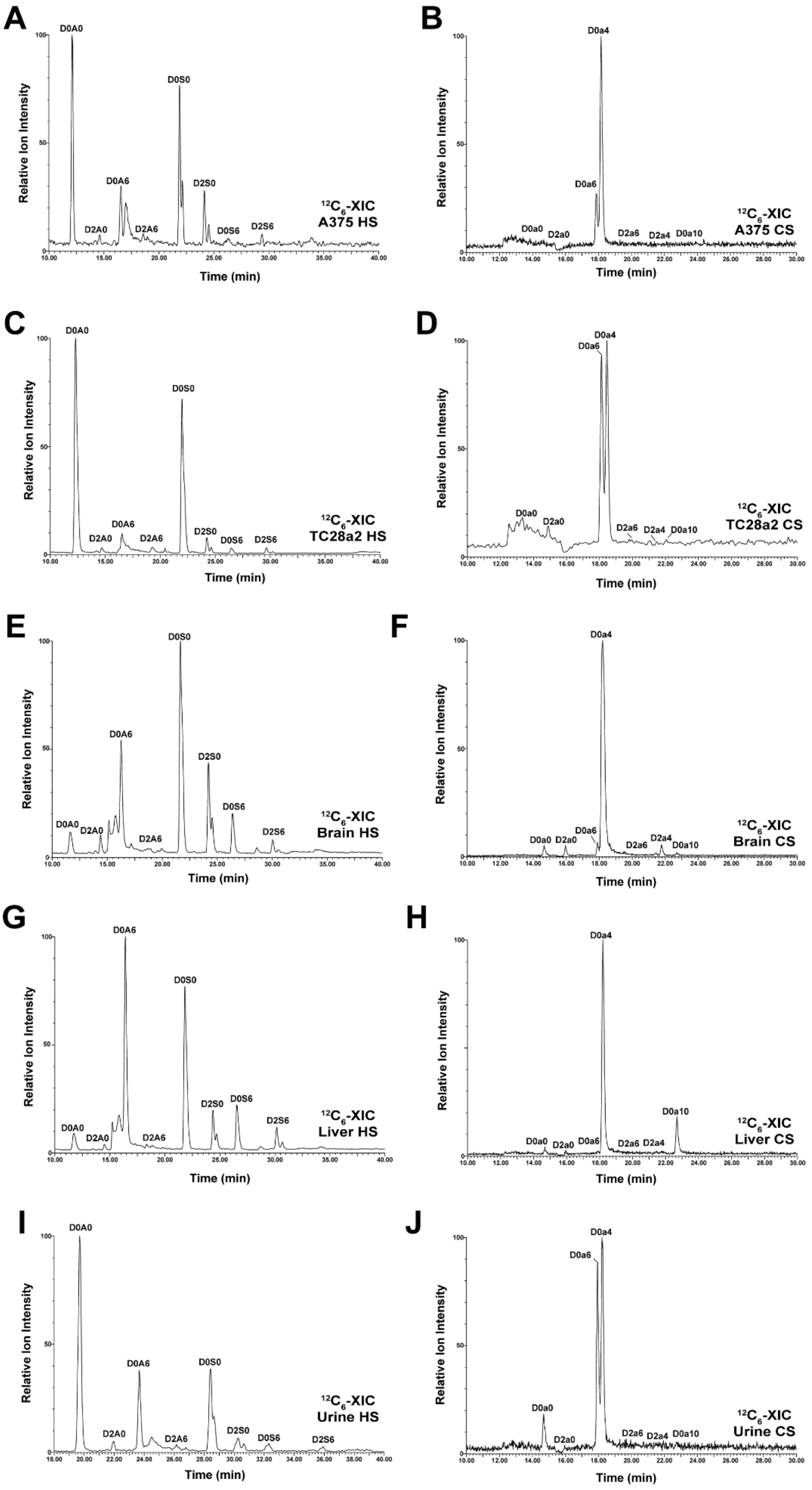

Supplemental Figure 6. XIC chromatographs for biological samples. XIC chromatographs for [$^{12}C_6$] aniline-tagged HS and CS/DS disaccharides for (A-B) A375, (C-D) TC28a2, (E-F) murine brain, (G-H) murine liver, and (I-J) human urine.

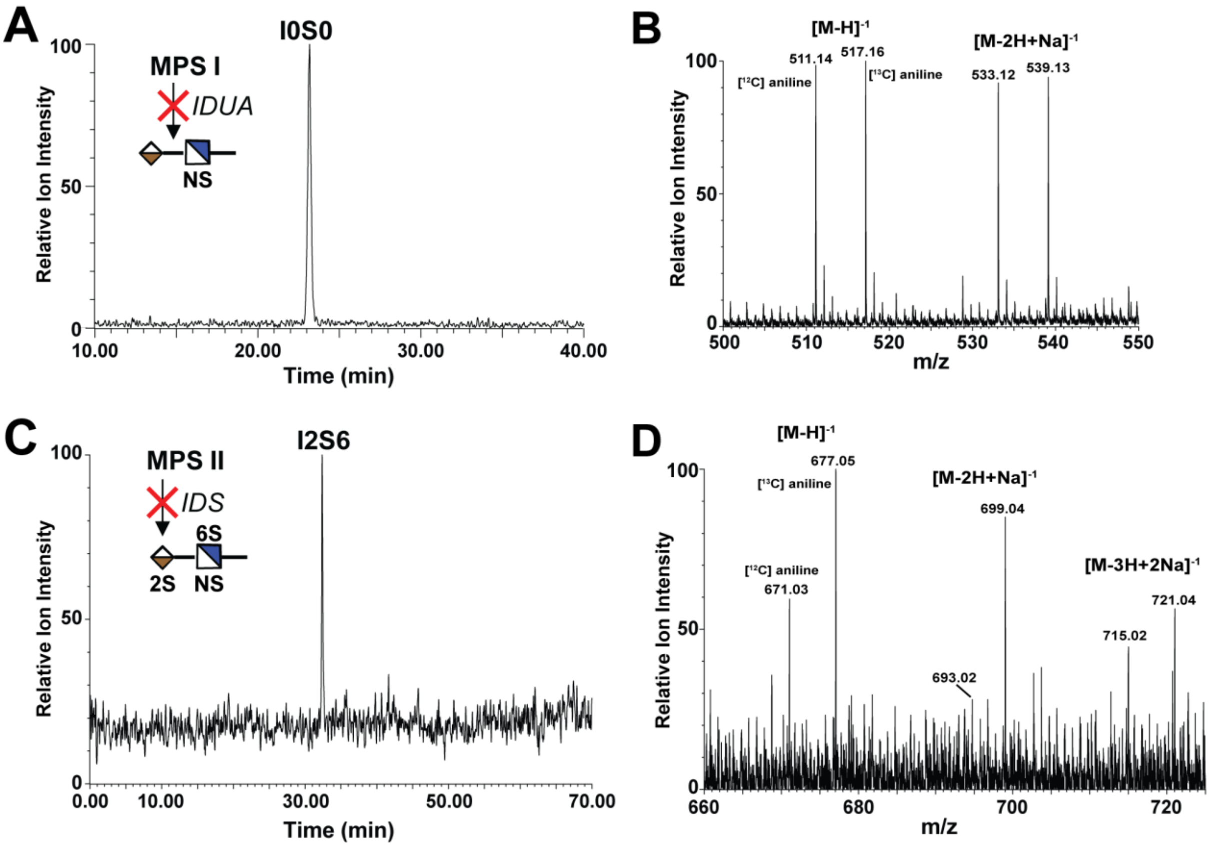

Supplemental Figure 7. I0S0 and I2S6 NRE synthetic standards. (A) XIC chromatogram for [^{13}C] aniline-tagged I0S0 NRE disaccharide standard. (B) XIC for the free molecular ions for [^{13}C] and [^{12}C] aniline-labeled MPS I NRE species, I0S0, $[M\text{-}H]^{-1}$ (m/z = 511, 517). Sodium adducts are also annotated. (C) XIC chromatogram for [^{13}C] aniline-tagged I2S6 NRE disaccharide standard. (D) XIC for the free molecular ions for [^{13}C] and [^{12}C] aniline-labeled MPS II NRE species, I2S6, $[M\text{-}H]^{-1}$ (m/z = 671, 677). Sodium adducts are also annotated.

Supplemental Table 1. Molecular masses (*m/z*) for [$^{12}C_6$] and [$^{13}C_6$] aniline GAG disaccharides

HS disaccharides		Non-derivatized	[$^{12}C_6$] Aniline-tagged				[$^{13}C_6$] Aniline-tagged			
Code	Structure	$[M-H]^{-1}$	$[M-H]^{-1}$	$[M-2H+Na]^{-1}$	$[M-3H+2Na]^{-1}$	$[M-4H+3Na]^{-1}$	$[M-H]^{-1}$	$[M-2H+Na]^{-1}$	$[M-3H+2Na]^{-1}$	$[M-4H+3Na]^{-1}$
D0A0	ΔUA-GlcNAc	378.10	455.17	–	–	–	461.19	–	–	–
D2A0	ΔUA2S-GlcNAc	458.10	535.12	557.11	–	–	541.14	563.13	–	–
D0A6	ΔUA-GlcNAc6S	458.10	535.12	557.11	–	–	541.14	563.13	–	–
D2A6	ΔUA2S-GlcNAc6S	538.00	615.08	637.06	659.04	–	621.10	643.08	665.06	–
D0S0	ΔUA-GlcNS	415.00	493.11	515.10	–	–	499.13	521.12	–	–
D2S0	ΔUA2S-GlcNS	493.99	573.06	595.05	617.04	–	579.08	601.07	623.06	–
D0S6	ΔUA-GlcNS6S	493.99	573.06	595.05	617.04	–	579.08	601.07	623.06	–
D2S6	ΔUA2S-GlcNS6S	575.97	653.03	675.01	696.99	718.98	659.05	681.03	703.01	724.99
CS disaccharides		Non-derivatized	[$^{12}C_6$] Aniline-tagged				[$^{13}C_6$] Aniline-tagged			
Code	Structure	$[M-H]^{-1}$	$[M-H]^{-1}$	$[M-2H+Na]^{-1}$	$[M-3H+2Na]^{-1}$	$[M-4H+3Na]^{-1}$	$[M-H]^{-1}$	$[M-2H+Na]^{-1}$	$[M-3H+2Na]^{-1}$	$[M-4H+3Na]^{-1}$
D0a0	ΔUA-GalNAc	378.10	455.17	–	–	–	461.19	–	–	–
D2a0	ΔUA2S-GalNAc	458.06	535.12	557.11	–	–	541. 14	563.13	–	–
D0a6	ΔUA-GalNAc6S	458.06	535.12	557.11	–	–	541. 14	563.13	–	–
D0a4	ΔUA-GalNAc4S	458.06	535.12	557.11	–	–	541. 14	563.13	–	–
D2a4	ΔUA2S-GalNAc4S	538.02	615.08	637.06	659.04	–	621.10	643.08	665.07	–
D2a6	ΔUA2S-GalNAc6S	538.02	615.08	637.06	659.04	–	621.10	643.08	665.07	–
D0a10	ΔUA-GalNAc4S6S	538.02	615.08	637.06	659.04	–	621.10	643.08	665.07	–

Supplemental Table 2: Molecular masses (*m/z*) for [$^{12}C_6$] and [$^{13}C_6$] aniline non-reducing end carbohydrates

NRE Species		Non-derivatized	[$^{12}C_6$] Aniline-tagged			[$^{13}C_6$] Aniline-tagged		
Code	Disease	$[M-H]^{-1}$	$[M-H]^{-1}$	$[M-2H+Na]^{-1}$	$[M-3H+2Na]^{-1}$	$[M-H]^{-1}$	$[M-2H+Na]^{-1}$	$[M-3H+2Na]^{-1}$
I0S0	MPS I	434.1	511.14	533.12	–	517.16	539.13	–
I2S6	MPS II	594.1	671.13	693.02	715.02	677.05	699.04	721.04
S0	MPS IIIA	258.1	335.08	n.d.	–	341.10	n.d.	–
H6	MPS IIID	258.1	335.08	n.d.	–	341.10	n.d.	–
G0S0	MPS VII	434.1	511.14	533.12	–	517.16	539.13	–

n.d. = not detected

General methods and materials. All reagents and solvents were purchased from commercial sources and were used without further purification. Anhydrous reactions were carried out under an argon atmosphere. Reactions were monitored using TLC on aluminium-backed plates coated with Silica Gel 60 F_{254} (E. Merck) and visualized by UV light (254 nM) where applicable, and by application of 5% H_2SO_4 in EtOH or with a solution of $(NH_4)_6Mo_7O_{24}.H_2O$ (Hanessian's stain, 25.0 gL^{-1}) in 10% H_2SO_4 in EtOH, as appropriate, and heating. Column chromatography was performed on silica gel G60 (Silicycle, 60-200 μm, 60 Å, normal phase), or on Bondapak C-18 (Waters, reverse phase). Size exclusion chromatography was carried out on a Sephadex™ LH-20 using MeOH/DCM (1/1, v/v) as elution system. Desalting was carried out on a Bio-Gel P-2 gel column using H_2O as elution. ^1H and ^{13}C NMR spectra were recorded on Varian 500 MHz, Bruker 600 MHz or Bruker 900 MHz spectrometer. Signals are reported in terms of chemical shift [δ in parts per million (ppm)] relative to tetramethylsilane (TMS) as the internal standard. NMR data is presented as follows: Chemical shift, multiplicity (s = singlet, br. s = broad singlet, d = doublet, t = triplet, dd = doublet of doublet, m = multiplet and/or multiple resonances), coupling constant in Hertz (Hz), integration. All NMR signals were assigned on the basis of ^1H NMR, ^{13}C NMR, COSY, TOCSY and HSQC experiments. Mass spectra were recorded on an ABISciex 5800 MALDi-TOF-TOF, Advion expression® compact mass spectrometers (CMS) or Shimadzu LCMS-IT-TOF mass spectrometer. The matrix used was 2,5-dihydroxy-benzoic acid (DHB) and ultramark 1621 as the internal standard. ‡Carbon assignments were extracted from HSQC experiment.

Experimental procedure and spectral data. Disaccharide standard **G0S0** (**1**) was prepared following the synthetic **Scheme S1** as depicted below.

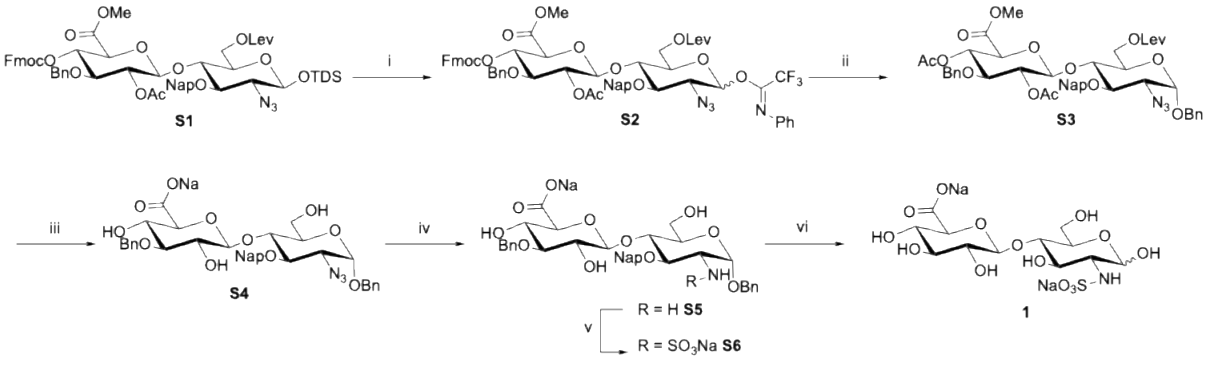

Scheme S1. Chemical synthesis of disaccharide standard G0S0 (**1**). *Reagents and conditions:* (i) a) HF.pyridine, THF, 0°C to rt, 16 h and b) trifluoro-*N*-phenylacetimidoyl chloride, NaH,

DCM, 0°C to rt, 1h; (ii) a) BnOH, TMSOTf, Toluene/Dioxane (1/3, v/v), MS 4 Å, rt, 1h b) Et_3N/DCM (1/4), rt, 1h c) pyridine, Ac_2O, DMAP (cat.), rt, 16h; (iii) 1.0 M aq. LiOH/THF (1/1, v/v), rt, 16h; (iv) 1.0 M PMe_3/THF, THF, NaOH, rt, 16 h; (v) SO_3.pyridine complex, MeOH, Et_3N, 0.1 M NaOH, 0°C to rt, 16 h; (vi) $Pd(OH)_2$/C, H_2 (atm.), *t*BuOH/H_2O, rt, 16h.

Dimethylthexylsilyl-*O*-(methyl-2-*O*-acetyl-3-*O*-benzyl-4-*O*-[9-fluorenylmethyloxy-carbonyl]-β-D-glucopyranosyluronate)-(1→4)-*O*-2-azido-2-deoxy-3-*O*-napthylmethyl-6-*O*-levulinoyl-α-D-glucopyranoside (**S1**):

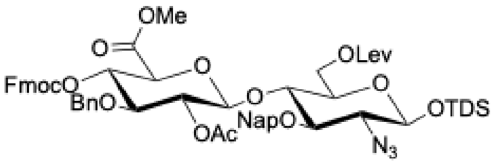

Disaccharide **S1** was prepared according to the literature procedure and spectral data is in close agreement with reported compound. ^{1}H NMR (500 MHz, $CDCl_3$): δ 8.00 – 7.14 (m, 20H, *Ar*H), 5.27 (d, *J* = 11.8 Hz, 1H, C*H*H-Nap), 5.14 (m, 2H, H-2^B and H-4^B), 4.96 (d, *J* = 11.8 Hz, 1H, CH*H*-Nap), 4.79 (d, *J* = 8.0 Hz, 1H, H-1^B), 4.71 (d, *J* = 11.7 Hz, 1H, C*H*H-Bn), 4.60 (d, *J* = 11.7 Hz, 1H, CH*H*-Bn), 4.50 (d, *J* = 7.7 Hz, 1H, H-1^A), 4.43 (dd, *J* = 10.5, 7.1 Hz, 1H, C*H*H-Fmoc), 4.38 – 4.29 (m, 2H, H-6A^A and CH*H*-Fmoc), 4.29 – 4.11 (m, 3H, H-6B^A, C*H*-Fmoc and H-5^B), 3.95 (t, *J* = 9.3 Hz, 1H, H-3^B), 3.85 (dd, *J* = 9.8, 8.5 Hz, 1H, H-4^A), 3.52 – 3.41 (m, 2H, H-5^A and H-3^A), 3.40 – 3.32 (m, 4H, COOCH_3 and H-2^A), 2.89 (m, 1H, C*H*H-Lev), 2.78 – 2.61 (m, 2H, CH_2-Lev), 2.55 (m, 1H, CH*H*-Lev), 2.23 and 2.03 (2 x s, 2 x 3H, 2 x COCH_3), 1.73 – 1.65 (m, 1H, C*H*(CH_3)$_2$), 1.01 – 0.81 (m, 12H, CH(CH_3)$_2$ and C(CH_3)$_2$) and 0.20 (d, *J* = 4.4 Hz, 6H, Si(CH_3)$_2$); ^{13}C NMR‡ (125 MHz, $CDCl_3$): δ 128.0 – 120.1 (*Ar*C), 101.0, 96.9, 80.7, 79.5, 78.5,75.3, 75.2, 75.2, 75.2, 74.5, 74.5, 74.5, 74.5, 72.7, 72.6, 72.5, 70.4, 70.3, 68.7, 62.5, 62.5, 52.7, 46.7, 38.0, 38.0, 37.9, 34.0, 30.0, 29.9, 27.9, 27.9, 20.9, 20.0, 19.2, 16.2, 16.5 and -2.5; MS (MALDI): calcd. for $C_{61}H_{71}NNaO_{16}Si$ [M+Na-N$_2$]$^+$ 1124.44, found 1124.26.

Benzyl-*O*-(methyl-2-*O*-acetyl-3-*O*-benzyl-4-*O*-[acetyl]-β-D-glucopyranosyluronate)-(1→4)-*O*-2-azido-2-deoxy-3-*O*-napthylmethyl-6-*O*-levulinoyl-α-D-glucopyranoside (**S3**):

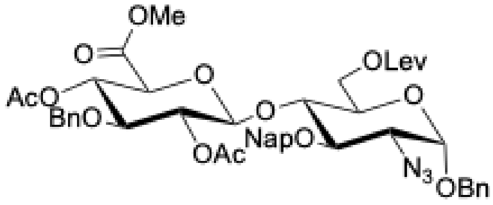

Compound **S1** (0.5 g, 0.44 mmol) was dissolved in THF (5.0 mL) and transferred to a plastic container, to which HF.pyridine (25 equiv.) was added dropwise at 0 °C at stirring. The progress of reaction was monitored by TLC (Hexanes/EtOAc, 1/1, v/v). After 16h, the reaction mixture was diluted with

EtOAc (10.0 mL) and added dropwise to the stirring saturated $NaHCO_3$ aqueous solution (50.0 mL) and stirring continued. Once the effervescence ceases, the solution was transferred to a separating flask and extracted with EtOAc (3 x 20.0 mL), combined organic layer were dried ($MgSO_4$), filtered, and concentrated under reduced pressure. The residue obtained was purified by flash chromatography using a gradient of Hexanes and EtOAc (from 9/1 to 1/9, v/v) to give desired lactol. Next, sodium hydride (NaH, 60% oil suspension, 2.0 equiv.) was added to a solution of lactol and trifluoro-*N*-phenylacetimidoyl chloride (2 equiv.) in DCM (2.0 mL) at 0 °C. After 2h, the reaction mixture was loaded directly onto a silica column (containing 10% K_2CO_3) and purified by flash chromatography using a gradient of Hexanes and EtOAc (from 4/1 to 1/4, v/v) to yield donor **S2** (0.4 g, 78.4%). Next, the disaccharide donor (**S2**, 0.15 g, 0.15 mmol) and benzyl alcohol (BnOH, 20 µL, 0.18 mmol) were combined and azeotroped with Toluene (3 x 3.0 mL), and then dissolved in anhyd. Toluene/Dioxane (1/3, v/v, 0.2 M based on the donor). Freshly activated powdered molecular sieves (4 Å, 1g for 5 mL) were added under argon atmosphere, and the mixture was stirred for 30 min at room temperature. TMSOTf (1.5 equiv) was added and the resulting reaction mixture was stirred for 1 h and then quenched by the addition of pyridine (5.0 µL). TLC analysis and MALDI (MS) confirms complete conversion to desired benzyl glycoside. The reaction mixture was filtered and concentrated under vaccum to afford a solid residue, which was taken in triethylamine/DCM (1/4, v/v, 5 mL) and stirred at rt. After 1h, reaction mixture was concentrated and residue obtained was taken in pyridine (5.0 mL), acetic anhydride (5.0 mL) and DMAP (cat.) and stirred at rt for overnight. Next, the mixture was concentrated under reduced pressure and the residue was purified by silica gel column chromatography using a gradient of Hexanes and EtOAc (from 9/1 to 1/9, v/v) to afford **S3** (0.1 g, 84%). ^{1}H NMR (600 MHz, $CDCl_3$): δ 8.05 – 7.13 (m, 17H, Ar*H*), 5.45 (d, *J* = 11.7 Hz, 1H, C*H*H-Nap), 5.26 (t, *J* = 9.5 Hz, 1H, H-2^B), 5.20 – 5.14 (m, 1H, H-4^B), 4.98 (d, *J* = 3.7 Hz, 1H, H-1^A), 4.93 – 4.87 (m, 1H, CH*H*-Nap), 4.79 (d, *J* = 8.0 Hz, 1H, H-1^B), 4.71 (d, *J* = 12.3 Hz, 1H, C*H*H-Bn), 4.67 – 4.61 (m, 3H, CH*H*-Bn and CH_2-Bn), 4.36 (dd, *J* = 12.3, 3.2 Hz, 1H, H-6A^A), 4.19 (d, *J* = 9.8 Hz, 1H, H-5^B), 4.16 – 4.10 (m, 1H, H-6B^A), 4.04 (m, 1H, H-3^B), 3.95 (m, 2H, H-4^A and H-5^A), 3.80 (m, 1H, H-3^A), 3.43 – 3.38 (m, 1H, H-2^A), 3.33 (s, 3H, COOCH_3), 3.02 – 2.91 (m, 1H, C*H*H-Lev), 2.77 – 2.63 (m, 2H, CH_2-Lev), 2.55 – 2.46 (m, 1H, CH*H*-Lev), 2.23, 2.03 and 1.95 (3 x s, 3 x 3H, 3 x COCH_3); ^{13}C NMR (151 MHz, $CDCl_3$): δ 206.7, 172.6, 169.5, 169.1, 167.6, 137.9, 136.6, 136.1, 133.4, 132.9, 128.6, 128.5, 128.4, 128.1, 128.1, 128.1, 127.9, 127.8, 127.7, 127.6, 126.4, 126.1, 125.8, 125.6, 100.8, 97.0, 79.6, 78.5, 78.1, 77.3, 77.1, 76.9, 75.6, 74.1, 72.8, 72.6, 71.2, 70.2, 68.9, 63.0, 62.0, 52.5,

38.0, 29.8, 27.9, 20.8 and 20.6; MS (MALDI): calcd. for $C_{60}H_{59}NNaO_{16}$ $[M+Na-N_2]^+$ 1072.37, found 1072.44.

Benzyl-*O*-(3-*O*-benzyl-β-D-glucopyranosyluronate)-(1→4)-*O*-2-azido-2-deoxy-3-*O*-napthylmethyl-α-D-glucopyranoside, sodium salt (**S4**):

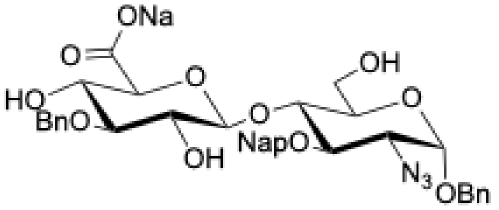

To the stirring solution of **S3** (0.1 g, 0.1 mmol) in THF (5.0 mL) was added aq. lithium hydroxide (aq. LiOH, 1.0 M, 1.0 mL). After 6h, the mixture was concentrated under reduced pressure and the residue obtained was purified by flash chromatography [silica gel column, 100% EA → 20% MeOH/EA) to afford **S4** (50 mg, 77 %) as white solid. ^{1}H NMR (600 MHz, MeOD): δ 8.02 – 7.20 (m, 17H, Ar*H*), 5.34 (d, *J* = 10.9 Hz, 1H, C*H*H-Nap), 5.01 (d, *J* = 3.5 Hz, 1H, H-1^A), 4.91 (merged with HDO, br.s, 2H, CH_2-Bn), 4.83 (d, *J* = 10.9 Hz, 1H, CH*H*-Nap), 4.75 (d, *J* = 11.9 Hz, 1H, C*H*H-Bn), 4.69 (d, *J* = 7.2 Hz, 1H, H-1^B), 4.59 (d, *J* = 11.9 Hz, 1H, CH*H*-Bn), 4.10 – 4.00 (m, 3H, H-6A^A, H-5^B and H-4^B), 3.90 – 3.76 (m, 4H, H-6B^A, H-3^B, H-4^A and H-5^A), 3.52 – 3.38 (m, 3H, H-2^B, H-3^A and H-2^A); ^{13}C NMR (151 MHz, MeOD): δ 171.4, 138.9, 137.2, 136.0, 133.4, 133.1, 128.1, 128.0, 127.8, 127.8, 127.8, 127.8, 127.7, 127.5, 127.5, 127.5, 127.2, 127.1, 126.9, 126.7, 126.6, 125.4, 125.4, 125.4, 103.2, 103.2, 96.9, 84.2, 78.2, 77.0, 75.5, 75.5, 74.7, 74.7, 74.1, 71.9, 71.9, 71.8, 69.1, 62.9, 59.8, 48.5, 48.1, 48.0, 47.9, 47.8, 47.8, 47.7, 47.7, 47.6, 47.6, 47.5, 47.4, 47.2 and 47.2; MS (CMS, ESI): calcd. for $C_{37}H_{38}N_3O_{11}$ $[M-H-Na]^-$ 700.3, found 700.5.

Benzyl-*O*-(3-*O*-benzyl-β-D-glucopyranosyluronate)-(1→4)-*O*-2-deoxy-2-sulfamino-3-*O*-napthylmethyl-α-D-glucopyranoside, sodium salt (**S6**):

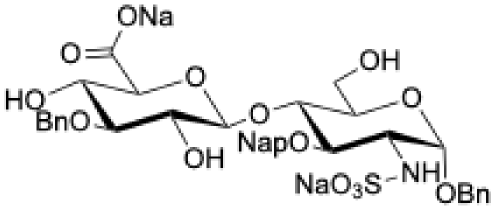

To the solution of **S4** (50 mg, 0.07 mmol) in THF (5.0 mL), trimethyl phosphine (PMe_3, 10 equiv., 1.0 M sol in THF) and aq. NaOH solution (0.1 M, 20 equiv.,) were added, and the reaction mixture was stirred for 2h. The progress of reaction was monitored by TLC (EtOAc/MeOH/H_2O 7/2/1, v/v/v). Upon completion, the pH was adjusted to 8.0–8.5 by careful addition of glacial AcOH, and the mixture was concentrated in vacuo. The residue obtained was purified by reverse phase chromatography (RP-18 silica gel column, H_2O/MeOH, 9/1→1/9, v/v). The appropriate

fractions were concentrated in vacuo, and the residue was passed through a column Dowex® 50 x 8 Na^+ resin (0.6 x 5 cm) using MeOH/H_2O (1/1, v/v) as eluent. The appropriate fractions were combined and lyophilized to afford **S5** (40 mg, 83.3 %) as sodium salt. The amino sugar (**S5**, 40 mg, 0.06 mmol) was dissolved in a mixture of MeOH (2.0 mL), Et_3N (0.3 mL) and aq. NaOH solution (0.1 M, 1.0 mL) at 0 °C was added SO_3.Py complex (5.0 equiv.). The progress of reaction was monitored by TLC (EtOAc/pyridine/H_2O/AcOH 8/5/3/1, v/v/v/v). Additional portions of SO_3.Py complex were added at 0 °C after 1h, 2h, 4h and 8h. After stirring for an additional 16h, the reaction mixture was concentrated in vacuo and the residue was passed through a column of Dowex® 50 x 8 Na^+ resin (0.6 x 5 cm) using H_2O/MeOH (9/1, v/v) as eluent to afford desired product. The appropriate fractions were concentrated in vacuo and further was by reverse phase chromatography (RP-18 silica gel column, H_2O/MeOH, 9.5/0.5→1/1, v/v). Appropriate fractions were lyophilized to afford desired product **S6** as sodium salt (25 mg, 58.1 %). ^{1}H NMR (600 MHz, D_2O): δ 7.92 – 7.76 (m, 4H, Ar*H*), 7.57 (dd, *J* = 8.4, 1.7 Hz, 1H, Ar*H*), 7.44 (tt, *J* = 6.9, 5.2 Hz, 2H, Ar*H*), 7.41 – 7.24 (m, 10H, Ar*H*), 5.22 (d, *J* = 3.5 Hz, 1H, H-1^A), 4.97 – 4.85 (m, 2H, CH_2-Nap), 4.68 – 4.61 (m, 3H, CH_2-Bn and C*H*H-Bn), 4.52 (d, *J* = 11.3 Hz, 1H, CH*H*-Bn), 4.47 (d, *J* = 7.9 Hz, 1H, H-1^B), 3.89 – 3.67 (m, 5H, H-$6A^A$, H-$6B^A$, H-5^A, H-3^A and H-4^A), 3.56 – 3.45 (m, 2H, H-5^B and H-4^B), 3.39 (dd, *J* = 10.4, 3.6 Hz, 1H, H-2^A), 3.35 (t, *J* = 8.8 Hz, 1H, H-3^B), 3.21 (dd, *J* = 9.4, 7.8 Hz, 1H, H-2^B); ^{13}C NMR (151 MHz, D_2O): δ 175.0, 137.5, 137.1, 135.3, 132.9, 132.8, 128.7, 128.6, 128.6, 128.5, 128.3, 128.3, 128.1, 128.1, 127.8, 127.6, 127.3, 126.3, 102.0, 97.2, 83.5, 77.5, 76.2, 75.9, 74.3, 73.1, 71.3, 71.1, 70.4, 60.1 and 57.4; HRMS (ESI): calcd. for $C_{37}H_{39}NO_{14}S$ $[M-2Na]^{2-}$ 376.6045, found 376.4783.

β-D-glucopyranosyluronate-(1→4)-*O*-2-deoxy-2-*N*-sulfamino-α/β-D-glucopyranose, sodium salt (G0S0, **1**):

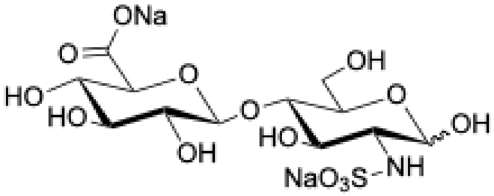

To a stirring solution of disaccharide **S6** (10 mg, 0.01 mmol) in *t*BuOH/H_2O (1/1, v/v, 5.0 mL) at room temperature was added Pd(OH)2/C (10%, 1.5 times the weight of the starting material). The reaction mixture was then placed under an atmosphere of H_2 gas (ballon at atmospheric pressure) and repeated cycle of vaccuming and H_2 gas flushing (3-4 times) were performed. After 16h, the reaction mixture was filtered through PTFE syringe filter, concentrated under vaccum and lyophilized. The residue obtained was disolved in minimum volume of H_2O (~0.2 mL) and

passed through a column of Dowex® 50 x 8 Na+ resin (0.6 x 5 cm) using H_2O as eluant. Appropriate fractions were concentrated under vaccum to minimum volume (~0.2 mL) and loaded on to a Bio-Gel P-2 size exclusion chromatography (SEC) column (1.5 x 80 cm). The compound was eluted using H_2O as eluant and desired fractions (checked with spotTLC, stained with Hanessian's stain) were combined and lyophilized to afford **1** (G0S0, 5 mg, 83.3 %) as white fluffy solid (reducing end GlcN α/β mixture, only α-anomer is reported). ^{1}H NMR (600 MHz, D_2O): δ 5.37 (d, J = 3.5 Hz, 1H, H-1^{A}), 4.46 (dd, J = 8.0, 3.0 Hz, 1H, H-1^{B}), 3.90 – 3.84 (m, 1H, H-5^{B}), 3.80 – 3.76 (m, 2H, H-6A^{A} and H-6B^{A}), 3.72 – 3.58 (m, 3H, H-4^{B}, H-5^{A} and H-3^{A}), 3.50 – 3.38 (m, 2H, H-4^{A} and H-3^{B}), 3.29 (t, J = 8.4 Hz, 1H, H-2^{B}), 3.16 (dd, J = 10.1, 3.6 Hz, 1H, H-2^{A}); ^{13}C NMR‡ (151 MHz, D_2O): δ 102.0, 90.8, 78.8, 75.8, 75.3, 72.9, 71.7, 70.0, 69.4, 60.0 and 57.7.

Disaccharide standard **I0S0** (**2**) was prepared following the synthetic **Scheme S2** as depicted below.

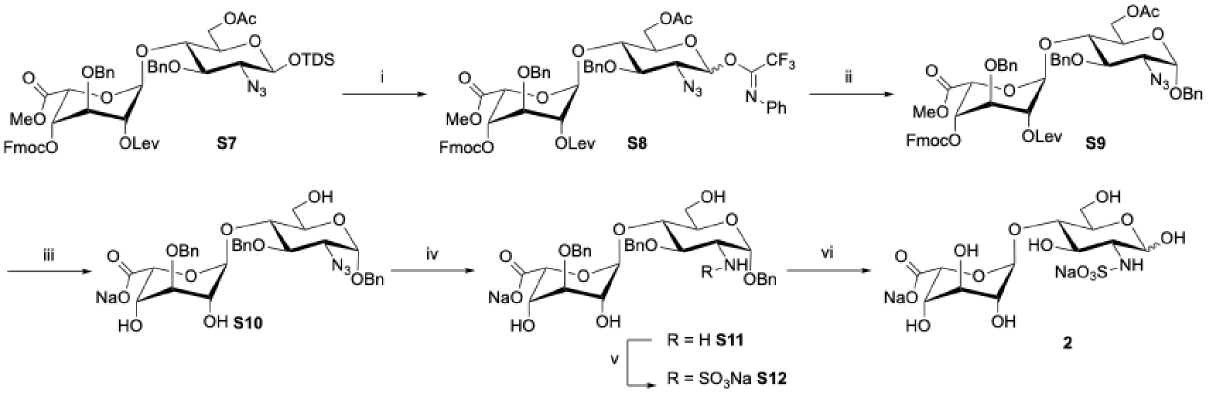

Scheme S2. Chemical synthesis of disaccharide standard I0S0 (**2**). *Reagents and conditions:* (i) a) HF.pyridine, THF, 0°C to rt, 16 h and b) trifluoro-*N*-phenylacetimidoyl chloride, NaH, DCM, 0°C to rt, 1h; (ii) BnOH, TMSOTf, Toluene/Dioxane (1/3, v/v), MS 4 Å, rt, 1h; (iii) 1.0 M aq. LiOH/THF (1/1, v/v), rt, 16h; (iv) 1.0 M PMe_3/THF, THF, NaOH, rt, 16 h; (v) SO_3.pyridine complex, MeOH, Et_3N, 0.1 M NaOH, 0°C to rt, 16 h; (vi) $Pd(OH)_2$/C, H_2 (atm.), *t*BuOH/H_2O, rt, 16h.

Dimethylthexylsilyl-*O*-(methyl-2-*O*-levulinoyl-3-*O*-benzyl-4-*O*-[9-fluorenylmethyloxy carbonyl]-α-L-idopyranosyluronate)-(1→4)-*O*-2-azido-2-deoxy-3-*O*-benzyl-6-*O*-acetyl-α-D-glucopyranoside (**S7**):

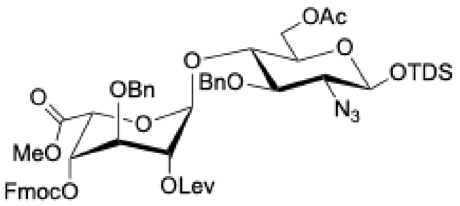

Disaccharide **S7** was prepared according to the literature procedure and spectral data is in close agreement with reported compound. ^{1}H NMR (300 MHz, $CDCl_3$): δ 7.76 – 7.21 (m, 18H, *Ar*H), 5.15 (s, 1H, H-1^B), 5.06 – 5.02 (m, 2H, H-5^B and H-4^B), 4.92 (t, *J* = 2.7 Hz, 1H, H-2^B), 4.78 – 4.63 (m, 4H, 2 x C*H*2-Bn), 4.57 – 4.47 (m, 3H, C*H*H-Fmoc, H-1^A and H-6A^A), 4.39 (dd, *J* = 10.5, 7.2 Hz, 1H, H-6B^A), 4.23 – 4.13 (m, 2H, C*H*-Fmoc and CH*H*-Fmoc), 3.91 – 3.88 (m, 2H, H-4^A and H-3^B), 3.53 – 3.50 (m, 1H, H-5^A), 3.45 (s, 3H, COOCH_3), 3.41 – 3.20 (m, 2H, H-2^A and H-3^A), 2.75 – 2.39 (m, 4H, 2 x CH_2-Lev), 2.11 and 2.04 (2 x s, 2 x 3H, 2 x COCH_3), 1.73 – 1.62 (m, 1H, C*H*(CH_3)$_2$), 0.96 – 0.90 (m, 12H, CH(CH_3)$_2$ and C(CH_3)$_2$) and 0.21 (d, *J* = 3.0 Hz, 6H, Si(CH_3)$_2$); ^{13}C NMR (75 MHz, $CDCl_3$): δ 206.1, 171.6, 170.5, 168.4, 154.3, 143.2, 143.1, 141.3, 141.3, 138.0, 137.3, 128.5, 128.2, 128.1, 128.0, 128.0, 127.5, 127.4, 127.2, 127.2, 125.1, 125.0, 120.1, 97.5, 97.0, 80.9, 74.8, 74.7, 73.2, 72.9, 72.9,

71.3, 70.1, 68.9, 67.3, 66.8, 62.3, 52.2, 46.6, 37.6, 34.0, 29.5, 29.3, 28.0, 24.8, 20.9, 20.0, 19.9, 18.5, 18.4, -2.1 and -3.2; MS (MALDI): calcd. for $C_{57}H_{69}N_3NaO_{16}Si$ $[M+Na]^+$ 1103.44, found 1103.00.

Benzyl-*O*-(methyl-2-*O*-levulinoyl-3-*O*-benzyl-4-*O*-[9-fluorenylmethyloxycarbonyl]-α-L-idopyranosyluronate)-(1→4)-*O*-2-azido-2-deoxy-3-*O*-benzyl-6-*O*-acetyl-α-D-glucopyranoside (**S9**):

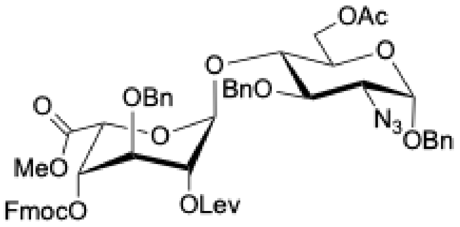

TDS-disaccharide **S7** (1.0 g, 0.9 mmol) was dissolved in THF (5.0 mL) and transferred to a plastic container, to which HF.pyridine (25 equiv.) was added dropwise at 0 °C. The progress of reaction was monitored by TLC (Hexanes/EtOAc, 1/1, v/v). After 16h, the reaction mixture was diluted with ethyl acetate (10.0 mL) and added dropwise to the stirring saturated $NaHCO_3$ aqueous solution (50.0 mL). Once the effervescence ceases, the solution was transferred to a separating flask and extracted with ethyl acetate (3 x 20.0 mL), combined organic layer were dried ($MgSO_4$), filtered, and concentrated under reduced pressure. The residue obtained was purified by silica gel column chromatography using a gradient of Hexanes and EtOAc (from 9/1 to 1/9, v/v) to give desired lactol. To the solution of lactol and trifluoro-*N*-phenylacetimidoyl chloride (2 equiv.) in DCM (2.0 mL) was added sodium hydride (NaH, 60% oil suspension, 2.0 equiv.) at 0 °C. After 2h, the reaction mixture was loaded directly onto a silica column (containing 10% K_2CO_3) and purified by flash chromatography using a gradient of Hexanes and EtOAc (from 4/1 to 1/4, v/v) to yield donor **S8** (0.8 g, 80%). Next, the disaccharide donor (**S8**, 0.6 g, 0.52 mmol) and benzyl alcohol (BnOH, 66 μL, 0.62 mmol) were combined and azeotroped with Toluene (3 x 3.0 mL), and then dissolved in anhyd. Toluene/Dioxane (1/3, v/v, 0.2 M based on the donor). Freshly activated powdered molecular sieves (4 Å, 1g for 5 mL) were added under argon atmosphere, and the mixture was stirred for 30 min at room temperature. TMSOTf (1.5 equiv) was added and the resulting reaction mixture was stirred for 1 h and then quenched by the addition of pyridine (5.0 μL). TLC analysis and MALDI (MS) confirms complete conversion to desired benzyl glycoside. The mixture was filtered, the filtrate was concentrated under reduced pressure and the residue was purified by silica gel column chromatography using a gradient of Hexanes and EtOAc (from 9/1 to 1/9, v/v) to afford **S9** (0.45 g, 84.9%). ^1H NMR (600 MHz, $CDCl_3$): δ 7.79 – 7.25 (m, 23H, *Ar*H),

5.20 (br.s, 1H, H-1^{B}), 5.06 – 5.03 (m, 2H, H-1^{A} and H-5^{B}), 4.97 (m, 2H, H-4^{B} and H-2^{B}), 4.82 – 4.68 (m, 5H, 2 x C*H*2-Bn and C*H*H-Bn), 4.64 (d, *J* = 11.9 Hz, 1H, CH*H*-Bn), 4.53 (dd, *J* = 10.6, 7.2 Hz, 1H, C*H*H-Fmoc), 4.49 – 4.40 (m, 2H, CH*H*-Fmoc and H-6A^{A}), 4.30 (dd, *J* = 12.5, 3.1 Hz, 1H, H-6B^{A}), 4.25 (t, *J* = 7.2 Hz, 1H, C*H*-Fmoc), 4.05 – 3.88 (m, 4H, H-4^{A}, H-3^{B}, H-5^{A} and H-3^{A}), 3.48 – 3.44 (m, 3H, COOC*H*3 and H-2^{A}), 2.77 – 2.45 (m, 4H, 2 x CH2-Lev), 2.19 and 2.09 (2 x s, 2 x 3H, 2 x COC*H*3); ^{13}C NMR‡ (151 MHz, $CDCl_3$): δ 134.5 – 121.8 (Ar*C*), 97.4, 96.7, 78.4, 74.9, 74.8, 73.2, 73.1, 71.4, 70.2, 70.0, 69.4, 67.3, 63.4, 62.3, 51.9, 46.7, 37.8, 29.9, 27.8 and 20.8; MS (MALDI): calcd. for $C_{56}H_{57}NNaO_{16}$ $[M+Na-N_2]^+$ 1022.36, found 1022.69.

Benzyl-*O*-(3-*O*-benzyl-α-L-idopyranosyluronate)-(1→4)-*O*-2-azido-2-deoxy-3-*O*-benzyl-α-D-glucopyranoside, sodium salt (**S10**):

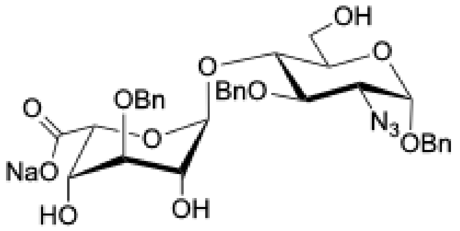

To the stirring solution of **S9** (0.1 g, 0.1 mmol) in THF (5.0 mL) was added aq. lithium hydroxide (aq. LiOH, 1.0 M, 1.0 mL). After 16h, the mixture was concentrated under reduced pressure and the residue obtained was purified by flash chromatography [silica gel column, 100% EA → 20% MeOH/EA) to afford **S10** (58 mg, 89.2%) as white solid. ^{1}H NMR (600 MHz, MeOD): δ 7.53 – 7.19 (m, 15H, *Ar*H), 5.09 (d, *J* = 10.6 Hz, 1H, C*H*H-Bn), 4.99 (d, *J* = 3.6 Hz, 1H, H-1^{B}), 4.96 – 4.93 (m, 2H, H-1^{A} and CH*H*-Bn), 4.83 – 4.79 (m, 2H, C*H*H-Bn and CH*H*-Bn), 4.70 – 4.65 (m, 1H, C*H*H-Bn), 4.59 (d, *J* = 11.9 Hz, 1H, CH*H*-Bn), 4.28* (d, *J* = 5.5 Hz, 1H, H-5^{B}), 4.17* (t, *J* = 9.3 Hz, 1H, H-3^{B}), 4.01 – 3.80 (m, 6H, H-4^{B}, H-6A^{A}, H-6B^{A}, H-5^{A}, H-2^{B} and H-4^{A}), 3.55 – 3.40 (m, 2H, H-3^{A} and H-2^{A}); ^{13}C NMR‡ (151 MHz, MeOD): δ 127.8 – 127.1 (multiple Ar*C*), 99.5, 96.6, 81.7, 77.8, 75.6, 74.1, 73.9, 73.0, 72.0, 71.7, 69.3, 62.9 and 60.8; MS (CMS, ESI): calcd. for $C_{33}H_{36}N_3O_{11}$ $[M-H-Na]^-$ 650.2, found 650.6.

Benzyl-*O*-(3-*O*-benzyl-α-L-idopyranosyluronate)-(1→4)-*O*-2-deoxy-2-sulfamino-3-*O*-benzyl-α-D-glucopyranoside, sodium salt (**S12**):

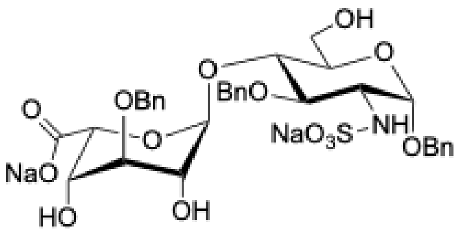

To the solution of azido sugar (**S10**, 55 mg, 0.08 mmol) in THF (5.0 mL), trimethyl phosphine (PMe_3, 10 equiv., 1.0 M sol in THF) and aq. NaOH solution (0.1 M, 20 equiv.) were added, and the reaction mixture was stirred for 2h. The progress of reaction was monitored by TLC

(EtOAc/MeOH/H_2O 7/2/1, v/v/v). Upon completion, the pH was adjusted to 8.0–8.5 by careful addition of glacial AcOH, and the mixture was concentrated in vacuo. The residue obtained was purified by reverse phase chromatography (RP-18 silica gel column, H_2O/MeOH, 9/1→1/9, v/v). The appropriate fractions were concentrated in vacuo, and the residue was passed through a column Dowex® 50 x 8 Na^+ resin (0.6 x 5 cm) using MeOH/H_2O (1/1, v/v) as eluent. The appropriate fractions were combined and lyophilized to afford **S11** (45 mg, 84.9%) as sodium salt. The amino sugar (**S11**, 45 mg, 0.07 mmol) was dissolved in a mixture of MeOH (2.0 mL), Et_3N (0.3 mL) and aq. NaOH solution (0.1 M, 1.0 mL) at 0 °C was added SO_3.Py complex (5.0 equiv.). The progress of reaction was monitored by TLC (EtOAc/pyridine/H_2O/AcOH 8/5/3/1, v/v/v/v). Two additional portions of SO_3.Py complex were added at 0 °C after 1h, 2h, 4h and 8h. After stirring for an additional 8h, the reaction mixture was concentrated in vacuo and the residue was passed through a column of Dowex® 50 x 8 Na^+ resin (0.6 x 5 cm) using H_2O/MeOH (9/1, v/v) as eluent to afford desired product. The appropriate fractions were concentrated in vacuo and further was by reverse phase chromatography (RP-18 silica gel column, H_2O/MeOH, 9.5/0.5→1/1, v/v). Appropriate fractions were lyophilized to afford **S12** as sodium salt (35 mg, 66.0%). ^1H NMR (600 MHz, D_2O): δ 7.42 – 6.91 (m, 15H, *Ar*H), 5.17 (d, *J* = 3.5 Hz, 1H, H-1^A), 4.85 (d, *J* = 2.8 Hz, 1H, H-1^B), 4.59 – 4.35 (m, 7H, 3 x CH_2-Bn and H-5^B), 3.96 (t, *J* = 3.5 Hz, 1H, H-4^B), 3.74 (t, *J* = 9.5 Hz, 1H, H-4^A), 3.76 – 3.53 (m, 5H, H-$6A^A$, H-$6B^A$, H-5^A, H-2^B and H-3^B), 3.44 (t, *J* = 9.8 Hz, 1H, H-3^A), 3.28 (dd, *J* = 10.5, 3.5 Hz, 1H, H-2^A); ^{13}C NMR (151 MHz, D_2O): δ 175.0, 137.7, 137.4, 137.0, 129.0, 128.6, 128.5, 128.4, 128.4, 128.2, 128.1, 128.0, 127.9, 100.6, 97.4, 77.1, 77.0, 75.4, 74.9, 72.2, 71.6, 70.2, 69.7, 68.6, 67.3, 59.9 and 57.4; HRMS (ESI): calcd. for $C_{33}H_{37}NO_{14}S$ $[M-2Na]^{1-}$ 703.1937, found 703.9665.

α-L-idopyranosyluronate-(1→4)-*O*-2-deoxy-2-*N*-sulfamino-α/β-D-glucopyranose, sodium salt (I0S0, **2**):

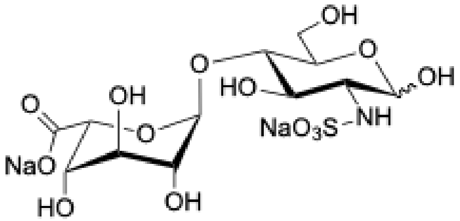

To a stirring solution of disaccharide **S12** (10 mg, 0.01 mmol) in *t*BuOH/H_2O (1/1, v/v, 5.0 mL) at room temperature was added $Pd(OH)_2$/C (10%, 1.5 times the weight of the starting material). The reaction mixture was then placed under an atmosphere of H_2 gas (ballon at atmospheric pressure) and repeated cycle of vaccuming and H_2 gas flushing (3-4 times) were performed. After 16h, the reaction mixture was filtered through PTFE syringe filter, concentrated under vaccum and lyophilized. The

residue obtained was disolved in minimum volume of H_2O (~0.2 mL) and passed through a column of Dowex® 50 x 8 Na+ resin (0.6 x 5 cm) using H_2O as eluant. Appropriate fractions were concentrated under vaccum to minimum volume (~0.2 mL) and loaded on to a Bio-Gel P-2 size exclusion chromatography (SEC) column (1.5 x 80 cm). The compound was eluted using H_2O as eluant and desired fractions (checked with spotTLC, stained with Hanessian's stain) were combined and lyophilized to afford **2** (I0S0, 5.5 mg, 87.3 %) as white fluffy solid (reducing end GlcN α/β mixture, only α-anomer is reported). ^{1}H NMR (600 MHz, D_2O): δ 5.37 (d, *J* = 3.5 Hz, 1H, H-1^A), 4.74 (d, *J* = 5.7 Hz, 1H, H-1^B), 4.48 (d, *J* = 4.5 Hz, 1H, H-5^B), 3.90 – 3.82 (m, 1H, H-5^A), 3.82 – 3.68 (m, 2H, H-6A^A and H-6B^A), 3.69 – 3.55 (m, 3H, H-4^A, H-3^A and H-3^B), 3.39 (dt, *J* = 8.0, 5.6 Hz, 1H, H-2^B), 3.17 (dd, *J* = 10.1, 3.6 Hz, 1H, H-2^A); ^{13}C NMR[‡] (151 MHz, D_2O): δ 101.0, 90.9, 77.8, 72.4, 71.5, 71.4, 71.0, 70.4, 69.4, 60.2 and 58.0.

Disaccharide standard **I2S6** (**3**) was prepared following the synthetic **Scheme S3** as depicted below.

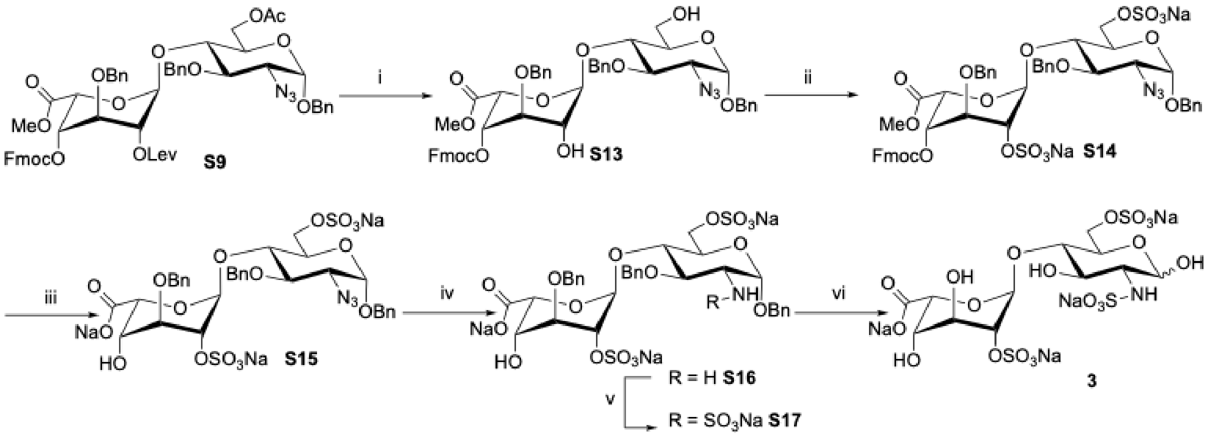

Scheme S3. Chemical synthesis of disaccharide standard I2S6 (**3**). *Reagents and conditions:* (i) a) Hydrazine acetate, Toluene/EtOH (1/2, v/v), rt, 2 h and b) Acetyl chloride/MeOH (1/9, v/v), 0°C to rt, 1h; (ii) SO_3.pyridine, DMF, rt, 16h (iii) a) 1.0 M aq. LiOH, aq. H_2O_2 (50%), rt, 24h and b) MeOH, adjust pH~14 with 4.0 M NaOH, rt, 16h; (iv) 1.0 M PMe_3/THF, THF, NaOH, rt, 16 h; (v) SO_3.pyridine complex, MeOH, Et_3N, 0.1 M NaOH, 0°C to rt, 16 h; (vi) $Pd(OH)_2$/C, H_2 (atm.), *t*BuOH/H_2O, rt, 16h.

Benzyl-*O*-(2-*O*-sulfonate-3-*O*-benzyl-α-L-idopyranosyluronate)-(1→4)-*O*-2-azido-2-deoxy-3-*O*-benzyl-α-D-glucopyranoside, sodium salt (**S14**):

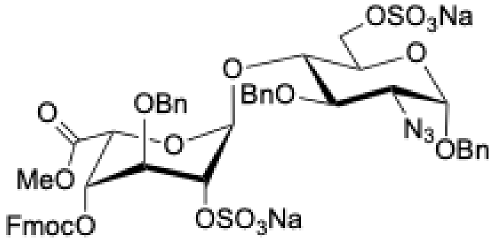

Hydrazine acetate (0.9 g, 0.97 mmol) was added to the solution of **S9** (0.4 g, 0.39 mmol) in a mixture of Toluene/Ethanol (1/2, v/v, 5.0 mL). After 2h, reaction was diluted with EtOAc (10.0 mL) and washed with water (3 x 10.0 mL), dried (Na_2SO_4), and concentrated under vaccum. For acetate deprotection, the residue obtained was taken in MeOH (5.0 mL) and acetyl chloride (0.5 mL) was added dropwise at 0°C. After stirring for 2h at rt, the reaction was concentrated under vaccum and the residue was purified by silica gel column chromatography using a gradient of Hexanes and EtOAc (from 9/1 to 1/9, v/v). The 2’, 6-dihydroxy compound **S13** obtained was dissolved in DMF (1.0 mL) and SO_3.Py complex (20 equiv. per hydroxy) was added. After 16h, completion of sulfation was confirmed by TLC and MS analysis and the reaction was purified by Sephadex LH-20 size exclusion chromatography (SEC) column (2.5

x 120 cm). To sodiate the sulfates, compound was passed through a column of Dowex® 50 x 8 Na+ resin (0.6 x 5 cm) using MeOH as eluant to afford **S14** (0.2 g, 47.2% over 3 steps). ^{1}H NMR (600 MHz, $CDCl_3$): δ 7.87 – 6.97 (m, 23H, *Ar*H), 5.48 (s, 1H, H-1^{B}), 5.01 (d, *J* = 3.6 Hz, 1H, H-1^{A}), 4.87 –4.64 (m, 7H, 2 x C*H*$_2$-Bn, H-5^{B}, H-4^{B} and H-2^{B}), 4.62 – 4.27 (m, 6H, C*H*$_2$-Bn, C*H*$_2$-Fmoc, H-6A^{A} and H-6B^{A}) , 4.24 (t, *J* = 5.8 Hz, 1H, C*H*-Fmoc), 4.18* (d, *J* = 3.0 Hz, 1H, H-3^{B}), 4.11* (td, *J* = 9.5, 3.5 Hz, 1H, H-5^{A}), 3.93 – 3.89 (m, 1H, H-4^{A}), 3.72 (t, *J* = 9.8 Hz, 1H, H-3^{A}), 3.43 – 3.37 (m, 1H, H-2^{A}), 3.01 (s, 3H, COOC*H*$_3$); ^{13}C NMR‡ (151 MHz, $CDCl_3$): δ 129.6 – 120.1 (multiple Ar*C*), 97.1, 96.7, 78.3, 74.2, 72.5, 72.4, 72.2, 71.5, 71.3, 70.0, 69.0, 69.6, 68.6, 65.7, 65.4, 63.4, 51.6 and 46.9 ; HRMS (ESI): calcd. for $C_{49}H_{47}N_3O_{19}S_2$ $[M\text{-}2Na]^{2-}$ 522.6123, found 522.4544.

Benzyl-*O*-(2-*O*-sulfonate-3-*O*-benzyl-α-L-idopyranosyluronate)-(1→4)-*O*-2-deoxy-2-sulfamino-3-*O*-benzyl-6-*O*-sulfonate-α-D-glucopyranoside, sodium salt (**S17**):

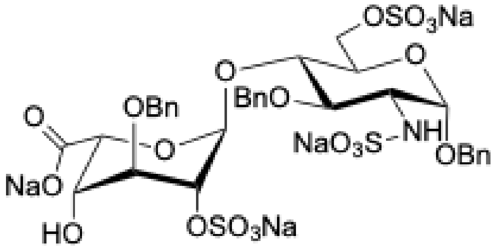

To the stirring solution of **S14** (0.2 g, 0.18 mmol) in THF (5.0 mL) was added a premixed solution of lithium hydroxide (aq. LiOH, 1.0 M) and hydrogen peroxide (aq. H_2O_2, 30 % solution) (3/1, 4.0 mL). After 16h, MeOH (2.0 mL) was added and the pH was adjusted to ~ 14 with 4.0 M NaOH and stirred for another 6h. Upon completion, the pH was adjusted to 8.0–8.5 by careful addition of glacial AcOH, and the mixture was concentrated in vacuo. The residue obtained was purified by reverse phase chromatography (RP-18 silica gel column, H_2O/MeOH, 9/1→1/9, v/v). The appropriate fractions were concentrated in vacuo, and the residue was passed through a column Dowex® 50 x 8 Na^+ resin (0.6 x 5 cm) using MeOH/H_2O (1/1, v/v) as eluent. The appropriate fractions were combined and lyophilized to afford **S15** (0.15 g, 88.2%) as sodium salt. To the solution of azido sugar (**S15**, 0.15 g, 0.19 mmol) in THF (5.0 mL), trimethyl phosphine (PMe_3, 10 equiv., 1.0 M sol in THF) and aq. NaOH solution (0.1 M, 20 equiv.) were added, and the reaction mixture was stirred for 2h. The progress of reaction was monitored by TLC (EtOAc/MeOH/H_2O 7/2/1, v/v/v). Upon completion, the pH was adjusted to 8.0–8.5 by careful addition of glacial AcOH, and the mixture was concentrated in vacuo. The residue obtained was purified by reverse phase chromatography (RP-18 silica gel column, H_2O/MeOH, 9/1→1/9, v/v). The appropriate fractions were concentrated in vacuo, and the residue was passed through a column Dowex®

50 x 8 Na^+ resin (0.6 x 5 cm) using MeOH/H_2O (1/1, v/v) as eluent. The appropriate fractions were combined and lyophilized to afford **S16** (0.14 g, quant.) as sodium salt. The amino sugar (**S16**, 0.14 g, 0.19 mmol) was dissolved in a mixture of MeOH (2.0 mL), Et_3N (0.3 mL) and aq. NaOH solution (0.1 M, 1.0 mL) at 0 °C was added SO_3.Py complex (5.0 equiv.). The progress of reaction was monitored by TLC (EtOAc/pyridine/H_2O/AcOH 8/5/3/1, v/v/v/v). Additional portions of SO_3.Py complex were added at 0 °C after 1h, 2h, 4h and 8h. After stirring for an additional 8h, the reaction mixture was concentrated in vacuo and the residue was passed through a column of Dowex® 50 x 8 Na^+ resin (0.6 x 5 cm) using H_2O/MeOH (9/1, v/v) as eluent to afford desired product. The appropriate fractions were concentrated in vacuo and further was by reverse phase chromatography (RP-18 silica gel column, H_2O/MeOH, 9.5/0.5→1/1, v/v). Appropriate fractions were lyophilized to afford **S17** as sodium salt (0.1 g, 64.1 %). ^{1}H NMR (600 MHz, D_2O): δ 7.47 – 7.18 (m, 15H, *Ar*H), 5.20 (d, *J* = 3.6 Hz, 1H, H-1^A), 5.14 (s, 1H, H-1^B), 4.69 – 4.48 (m, 7H, 3 x C*H*$_2$-Bn and H-5^B), 4.42 (br. s, 1H, H-2^B), 4.19 (dd, *J* = 11.6, 4.3 Hz, 1H, H-6A^A), 4.15 – 4.09 (m, 1H, H-6B^A), 4.05 (t, *J* = 2.7 Hz, 1H, H-4^B), 3.91 – 3.82 (m, 2H, H-3^B and H-5^A), 3.83 (t, *J* = 9.4 Hz, 1H, H-4^A), 3.58 (dd, *J* = 10.5, 9.0 Hz, 1H, H-3^A), 3.35 (dd, *J* = 10.5, 3.6 Hz, 1H, H-2^A); ^{13}C NMR‡ (151 MHz, D_2O): δ 129.2 – 128.1 (multiple Ar*C*), 98.1, 97.1, 76.9, 75.4, 74.7, 74.5, 72.2, 71.4, 70.4, 69.2, 69.2, 67.2, 66.6 and 57.1; HRMS (ESI): calcd. for $C_{33}H_{37}NO_{20}S_3$ $[M+2H-4Na]^{2-}$ 431.5536, found 431.4253.

2-*O*-sulfonate-α-L-idopyranosyluronate-(1→4)-*O*-2-deoxy-2-*N*-sulfamino-6-*O*-sulfonate-α/β-D-glucopyranose, sodium salt (I2S6, **3**):

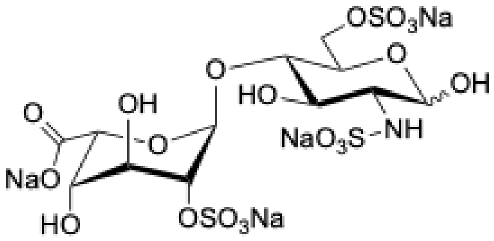

To a stirring solution of disaccharide **S17** (10 mg, 0.1 mmol) in *t*BuOH/H_2O (1/1, v/v, 5.0 mL) at room temperature was added Pd(OH)2/C (10%, 1.5 times the weight of the starting material). The reaction mixture was then placed under an atmosphere of H_2 gas (ballon at atmospheric pressure) and repeated cycle of vaccuming and H_2 gas flushing (3-4 times) were performed. After 16h, the reaction mixture was filtered through PTFE syringe filter, concentrated under vaccum and lyophilized. The residue obtained was disolved in minimum volume of H_2O (~0.2 mL) and passed through a column of Dowex® 50 x 8 Na+ resin (0.6 x 5 cm) using H_2O as eluant. Appropriate fractions were concentrated under vaccum to minimum volume (~0.2 mL) and loaded on to a Bio-Gel P-2 size exclusion chromatography (SEC) column (1.5 x 80 cm). The compound was eluted using H_2O as eluant and desired

fractions (checked with spotTLC, stained with Hanessian's stain) were combined and lyophilized to afford **3** (I2S6, 6.6 mg, 91.6 %) as white fluffy solid (reducing end GlcN α/β mixture, only α-anomer is reported). ^{1}H NMR (600 MHz, D_2O): δ 5.41 (br. s, 1H, H-1^{A}), 5.08 (s, 1H, H-1^{B}), 4.67 (merged with HDO, s, 1H, H-5^{B}), 4.30 – 4.15 (m, 3H, H-6A^{A}, H-6B^{A} and H-2^{B}), 4.11 – 4.02 (m, 1H, H-5^{A}), 3.96 (br. s, 1H, H-3^{B}), 3.91 (br. s, 1H, H-4^{B}), 3.72 – 3.62 (m, 2H, H-3^{A} and H-4^{A}), 3.23 – 3.15 (m, 1H, H-2^{A}); ^{13}C NMR (151 MHz, D_2O): δ 176.1, 99.2, 91.0, 77.6, 75.0, 69.6, 69.5, 69.4, 69.4, 68.4, 66.9 and 58.0.

Monosaccharide standard **H6** (**4**) was prepared from glucosamine (**Scheme S4**), detailed synthesis will be reported elsewhere.

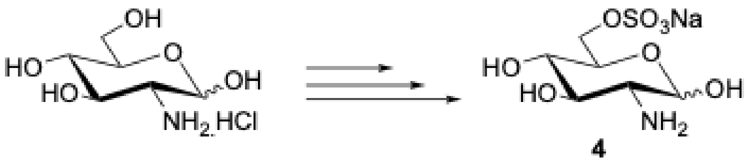

Scheme S4. Chemical synthesis of monosaccharide standard H6 (**4**)

2-Deoxy-2-amino-6-*O*-sulfonate-α/β-D-glucopyranose, sodium salt (H6, **4**):

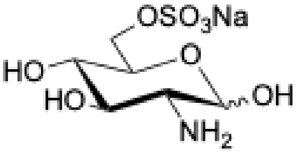

Monosaccharide **4** was obtained as white fluffy solid. ^{1}H NMR (600 MHz, D_2O): δ 5.35 (d, J = 3.6 Hz, 1H, H-1), 4.31 – 4.12 (m, 2H, H-6A and H-6B), 4.05 – 3.97 (m, 1H, H-5), 3.81 (dd, J = 10.6, 9.1 Hz, 1H, H-3), 3.49 – 3.44 (m, 1H, H-4) and 3.22 (dd, J = 10.6, 3.6 Hz, 1H, H-2); ^{13}C NMR‡ (151 MHz, D_2O): δ 89.2, 69.6, 69.5, 69.1 66.9 and 54.2; HRMS (ESI): calcd. for $C_6H_{12}NO_8$ $[M]^{1-}$ 258.0284, found 257.9351.

References:

1) S. Arungundram, K. Al-Mafraji, J. Asong, F. E. Leach, I. J. Amster, A. Venot, J. E. Turnbull, G.-J. Boons, *J. Am. Chem. Soc.* **2009**, *131*, 17394-17405.

2) C. Zong, A. Venot, X. Li, W. Lu, W. Xiao, J. L. Wilkes, C. L. Salanga, T. M. Handel, L. Wang, M. A. Wolfert, G. J. Boons, *J. Am. Chem. Soc.* **2017**, *139*, 9534-9543.

3) P. Chopra, A. Joshi, J. Wu, W. Lu, T. Yadavalli, M. A. Wolfert, D. Shukla, J. Zaia, G.-J. Boons, *Proc. Natl. Acad. Sci. U. S. A.* **2021**, *118*, e2012935118.

4) R. Karlsson, P. Chopra, A. Joshi, Z. Yang, S. Y. Vakhrushev, T. M. Clausen, C. D. Painter, G. P. Szekeres, T. -H. Chen, D. R. Sandoval, et al. *Sci. Adv.* **2021**, 7 (52), eabl6026.

Scanned spectras.

Benzyl-*O*-(methyl-2-*O*-acetyl-3-*O*-benzyl-4-*O*-[acetyl]-β-D-glucopyranosyluronate)-(1→4)-*O*-2-azido-2-deoxy-3-*O*-napthylmethyl-6-*O*-levulinoyl-α-D-glucopyranoside (**S3**)

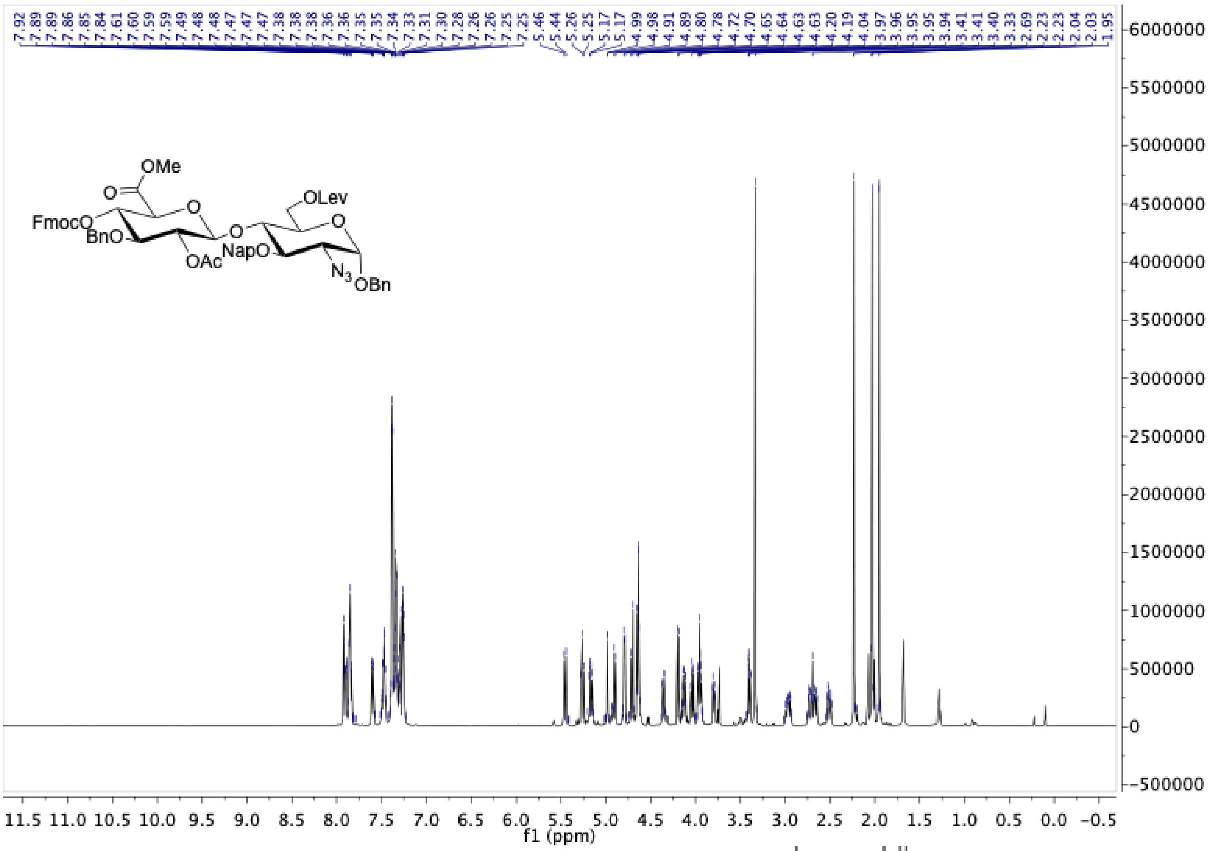

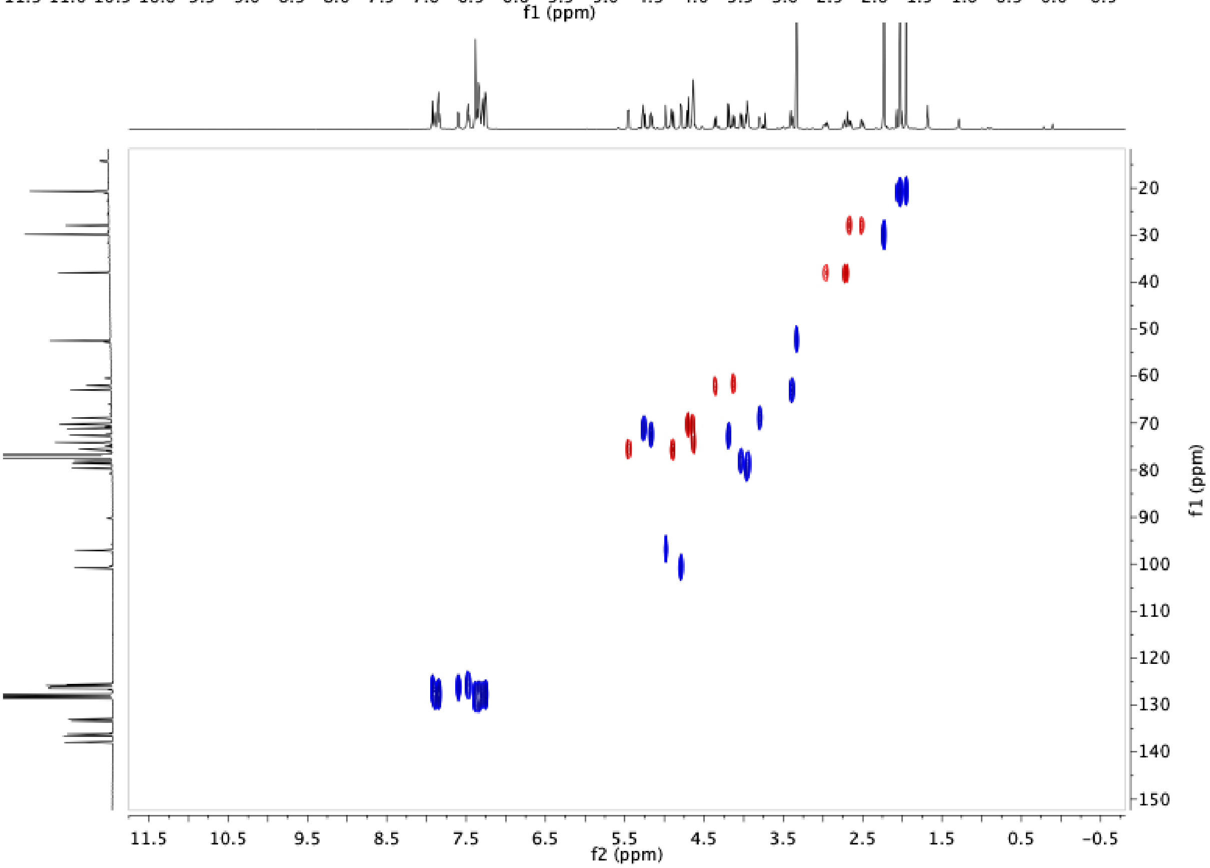

Benzyl-*O*-(3-*O*-benzyl-β-D-glucopyranosyluronate)-(1→4)-*O*-2-azido-2-deoxy-3-*O*-napthylmethyl-α-D-glucopyranoside, sodium salt (**S4**)

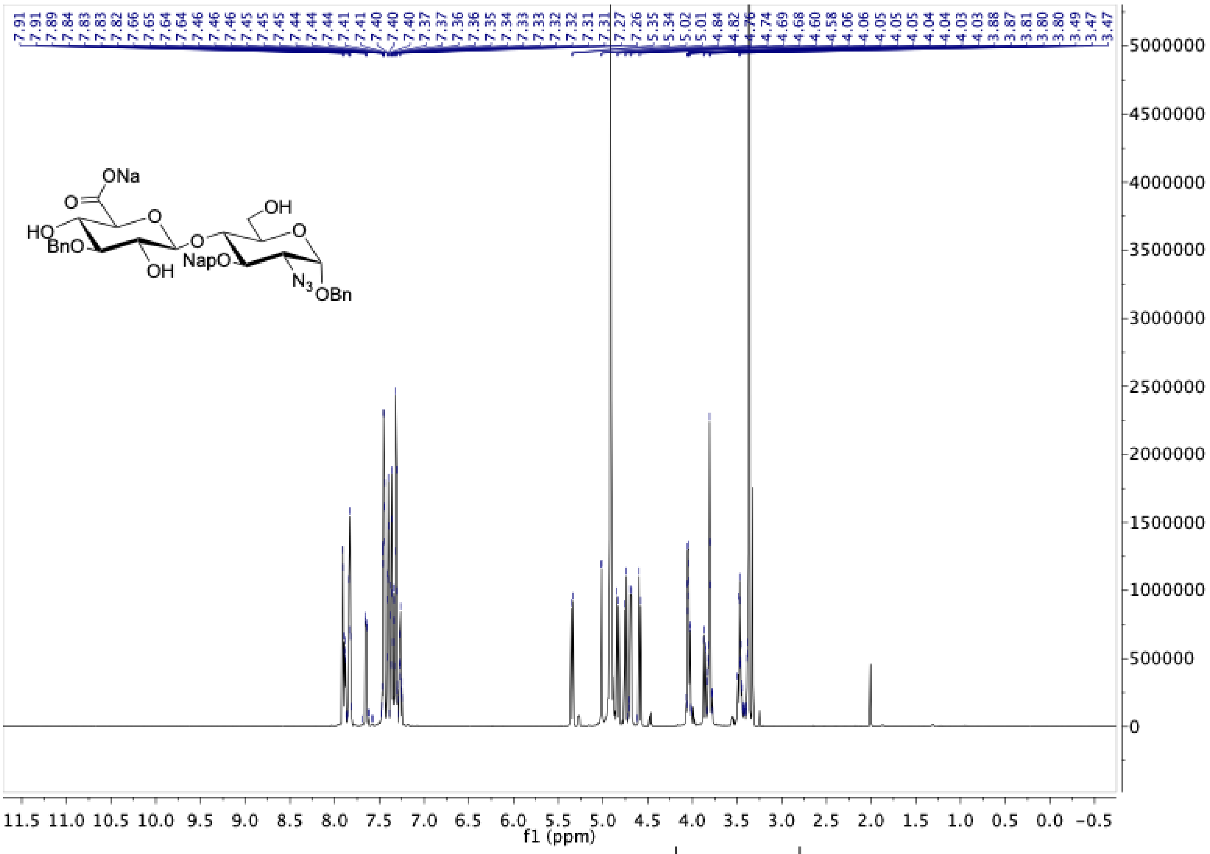

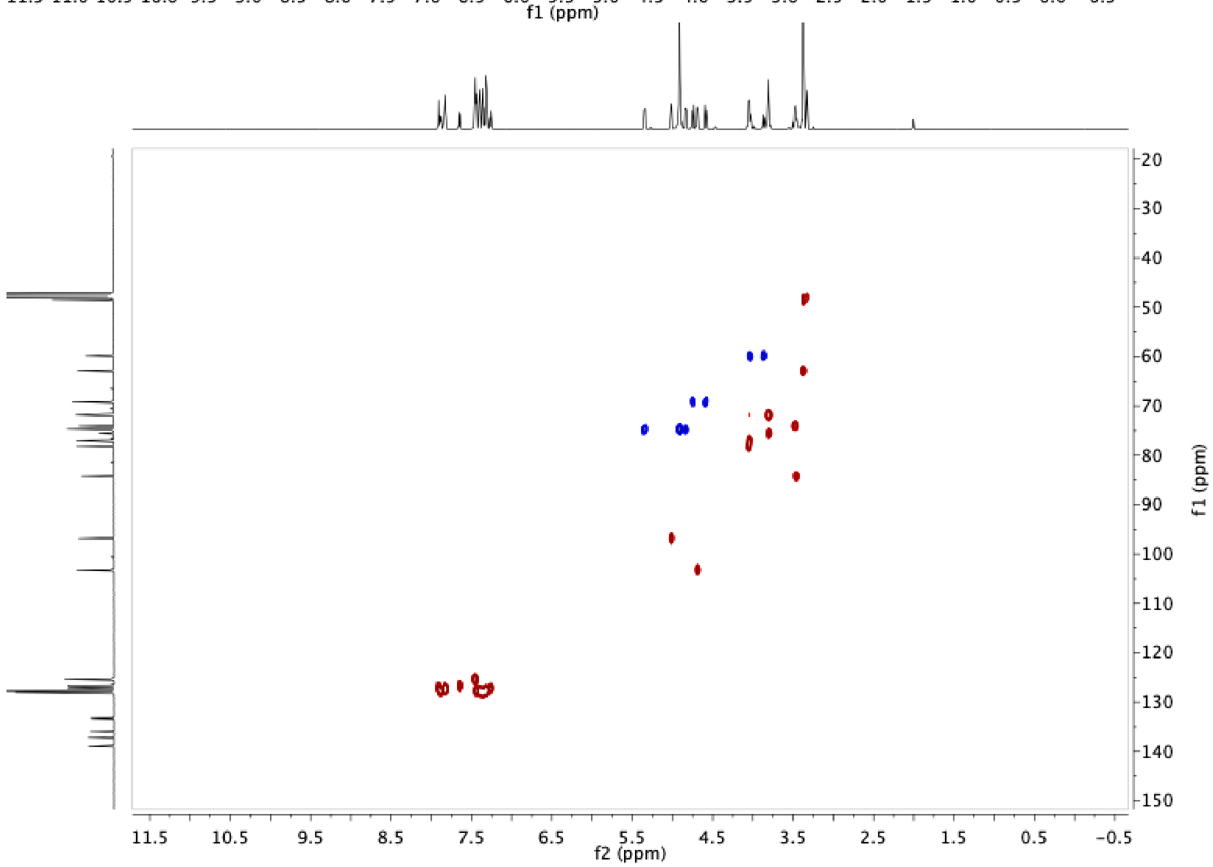

Benzyl-*O*-(3-*O*-benzyl-β-D-glucopyranosyluronate)-(1→4)-*O*-2-deoxy-2-sulfamino-3-*O*-napthylmethyl-α-D-glucopyranoside, sodium salt (**S6**)

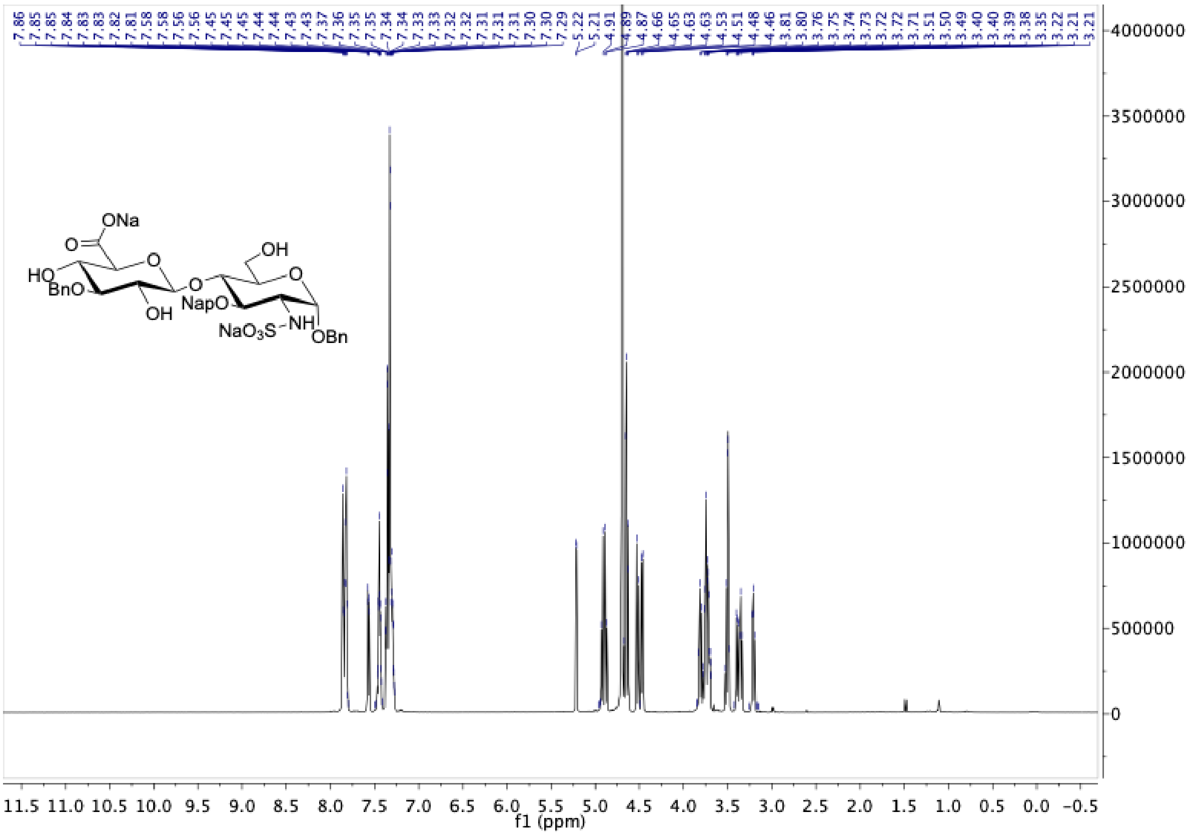

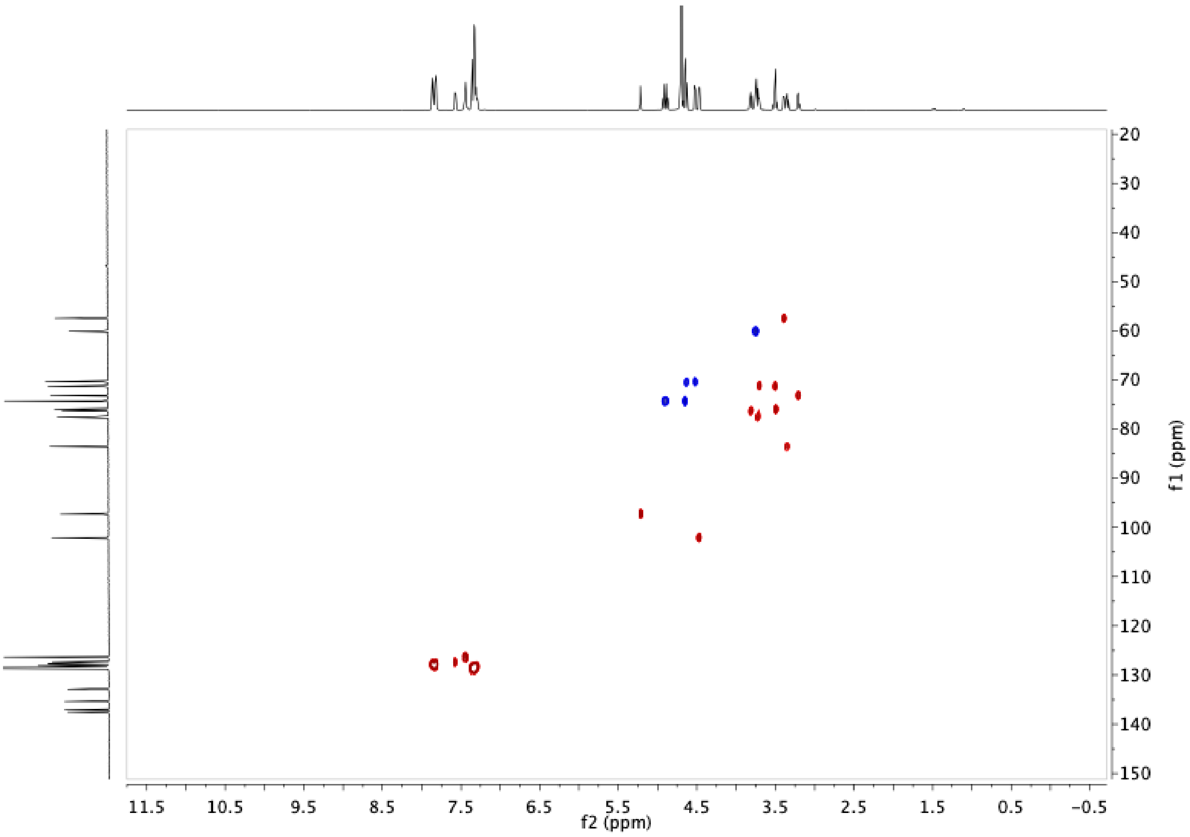

β-D-glucopyranosyluronate-(1→4)-*O*-2-deoxy-2-*N*-sulfamino-α/β-D-glucopyranose, sodium salt (G0S0, **1**)

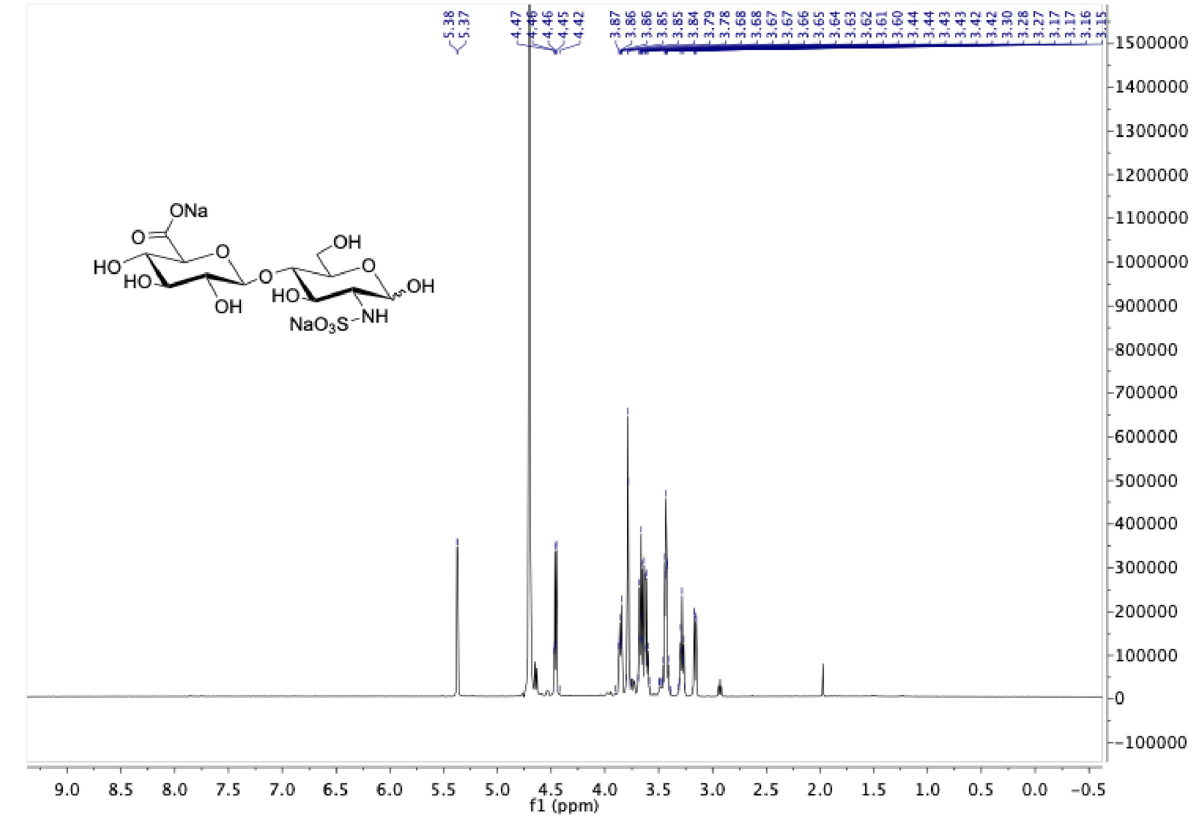

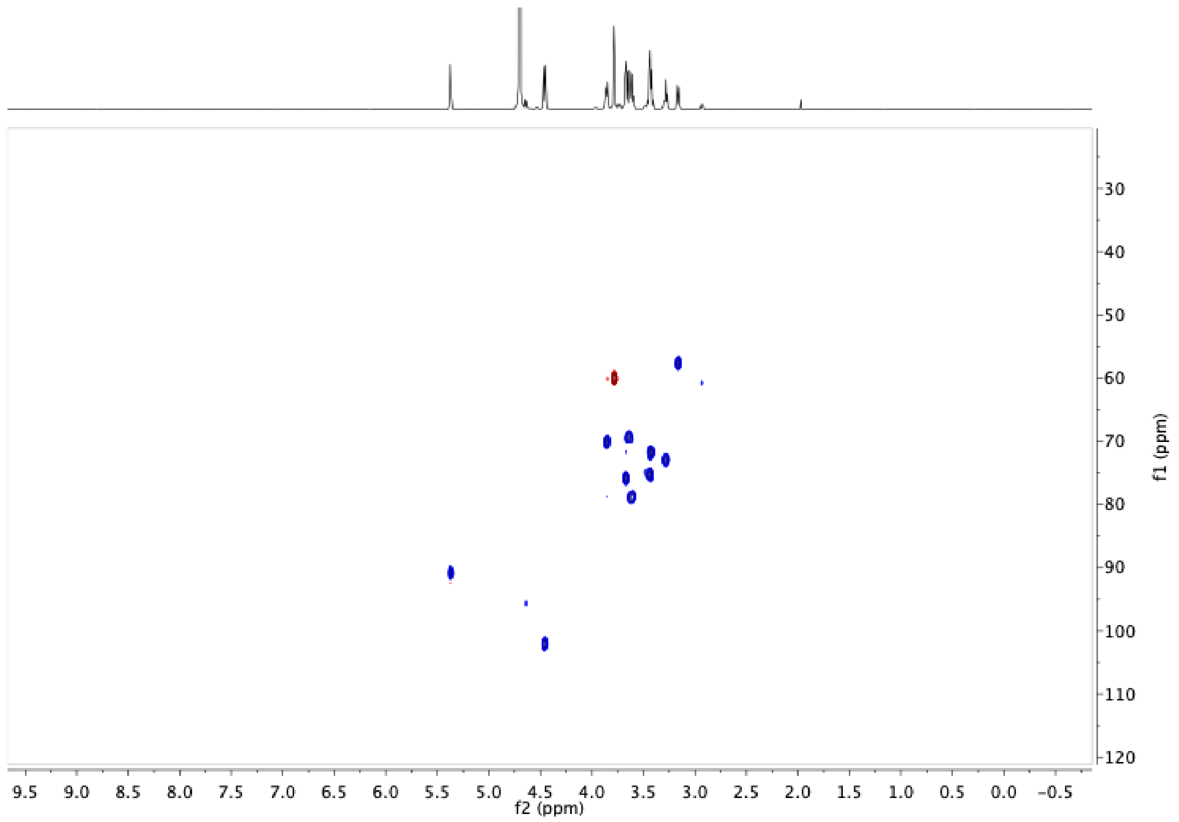

Benzyl-*O*-(methyl-2-*O*-levulinoyl-3-*O*-benzyl-4-*O*-[9-fluorenylmethyloxycarbonyl]-α-L-idopyranosyluronate)-(1→4)-*O*-2-azido-2-deoxy-3-*O*-benzyl-6-*O*-acetyl-α-D-glucopyranoside (**S9**)

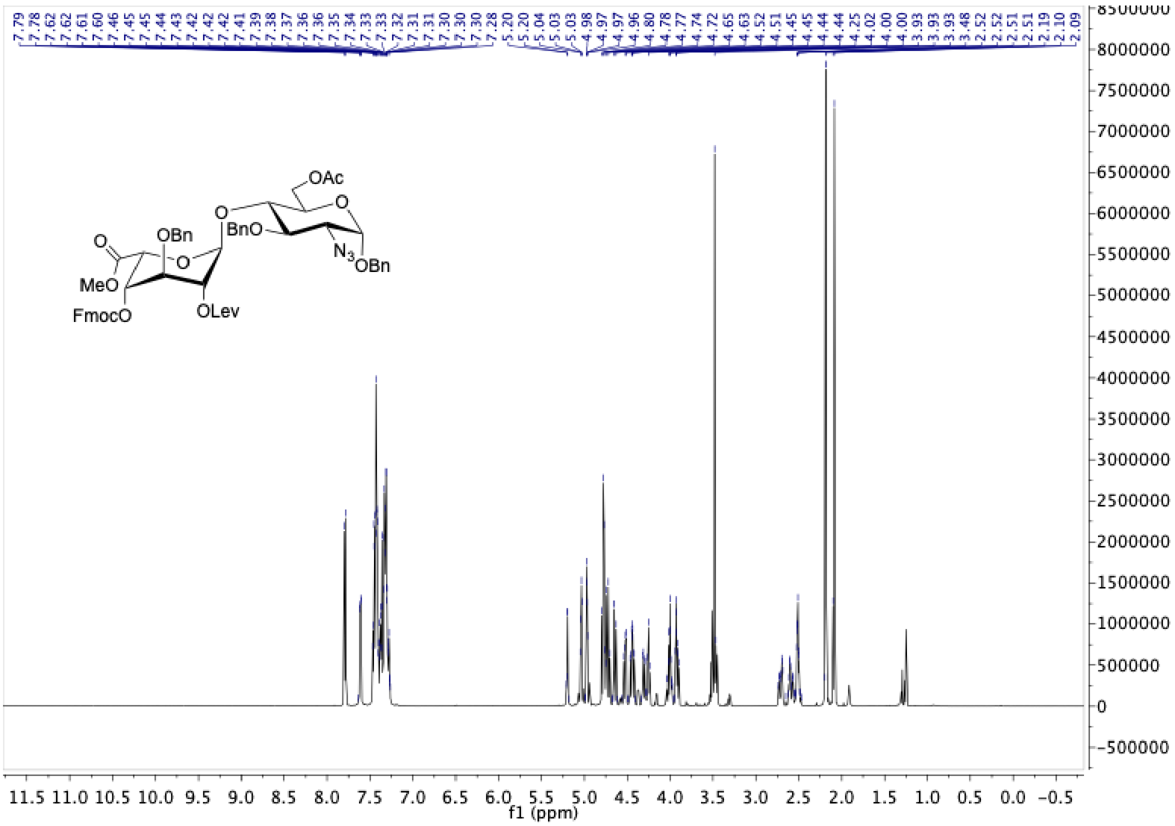

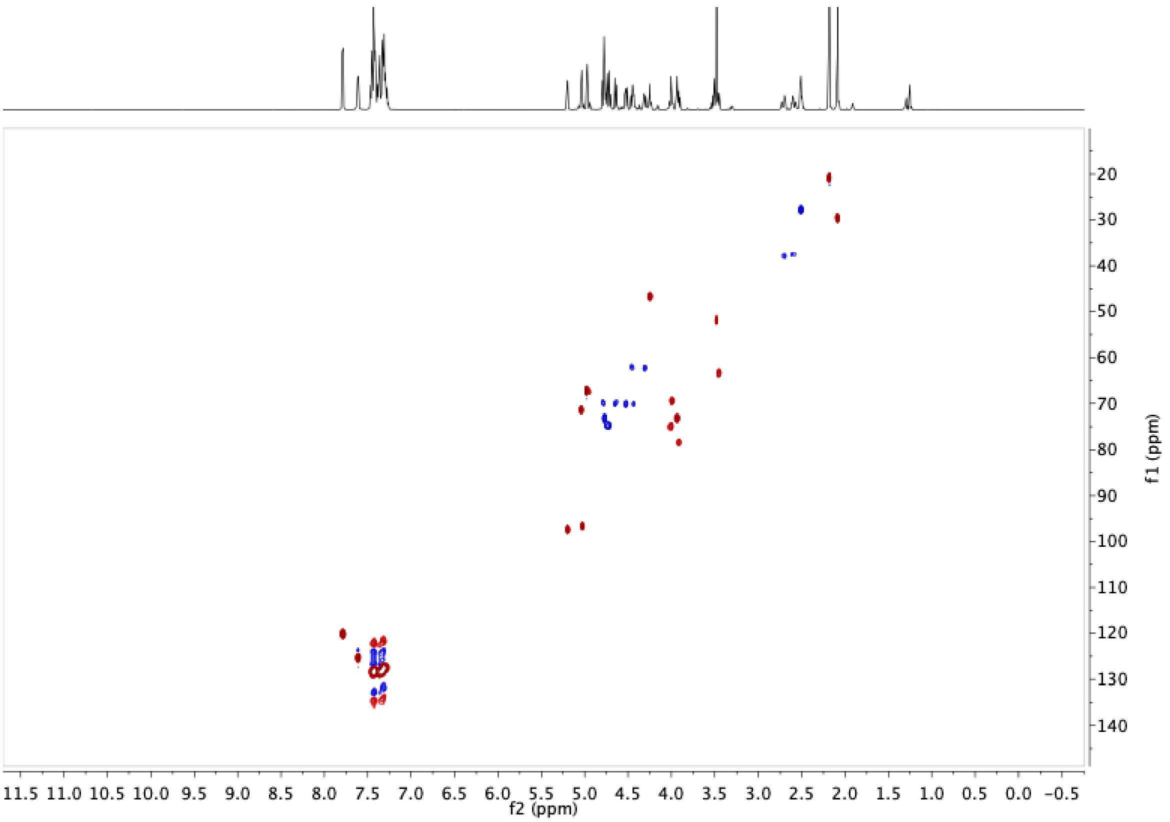

Benzyl-*O*-(3-*O*-benzyl-α-L-idopyranosyluronate)-(1→4)-*O*-2-azido-2-deoxy-3-*O*-benzyl-α-D-glucopyranoside, sodium salt (**S10**)

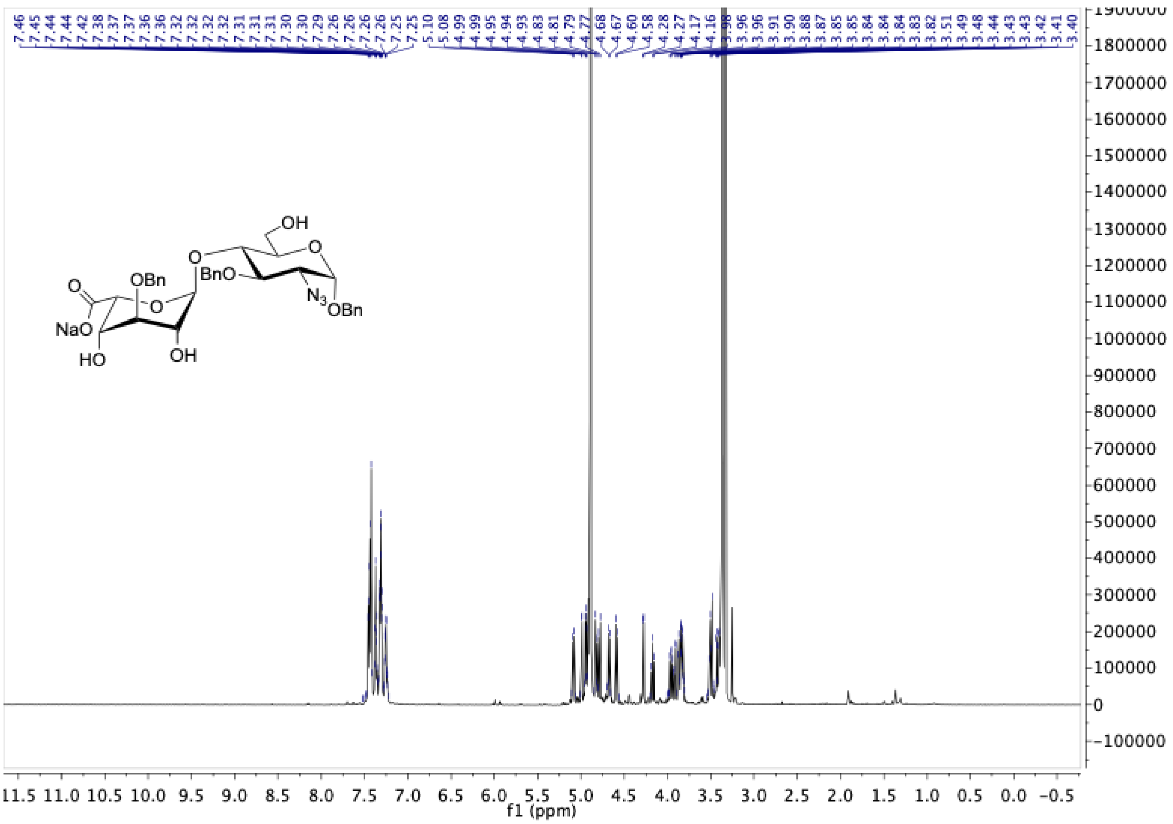

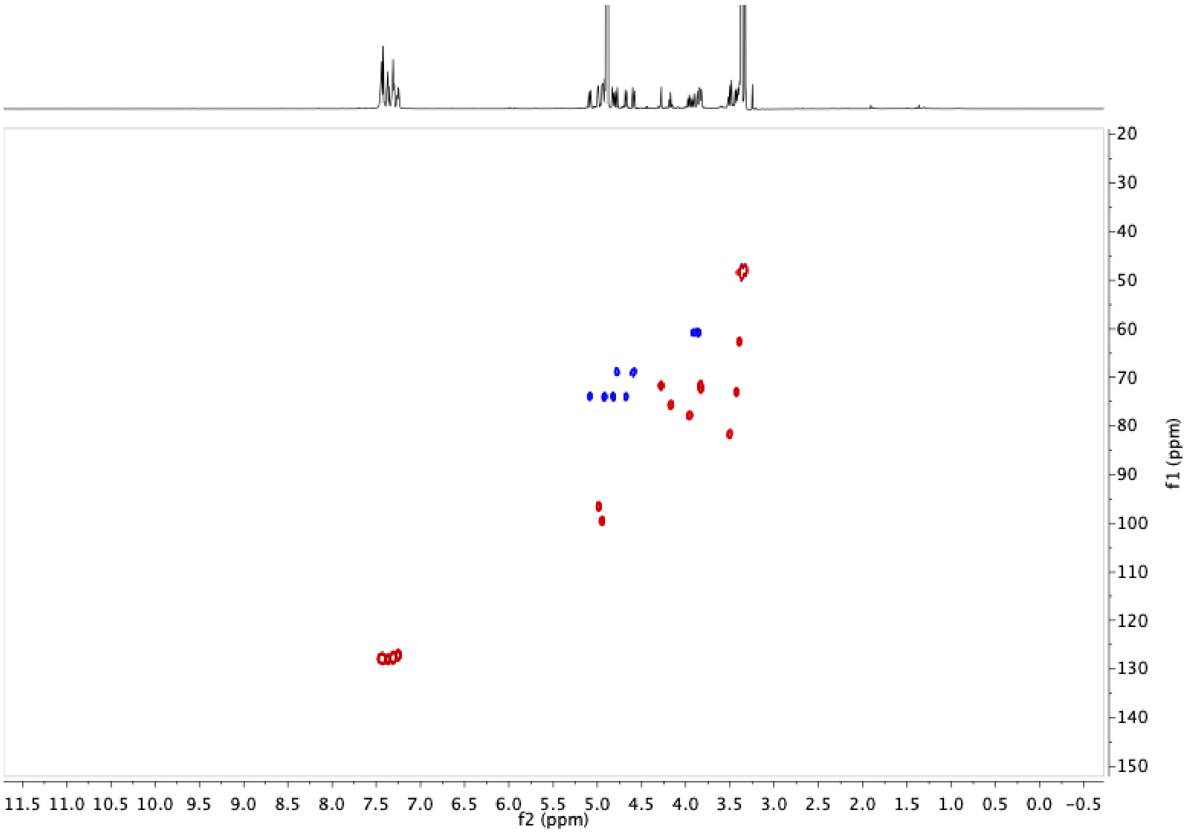

Benzyl-*O*-(3-*O*-benzyl-α-L-idopyranosyluronate)-(1→4)-*O*-2-deoxy-2-sulfamino-3-*O*-benzyl-α-D-glucopyranoside, sodium salt (**S12**)

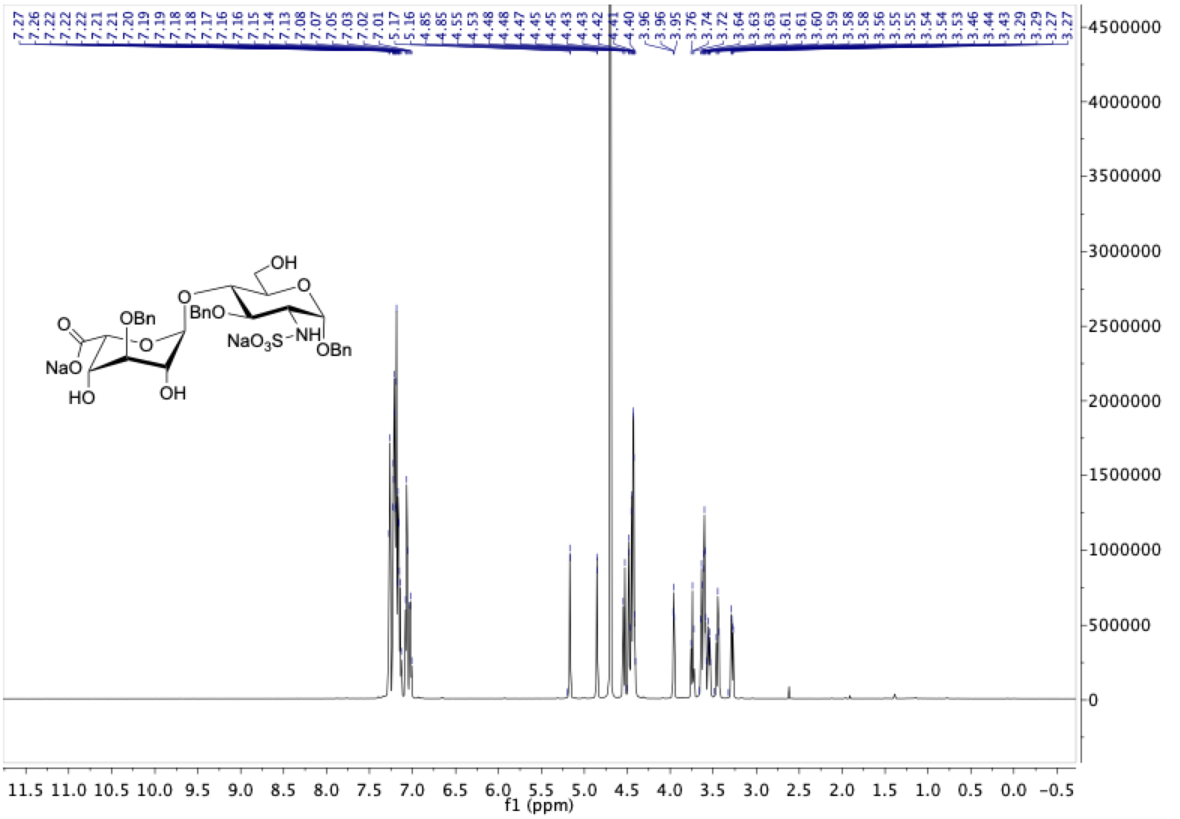

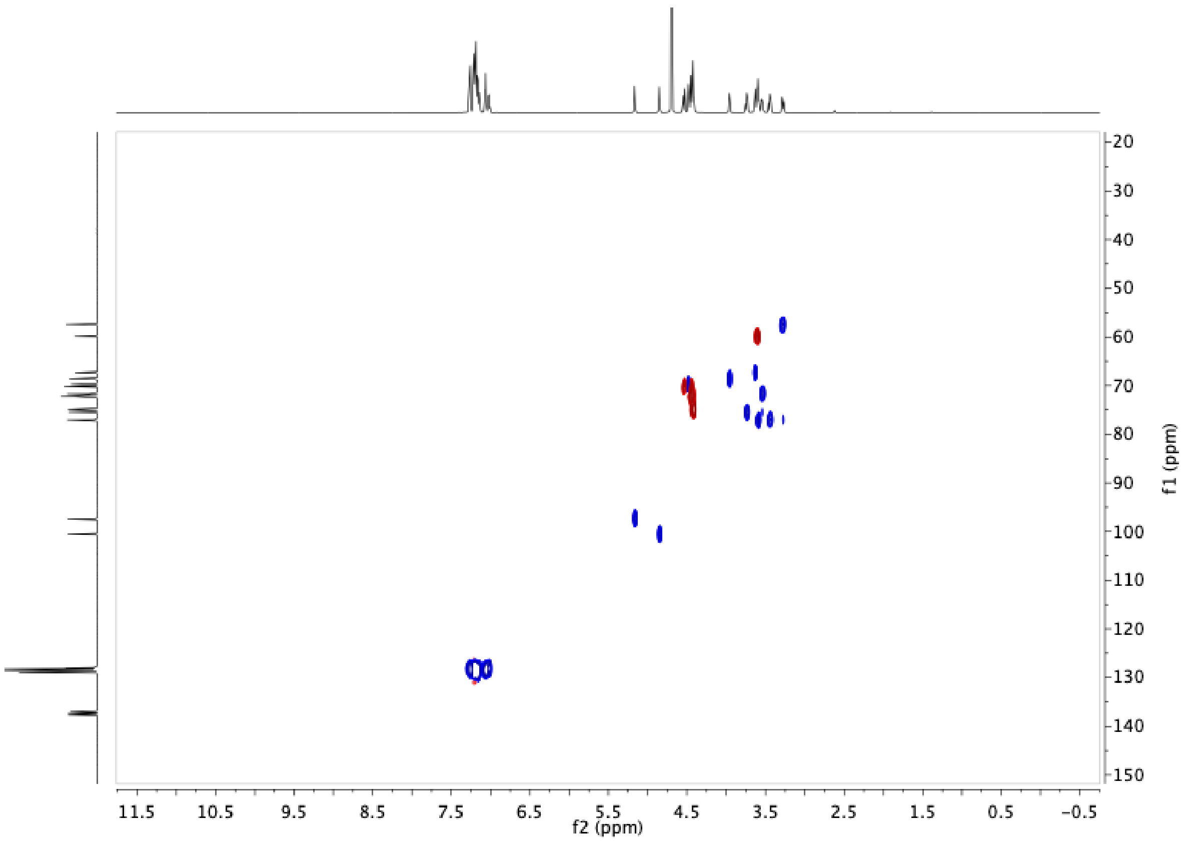

α-L-idopyranosyluronate-(1→4)-*O*-2-deoxy-2-*N*-sulfamino-α/β-D-glucopyranose, sodium salt (I0S0, **2**)

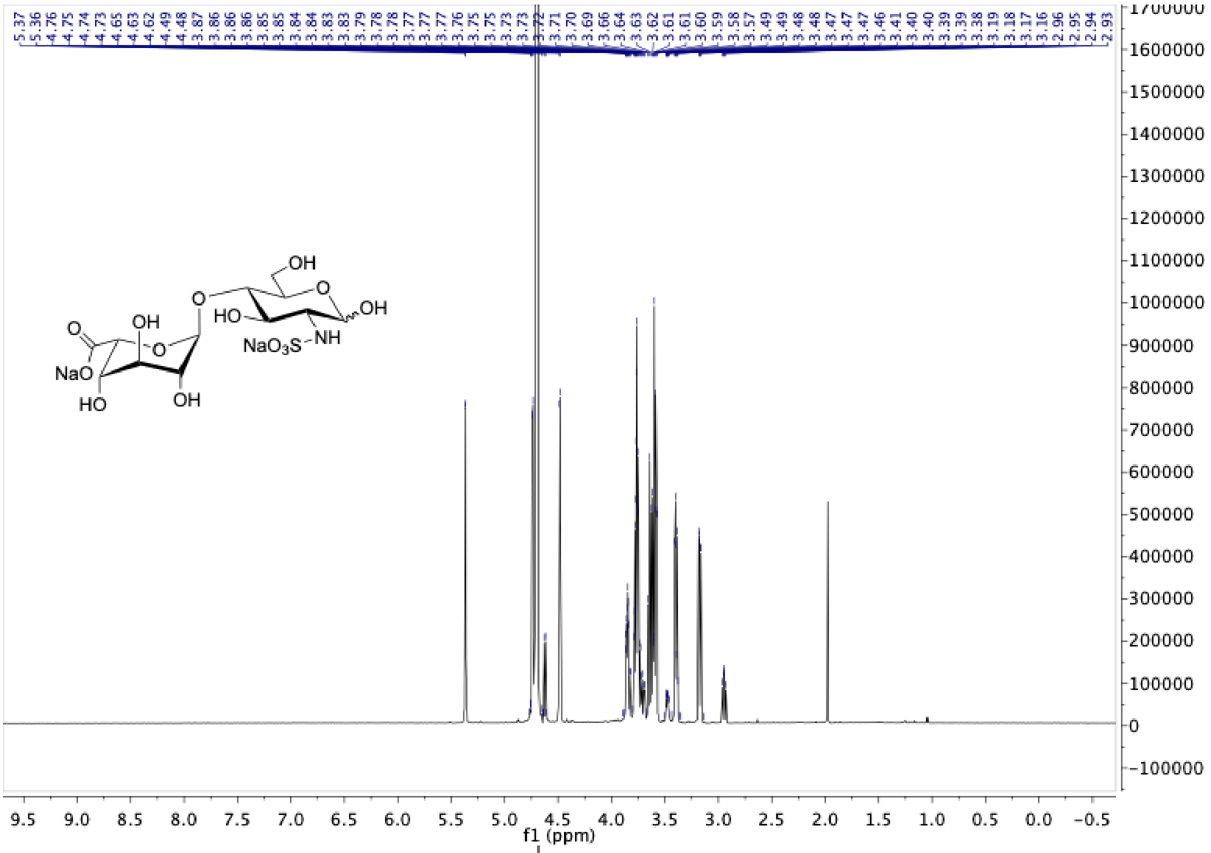

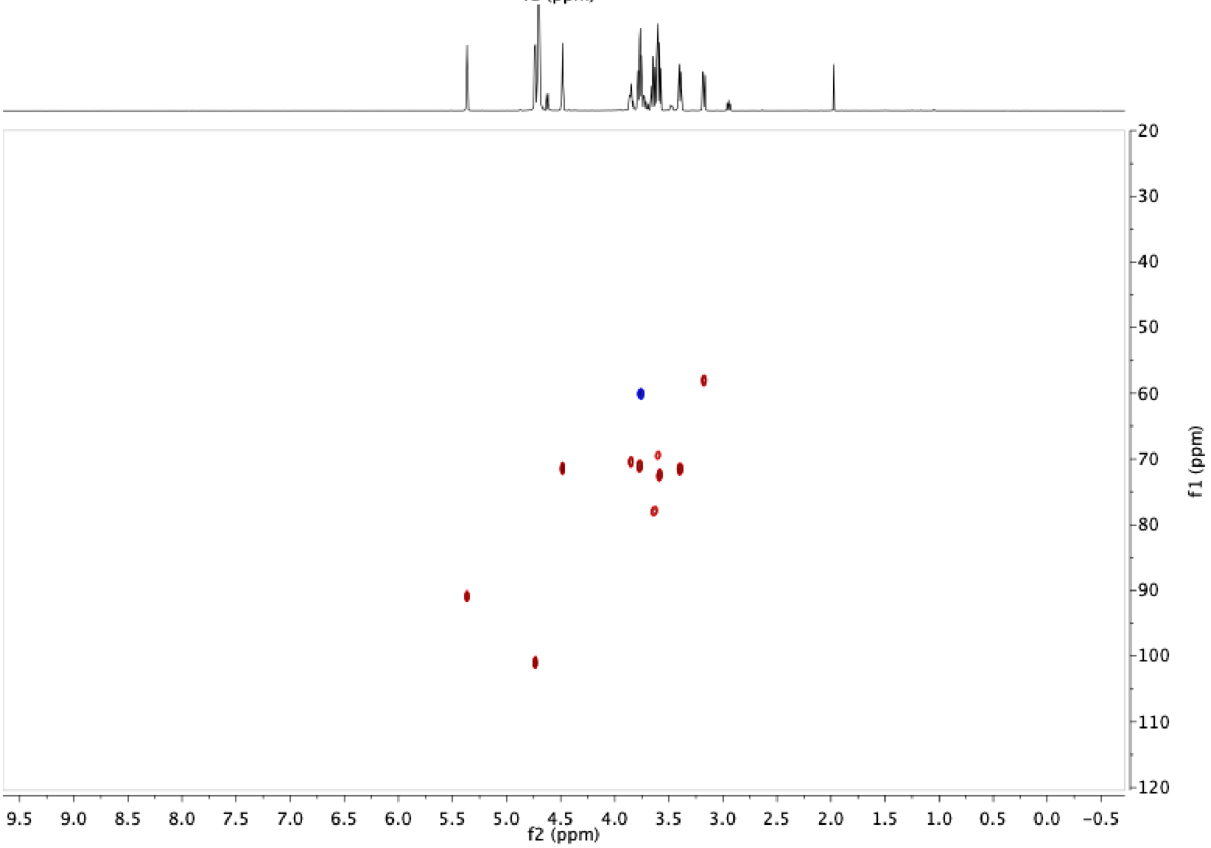

Benzyl-*O*-(2-*O*-sulfonate-3-*O*-benzyl-4-*O*-[9-fluorenylmethyloxycarbonyl]-α-L-idopyranosyl uronate)-(1→4)-*O*-2-azido-2-deoxy-3-*O*-benzyl-α-D-glucopyranoside, sodium salt (**S14**):

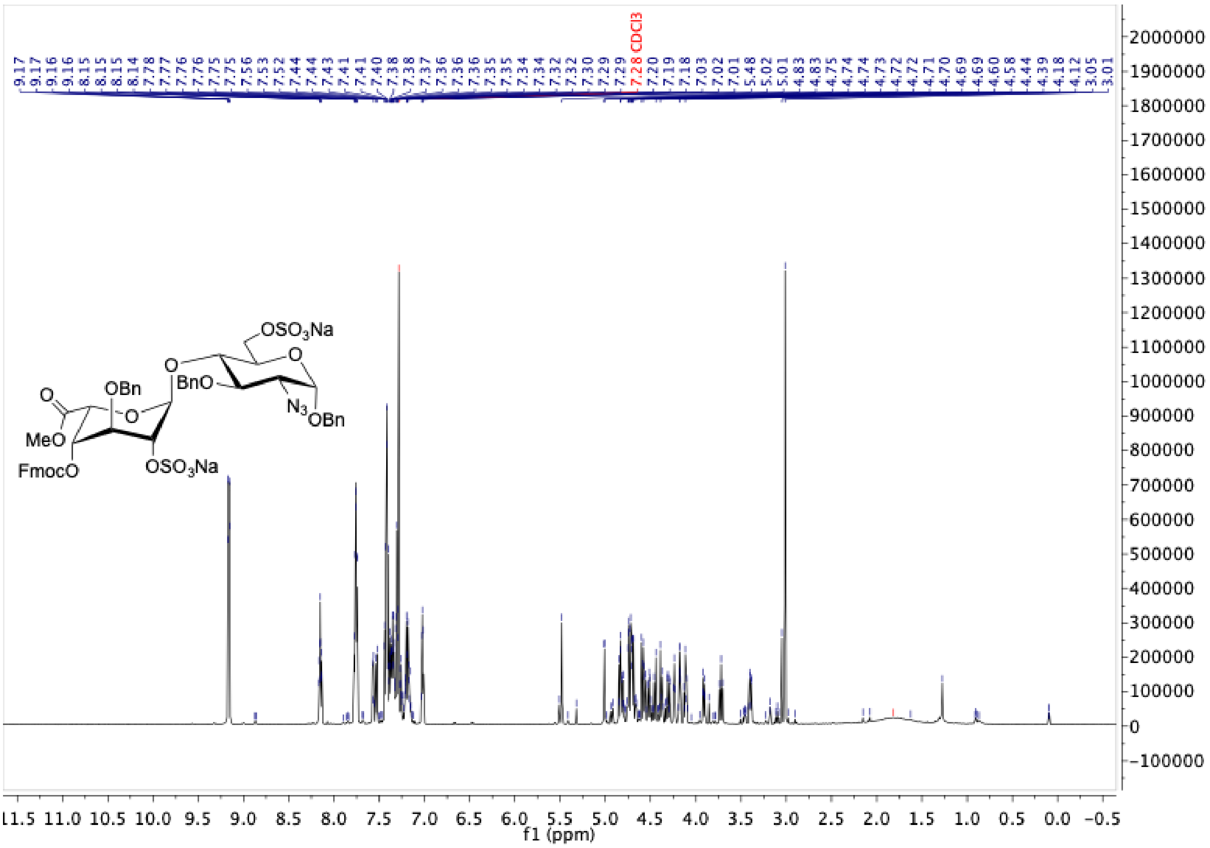

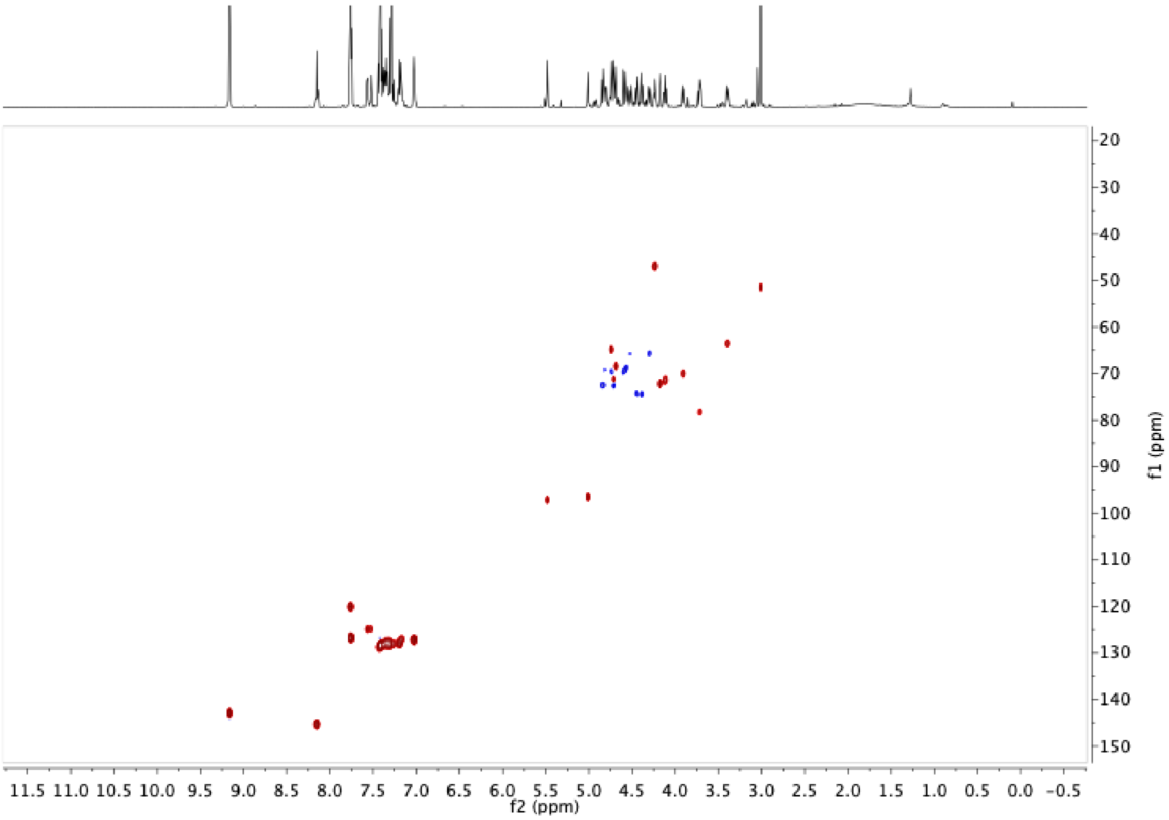

Benzyl-*O*-(2-*O*-sulfonate-3-*O*-benzyl-α-L-idopyranosyluronate)-(1→4)-*O*-2-deoxy-2-sulfamino-3-*O*-benzyl-6-*O*-sulfonate-α-D-glucopyranoside, sodium salt (**S17**)

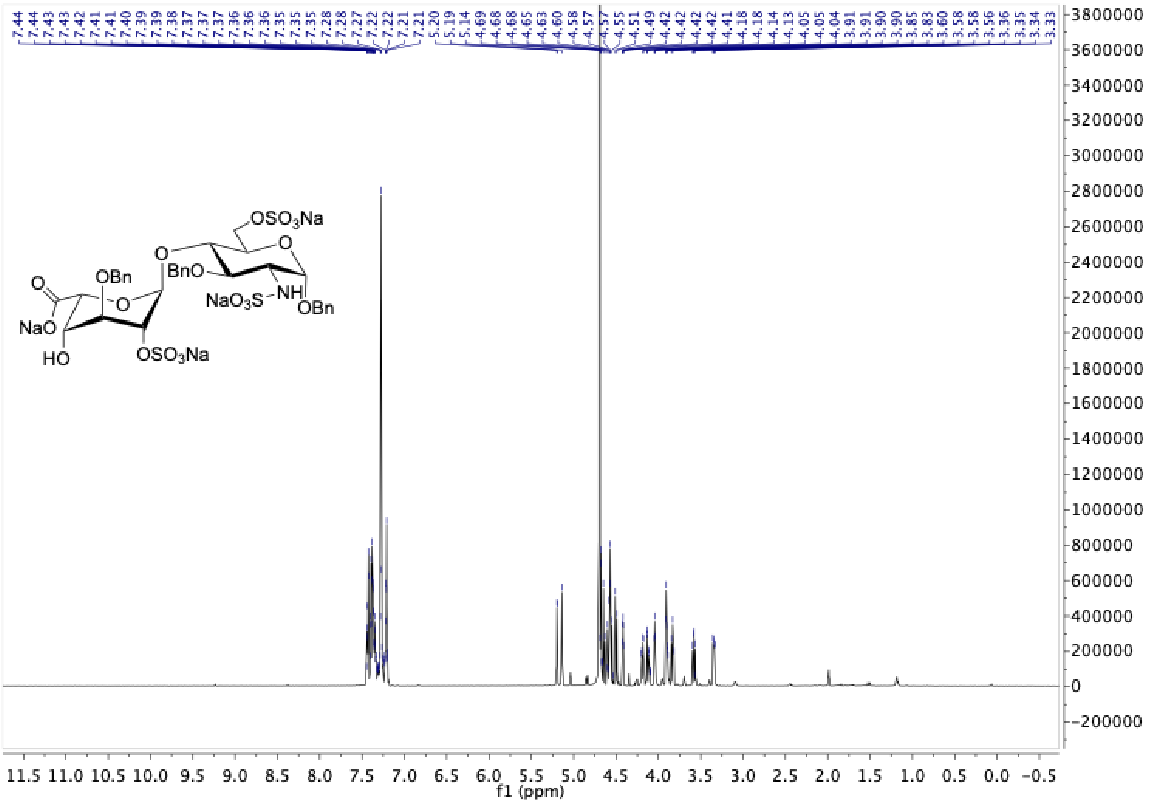

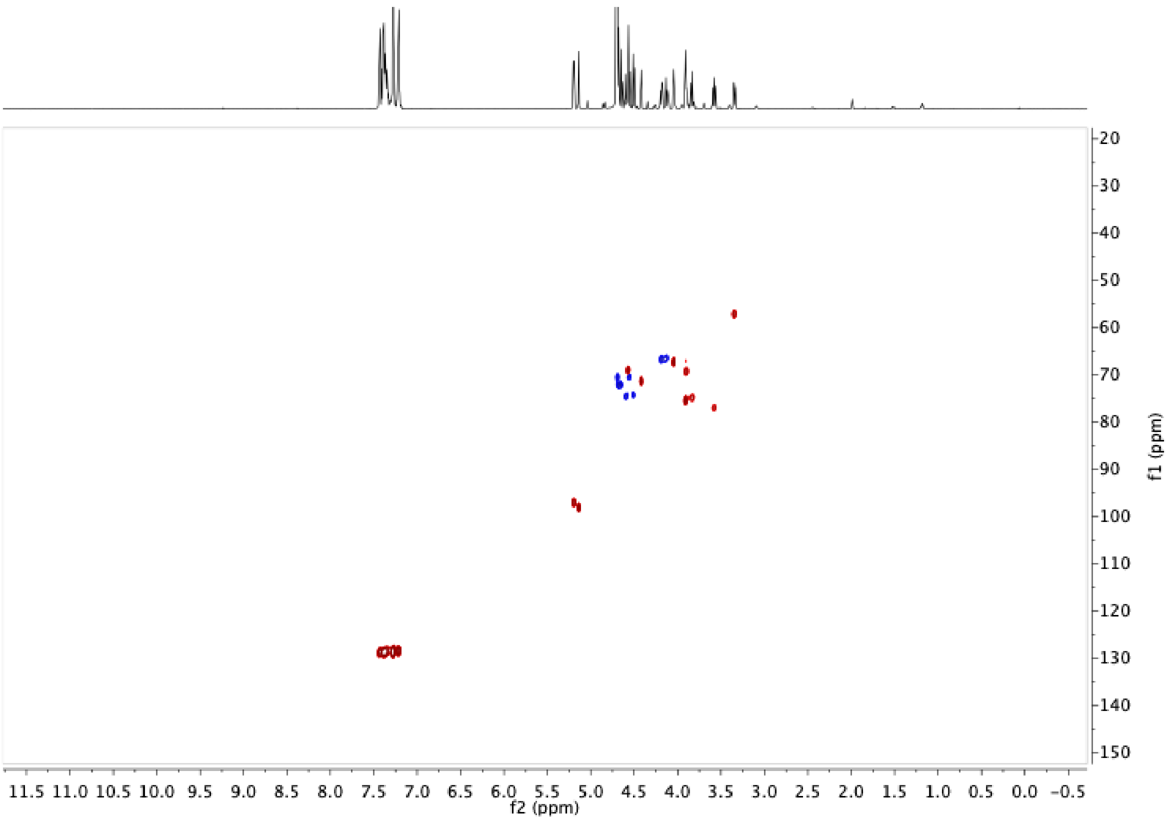

2-*O*-sulfonate-α-L-idopyranosyluronate-(1→4)-*O*-2-deoxy-2-*N*-sulfamino-6-*O*-sulfonate-α/β-D-glucopyranose, sodium salt (I2S6, **3**)

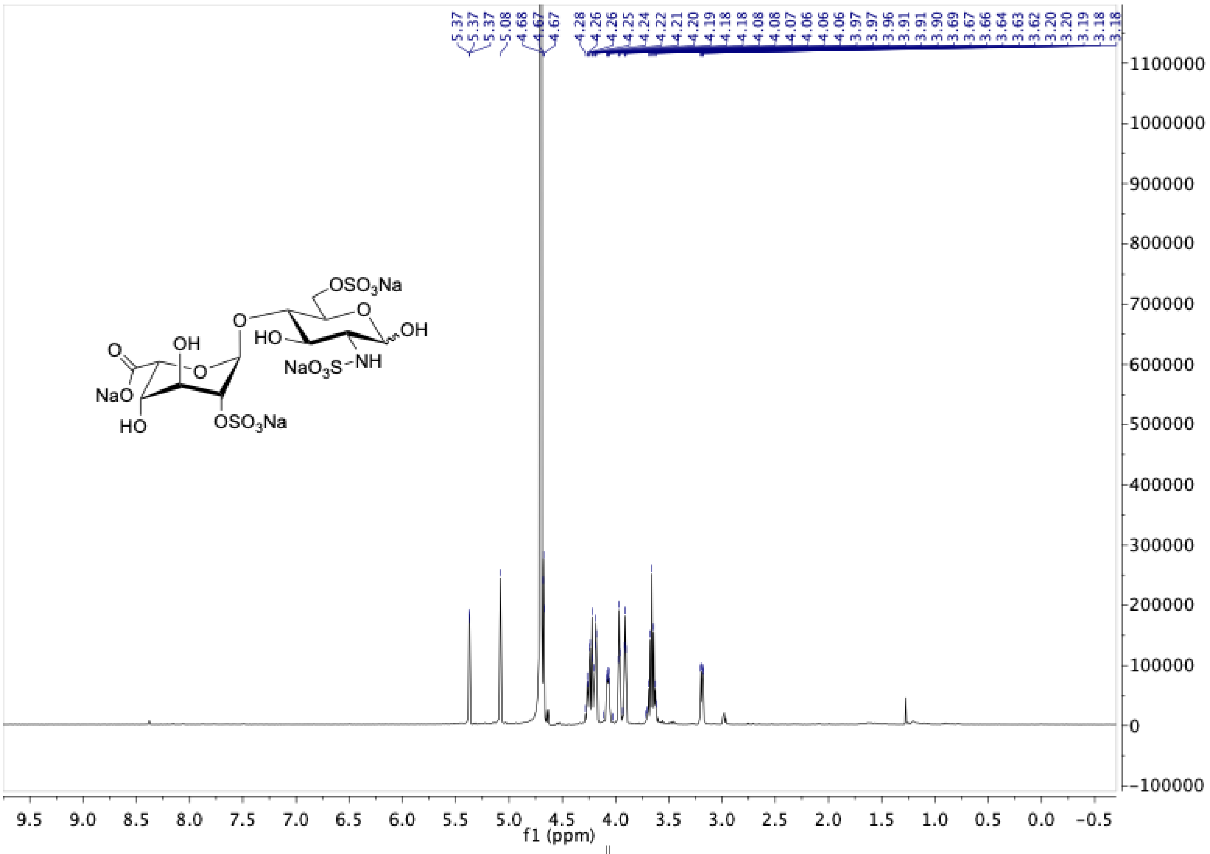

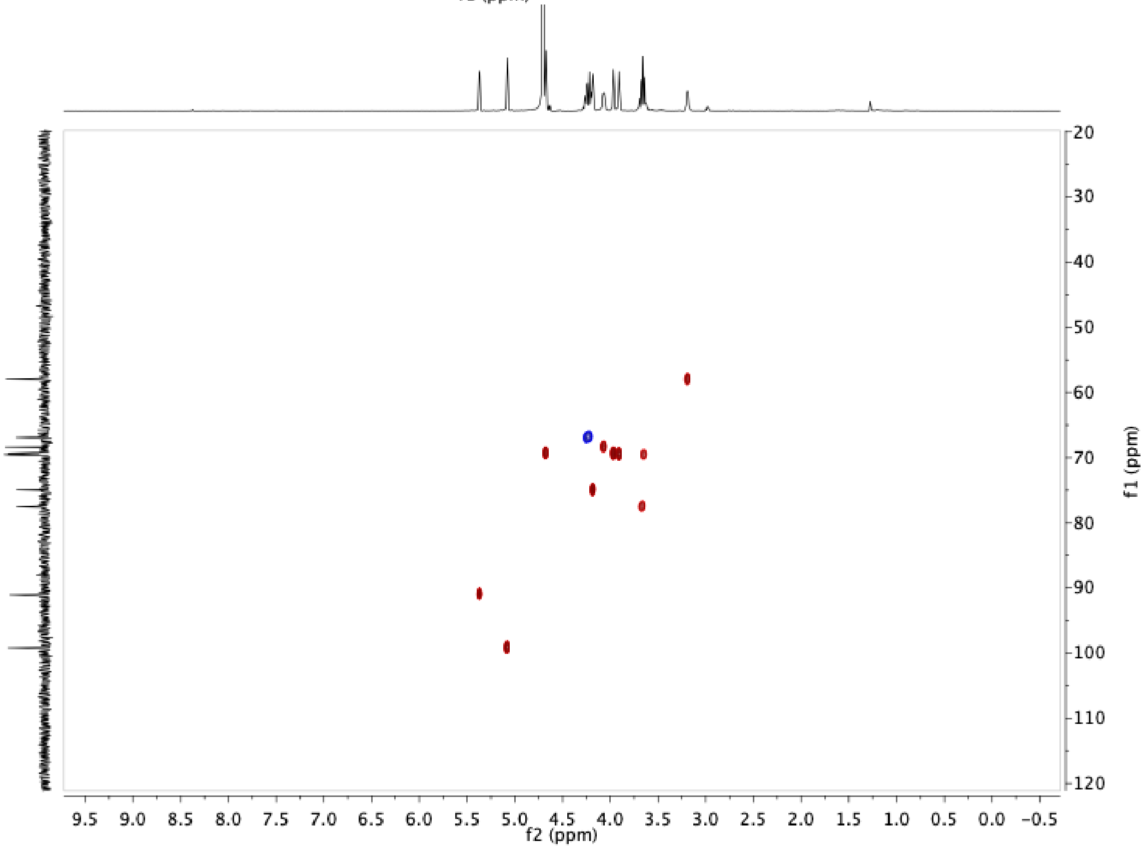

2-Deoxy-2-amino-6-*O*-sulfonate-a/β-D-glucopyranose, sodium salt (H6, **4**)

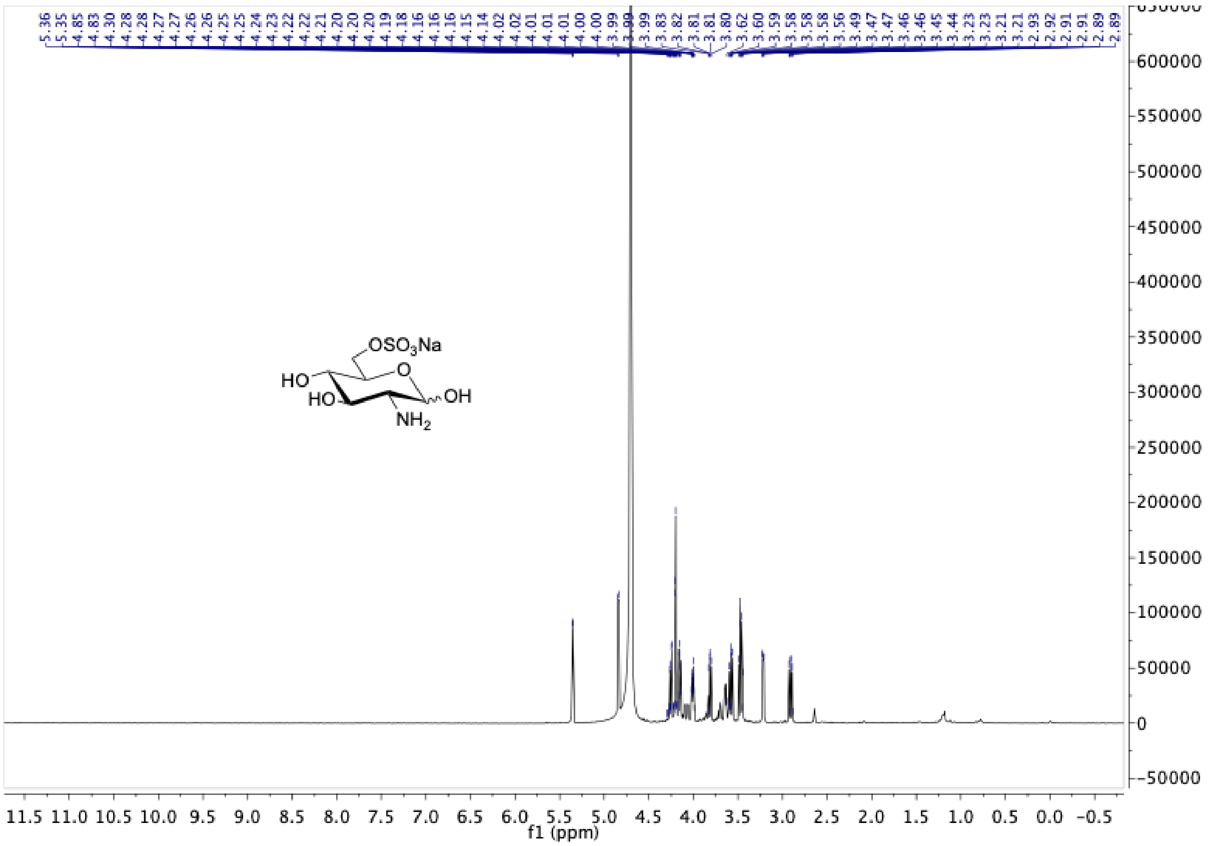

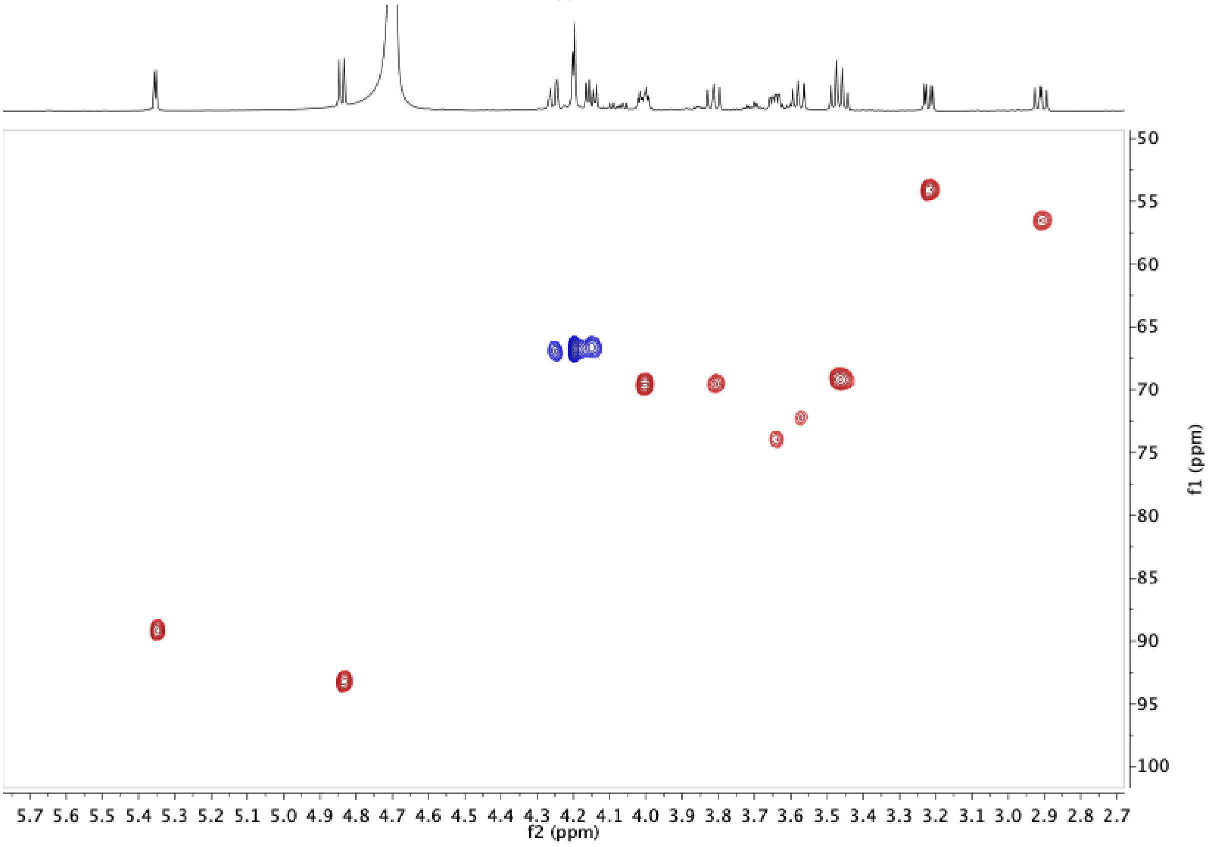

Supplement: Supplementary file 1 [file ac5c02338_si_001.pdf]
